# Psilocybin-assisted therapy for relapse prevention in alcohol use disorder: a phase 2 randomized clinical trial

**DOI:** 10.1016/j.eclinm.2025.103149

**Published:** 2025-03-14

**Authors:** Nathalie M. Rieser, Raoul Bitar, Simon Halm, Christina Rossgoderer, Ladina P. Gubser, Maeva Thévenaz, Yara Kreis, Robin von Rotz, Carlos Nordt, Monika Visentini, Flora Moujaes, Etna J.E. Engeli, Andres Ort, Erich Seifritz, Franz X. Vollenweider, Marcus Herdener, Katrin H. Preller

**Affiliations:** a Department of Adult Psychiatry and Psychotherapy, Psychiatric University Clinic Zurich and University of Zurich, Lenggstrasse 31, Zurich 8032, Switzerland

**Keywords:** Psychedelic-assisted therapy, Alcohol use disorder, Addiction, RCT, Efficacy

## Abstract

**Background:**

Despite the promising therapeutic effects of psilocybin, its efficacy in preventing relapse after withdrawal treatment for alcohol use disorder (AUD) remains unknown. This study aims to assess whether a single dose of psilocybin combined with brief psychotherapy could reduce relapse rates and alcohol use in AUD patients.

**Methods:**

This single-center, double-blind, randomized clinical trial was conducted in Switzerland. We recruited participants with AUD between June 8, 2020, and August 16, 2023 who completed withdrawal treatment within six weeks prior to enrollment. Participants were randomized (1:1) to receive either a single oral dose of psilocybin (25 mg) or placebo (mannitol), combined with brief psychotherapy. The primary outcomes were abstinence and mean alcohol use at 4-week follow-up. Participants completed the timeline followback to assess daily alcohol use. The trial is registered on ClinicalTrials.gov (NCT04141501).

**Findings:**

We included 37 participants who completed the 4-week follow-up (female:male = 14:23; psilocybin = 18, placebo = 19) in the analysis. There were no significant differences between groups in abstinence duration (*p* = 0.55, psilocybin mean = 16.80 days, 95% CI: 14.31–19.29; placebo mean = 13.80 days, 95% CI: 10.97–16.63; Cohen’s *d* = 0.151) or mean alcohol use per day (*p* = 0.51, psilocybin: median = 0.48 standard alcohol units, range: 0–3.99, placebo: median = 0.54 standard alcohol units, range: 0–5.96; Cohen’s *d* = 0.11) at 4-week or 6-month follow-up (abstinence: Cohen’s *d* = 0.10, alcohol use: Cohen’s *d* = 0.075). Participants in both groups reported reduced craving and temptation to drink alcohol after the dosing visit, with an additional reduction observed in the psilocybin group. Thirteen adverse events occurred in the psilocybin and seven in the placebo group. One serious adverse event occurred in the psilocybin and four in the placebo group, all related to inpatient withdrawal treatments.

**Interpretation:**

A single dose of psilocybin combined with five psychotherapy sessions may not be sufficient to reduce relapse rates and alcohol use in severely affected AUD patients following withdrawal treatment. However, given the limited sample size of our study, larger trials are needed in the future to confirm these findings.

**Funding:**

10.13039/501100001711Swiss National Science Foundation under the framework of Neuron Cofund, Swiss Neuromatrix Foundation, and Heffter Young Investigator Fellowship Award.

**Research in context**

**Evidence before this study**

Prior to conducting this study, promising evidence from the 1950s and 1960s involving clinical trials using LSD to treat alcohol use disorder (AUD) was available. More recently, two open-label pilot studies were published: one testing psilocybin for the treatment of AUD (N = 10) and another study in tobacco use disorder (N = 15), both showing encouraging results. Additionally, studies of psilocybin in the treatment of depression further provided early clinical evidence of safety and efficacy. To refine our understanding, we conducted searches using PubMed and Google Scholar with keywords including “relapse”, “withdrawal”, “pharmacological”, “treatment”, “psychedelics”, “psilocybin”, “addiction”, “alcohol use disorder”, “substance use disorder”, and “LSD”. These searches underscored the lack of effective pharmacological treatments for AUD, as relapse rates following withdrawal remain high. We identified a gap in randomized controlled trials (RCTs) investigating psilocybin’s potential to extend abstinence after withdrawal treatment.

**Added value of this study**

This RCT assesses the efficacy of a single dose of psilocybin combined with psychotherapy, in the context of relapse prevention in AUD following withdrawal treatment. Our study builds on previous open-label trials testing psilocybin for reducing alcohol use. However no previous trial has investigated psilocybin for relapse prevention in AUD. The findings presented here provide critical insights into the limitations of a single-dose approach for relapse prevention, highlighting the need for further investigation into optimal dosing regimens and accompanying psychotherapeutic approaches.

**Implications of all the available evidence**

Our results indicate that a single dose of psilocybin combined with 5.5 h of psychotherapy may not suffice to significantly reduce alcohol use or prolong abstinence in AUD patients post withdrawal treatment. This study contributes essential knowledge guiding the future development of psilocybin-assisted therapy and helping to inform optimal treatment strategies in the field of psychedelic-assisted interventions.

## Introduction

The harmful use of alcohol is a leading health risk globally.^1^ Alcohol use disorder (AUD) is often characterized by recurring cycles of treatment, periods of abstinence, and relapse.^2^^,^^3^ Increased numbers of relapse cycles are associated with more pronounced withdrawal symptoms and increased psychiatric symptoms.^4^ Relapse is typically defined as the re-initiation of alcohol use following a period of abstinence.^5^ In this study it is quantified as more than 1 standard alcohol unit (SU) per day, which was chosen to distinguish it both from minor lapses and heavy drinking days. Medications such as naltrexone and acamprosate are available to treat AUD^6^; however, relapse rates remain high — at least 60% relapse within 6-months post-treatment.^7^^,^^8^ Therefore, there is a critical unmet need for more efficacious treatments for relapse prevention after withdrawal.

Psychedelics such as LSD and psilocybin are psychoactive compounds known to induce alterations in perception, consciousness, cognition, and mood.^9^^,^^10^ Early studies in the 1950–1960s explored the clinical use of psychedelics for treating AUD, revealing promising efficacy and safety profiles.^11^ A recent proof-of-concept study and a randomized placebo-controlled trial (RCT) tested the effects of two doses of psilocybin combined with 12–14 sessions of Cognitive Behavioral Therapy and Motivational Enhancement Therapy in patients with AUD who were aiming to reduce their alcohol use rather than abstain.^12^^,^^13^ This treatment resulted in fewer heavy drinking days for up to 6 months. Considering the high relapse rates in AUD, identifying effective treatment to prolong abstinence following withdrawal is crucial. Psilocybin has shown promise in reducing alcohol use in previous studies, which suggests that it may help reduce craving and other factors that contribute to relapse. This potential to sustain reduced alcohol use makes psilocybin a promising candidate for further investigation in a relapse prevention framework in AUD.

While previous studies provide encouraging efficacy and safety data for psilocybin-assisted therapy regarding drinking reduction in active alcohol users, no RCT conducted at current standards has yet investigated whether psilocybin can prevent relapse in AUD patients who have undergone withdrawal treatment.^14^ Recent studies in depressed patients report comparable efficacy following the administration of one or two doses.^15^, ^16^, ^17^, ^18^, ^19^ However, while important for increasing accessibility, the efficacy of a single dose of psilocybin has not been tested in AUD. Therefore, in this RCT we test the efficacy of a single oral dose of psilocybin in combination with a brief psychotherapeutic intervention for preventing relapse in participants with AUD who have undergone withdrawal treatment. We hypothesize participants receiving psilocybin will show longer abstinence duration and reduced mean alcohol use between the dosing visit and 4-week follow-up compared to placebo.

## Methods

### Study design

The study was conducted at the Psychiatric University Hospital Zurich, Switzerland, as a randomized, double-blind, placebo-controlled, parallel-group design. Data assessment followed the guidelines of the revised declaration of Helsinki (2000) and Good Clinical Practice. Psilocybin was provided by the Usona Institute, Wisconsin. The trial was registered at Kofam (SNCTP000003445) and ClinicalTrials.gov (NCT04141501) and the protocol is available in the supplement.

### Ethics statement

The trial has been approved by the Cantonal Ethics Committee, Zurich, Switzerland (2019-01390), the Swiss Agency for Therapeutic Products (Swissmedic) and the Federal Office of Public Health (BAG). All participants provided written informed consent prior to study participation.

### Participants

**Fig. 1:**
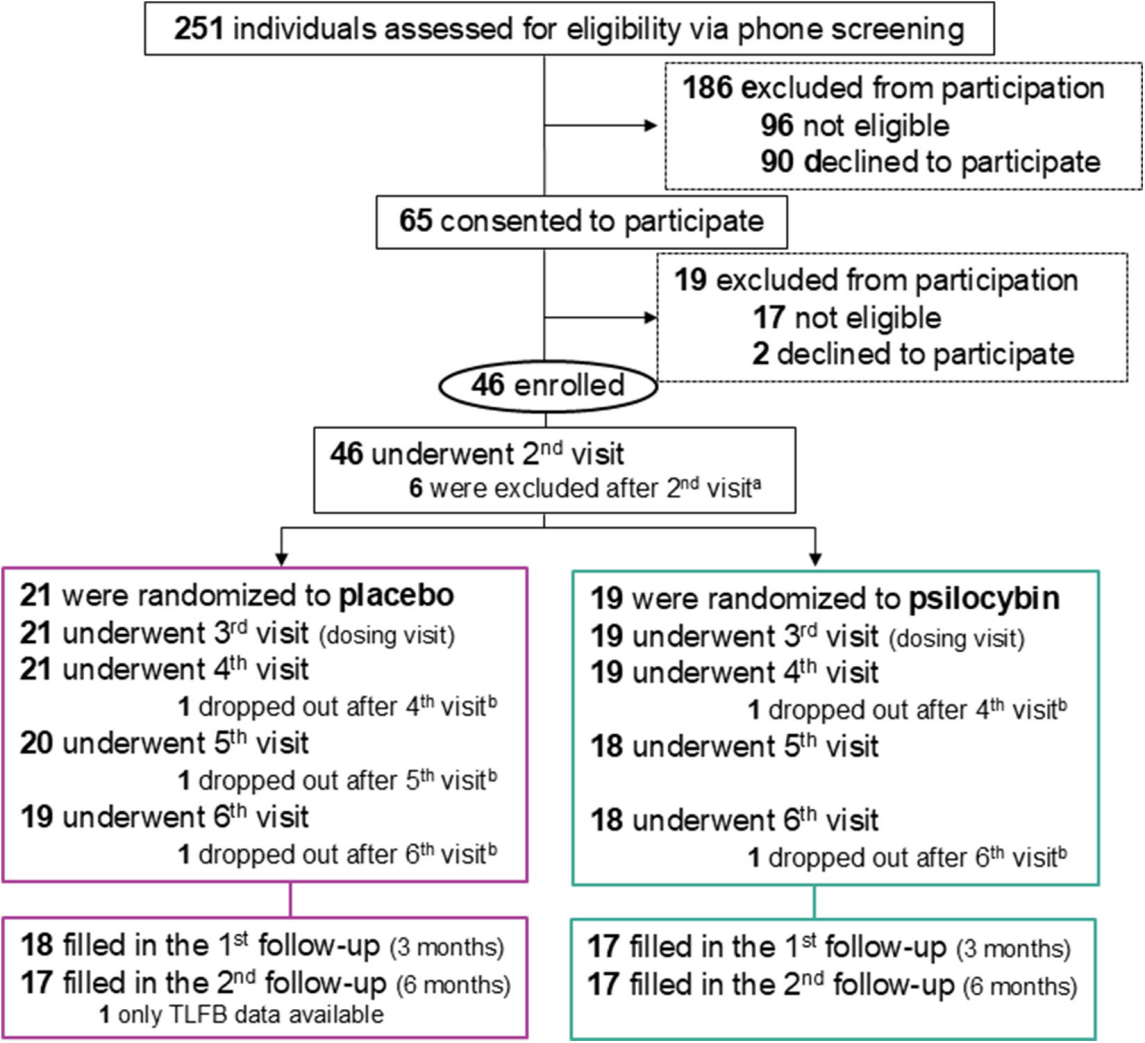
CONSORT flow diagram showing the numbers of participants at each stage of the study. a) 1: Decrease in physical well-being, unrelated to study participation; 1: TSH-value out of range, also after repetition of measurement at 2nd visit; 1: suicidal ideation; 1: abnormal brain scan; 2: Dropout due to participants’ time constraints. b) 1: Lost to follow-up. TLFB: timeline followback questionnaire assessing daily alcohol use.

German-speaking male and female participants aged 18–60 years, fulfilling the diagnosis of AUD (based on DSM-5 and the Mini International Neuropsychiatric Interview) were included. All participants completed an inpatient, outpatient, or autonomous withdrawal treatment within six weeks prior to study enrollment. Between June 8, 2020 and August 16, 2023 37 participants completed the study procedure at least up to the primary endpoint (4-week post dosing visit, see Fig. 1 and Table 1). Exclusion criteria included major psychiatric, and substance use disorders (other than alcohol and tobacco), hallucinogen use of more than ten instances within the last ten years, history of suicidal behavior, unstable physical health determined by medical history and laboratory tests, and family history of schizophrenia, schizoaffective disorder, or bipolar disorder. Full criteria are reported in the study protocol in the supplement. 18 participants received psilocybin, 19 were allocated to the placebo group. One participant in each group was lost after the 4-week follow-up, resulting in 35 participants completing the 6-month follow-up survey (Fig. 1).

**Table 1:** Participants characteristics.

	Total	Psilocybin	Placebo
Sample size	37	18	19
Sex (f:m)	14:23	7:11	7:12
Age (years), mean (SD)	37.22 (11.86)	34.8 (12.60)	39.5 (11.00)
Verbal IQ (MWT-B), mean (SD)	111.03 (13.06)	110.78 (15.63)	111.26 (10.50)
Bodyweight (kg), mean (SD)	72.23 (14.32)	73.59 (13.91)	70.94 (14.98)
BMI, mean (SD)	24.14 (3.57)	24.18 (3.56)	24.09 (3.68)
Years of education, mean (SD)	14.77 (2.68)	14.69 (2.97)	14.84 (2.46)
**Race and ethnicity**^a^			
Caucasian	37	18	19
African	1	1	0
**Alcohol use characteristics**^b^			
Drinks per day before withdrawal (units), mean (SD)	7.0 (6.45)	7.7 (7.91)	6.33 (4.82)
AUD severity (mild:moderate:severe, DSM5)	3:6:28	1:4:13	2:2:15
Days since last alcohol use, mean (SD)	15.63 (20.23)	20.05 (21.75)	11.45 (18.27)
Lifetime alcohol use (units), mean (SD)	26'370 (30'496)	31'023 (40'033)	21'962 (17'442)
**Previous withdrawal treatment (yes:no)**^c^	18:19	8:10	10:9
Number of previous outpatient withdrawal treatments, mean (SD)	1.28 (1.13) (n = 18)	1.62 (1.19) (n = 6)	1.0 (1.05) (n = 4)
Number of previous inpatient withdrawal treatments, mean (SD)	1.11 (1.08) (n = 18)	0.88 (0.83) (n = 5)	1.3 (1.25) (n = 7)
**Psychiatric comorbidities (ICD-10)**			
Depressive disorder (F31.x/F33.x)	5	3	2
Dysthmia (F34.1)	1	1	0
ADHD (F90.0)	4	1	3
Panic Disorder (F41.0)	1	0	1
Emotionally unstable personality disorder: Borderline (F60.31)	2	1	1
Use of cannabinoids: Harmful use (F12.1)	1	1	0
**Previous use of psychedelics**			
Use of psychedelics in the last 3 months (yes:no)^d^	4:33	4:14	0:19
Previous experience with psychedelics (yes:no)^d^	19:18	10:8	9:10
*If yes:* Instances of previous experiences with psychedelics, mean (SD)^e^	26.53 (51.19) (n = 19)	28.95 (64.40) (n = 10)	23.83 (34.84) (n = 9)
**Drug anamnesis**			
Smoker (yes:no)	21:16	9:9	12:7
*If yes:* Smoking (cigarettes per day), mean (SD)	5.48 (7.54) (n = 21)	5.28 (7.64) (n = 9)	5.66 (7.65) (n = 12)
Cannabis use in the last 3 months (yes:no)	11:26	5:13	6:13
Lifetime use of cannabis (yes:no)	21:16	10:8	11:8
*If yes:* Cannabis (grams per week)	3.42 (8.40) (n = 11)	5.84 (12.39) (n = 5)^f^	1.41 (2.75) (n = 6)
Cannabis (lifetime use in g)	1131.00 (2244.00) (n = 29)	1144.29 (2120.73) (n = 13)	1120.01 (2408.00) (n = 16)
MDMA use in last 3 months (yes:no)	5:32	3:15	2:17
Lifetime use of MDMA (yes:no)	19:18	10:8	9:10
*If yes:* Previous use of MDMA (instances), mean (SD)	230.00 (432.00) (n = 19)	177.01 (282.88) (n = 10)	289.60 (567.00) (n = 9)
*If yes:* Duration of this use pattern in years, mean (SD)	6.95 (5.88) (n = 19)	7.8 (7.55) (n = 10)	6.00 (3.43) (n = 9)
Cocaine use in last 3 months (yes:no)	11:26	4:14	7:12
Lifetime use of cocaine (yes:no)	21:16	10:8	11:8
*If yes:* Cocaine instances/month, mean (SD)	4.95 (8.25) (n = 21)	4.17 (9.35) (n = 10)	8.02 (11.25) (n = 11)
*If yes:* Duration of this use pattern in years, mean (SD)	5.14 (5.03) (n = 21)	4.7 (4.79) (n = 10)	5.55 (5.45) (n = 11)
Amphetamine use in the last 3 months (yes:no)	1:36	0:18	1:18
Lifetime use of amphetamine (yes:no)^g^	14:23	7:11	7:12
*If yes:* Amphetamine instances/month, mean (SD)	13.10 (27.10) (n = 14)	13.97 (35.71) (n = 7)	12.17 (17.60) (n = 7)
*If yes:* Duration of this use pattern in years, mean (SD)	7.43 (7.22) (n = 14)	9.43 (8.00) (n = 7)	5.43 (6.29) (n = 7)
Ketamine use in the last 3 months (yes:no)	1:36	0:18	1:18
Lifetime use of ketamine (yes:no)	9:28	4:14	5:14
*If yes:* Ketamine instances/month, mean (SD)	1.91 (4.92) (n = 9)	4.04 (7.32) (n = 4)	0.21 (0.26) (n = 5)
*If yes:* Duration of this use pattern in years, mean (SD)	4.78 (5.58) (n = 9)	5.00 (4.24) (n = 4)	4.60 (6.99) (n = 5)
Opiate use in the last 3 months (yes:no)	0:37	0:18	0:19
Lifetime use of opiates (yes:no)	6:31	4:14	2:17
*If yes:* Opiates lifetime instances, mean (SD)	2.50 (1.76) (n = 6)	2.75 (2.06) (n = 4)	2.00 (1.41) (n = 2)
GHB/GBL use in the last 3 months (yes:no)	0:37	0:18	0:19
Lifetime use of GHB/GBL (yes:no)	5:32	1:17	4:15
*If yes:* GHB/GBL lifetime instances, mean (SD)	47.30 (52.8) (n = 5)	104.28 (n = 1)	33.07 (48.60) (n = 4)

*Notes:* Participants who completed the study until the primary endpoint (4-week post-dosing visit).

a The total exceeds 100% because one participant selected multiple categories.

b One standard alcohol unit is 1.7 cl/10–12 g.

c In- and outpatient, unsupervised withdrawal treatment at home was not included.

d 2 participants reported ≤5 microdosing events in the last 3 months. These instances were not factored into the calculations. Two participants reported a single use of psychedelics, 1 participant two instances of psychedelic use in the last 3 months. (1: psilocybin, 1: LSD, 1: DMT, 1: Psilocybin and 5-MeO-DMT).

e Five participants reported lifetime use of microdosing. These instances were not factored into the calculations.

f One participant used 4 g cannabis/day until 2 months before study inclusion, which is included in the analysis.

g In each group one participant received medically prescribed Amphetamine as a treatment for ADHD which is included in the calculation. MWT-B: Mehrfachwahl-Wortschatz-Intelligenztest, German questionnaire assessing verbal IQ. ADHD: Attention-deficit/hyperactivity disorder. GHB: gamma hydroxybutyrate/gamma butyrolactone. LSD: Lysergic acid diethylamide. DMT: Dimethyltryptamine. 5-MeO-DMT: 5-methoxy-N,N-dimethyltryptamine.

### Study procedure

Participants completed six on-site visits over a period of six weeks: two preparation visits (approx. 2-week and 5-day prior to dosing visit), one drug administration (25 mg psilocybin or a placebo), and three integration visits (1-day, approx. 2-week, and 4-week post dosing visit), followed by two online surveys (3- and 6-month post dosing visit). Psilocybin/placebo were administered during an 8 h session in an outpatient setting under close medical and psychological supervision by two team members (one of them a trained psychiatrist). 1.5 h after drug administration, participants underwent an MRI assessment for 30 min (results will be reported in a separate publication). The drug administration was integrated in semi-structured psychotherapeutic sessions based on the BRENDA-approach,^20^ incorporating principles of motivational interviewing and cognitive-behavioral therapy and expanded to psychedelic-assisted therapy. A more detailed study procedure and underlying psychotherapy is reported in the Supplementary methods and Supplementary Fig. S1A.

### Randomization and blinding

Participants were randomized by the local hospital pharmacy with a 1:1 ratio to receive either a single dose of 25 mg psilocybin or a placebo (100% mannitol), both administered in identical capsules. The allocation process involved stratified randomization, where participants were assigned to one of 12 groups based on age (≤45 years or >45 years), sex, and the severity of AUD (mild, moderate, or severe) according to DSM-5 criteria. This stratification was implemented to achieve balanced representation of these factors across the groups. Since baseline comparisons revealed no significant differences between groups for these factors, they were not included in subsequent analyses. Each group was then randomly assigned to receive either psilocybin or placebo. This approach ensured that the distribution of participants across the various stratification factors was balanced, and that the randomization process accounted for potential confounding variables. To assess allocation blinding, the treatment providers/investigators and the patients were asked to indicate their guess of treatment allocation at 1-day follow-up with the choice of either placebo or psilocybin.

### Choice of primary measure

The primary endpoint of the study, abstinence and mean SU at 4-week follow-up, was measured using the timeline followback method.^21^ This calendar-based questionnaire is a reliable, standardized, and validated instrument to assess drinking behavior and is used routinely in clinical trials. Based on this instrument, abstinent days were assessed and relapse was defined as drinking >1 SU/day.

### Assessment of outcomes

Self-reported alcohol use prior to the dosing visit and at the 4-week follow-up was validated using urine samples, specifically measuring Ethylglucuronide. These findings confirmed abstinence and supported the accuracy of the self-reported data. Further outcome measures of alcohol use were blood markers (aspartate aminotransferase (ASAT/GOT), alanine aminotransferase (ALAT/GPT), and gamma-glutamyltransferase (Gamma-GT)), Penn Alcohol Craving Scale (PACS), and Alcohol Self-Efficacy Scale (AASE). Details on these measures are provided in the Supplementary methods.

#### Secondary endpoints

Questionnaires measuring depressive symptoms (BDI), hopelessness (HS), emotion regulation questionnaire (ERQ), positive and negative affect scale (PANAS), quality of life, anhedonia (SHAPS, TEPS), psychopathological symptom clusters (SCL-90-R), and subjective effects (5D-ASC) are described in the Supplementary methods.

### Safety assessment

Vital signs including heart rate, systolic and diastolic blood pressure were recorded at every visit, with hourly measurements during the dosing visit and increased frequency if necessary (see Supplementary Fig. S2). (Serious) adverse events ((S)AEs) were documented at each visit using an (S)AE case report form.

### Sample size and power

The study was initially designed to enroll 60 participants, with sample size determined by power analysis using G-Power 3.1 based on the effect sizes reported by Bogenschutz et al.,^12^ alpha error probability = 0.05 (two-tailed), and power = 0.95 resulting in a minimum sample size requirement of n = 24 per arm. However, due to delays associated with the Covid-19 pandemic, the study was terminated before reaching the intended enrollment goal of 60 participants. This may have contributed to an imprecision of the effect estimates, potentially affecting the study’s ability to detect differences between groups.

### Statistics

For the assessment of primary outcome; abstinence and alcohol use characteristics, the analysis was divided into 4-week (N = 37) and 6-month follow-up (n = 35). For secondary clinical measures, available data for up to the 6-month follow-up was used. Statistical analysis was conducted using R. Data were visually assessed and tested for normality using Shapiro-tests, qq-plots, and histograms and homogeneity of variances at each timepoint and across groups. Relapse (>1 SU/day) in each group was evaluated using a Kaplan–Meier survival analysis. The hazard rate of the psilocybin compared to the placebo group was obtained by a Cox Proportional Hazard Regression. Participants who relapsed immediately after the dosing visit were assigned 0 abstinent days. Group differences between psilocybin and placebo were evaluated using the Mann–Whitney U-Test for non-normally distributed data and a t-test for normally distributed data. To assess changes in blood markers of alcohol use, we conducted Wilcoxon rank sum tests with continuity correction to compare ASAT/GOT, ALAT/GPT, and Gamma-GT levels at screening vs. 4-week follow-up.

To address missing data points and account for non-normal data distribution, we utilized linear mixed models to evaluate the impact of the intervention (psilocybin vs. placebo) on clinical scores over time. More specifically, in the random coefficient models, we set the clinical scores as dependent variables (PACS, AASE, BDI, HS, ERQ, PANAS, Quality of life, SHAPS, TEPS), with a random intercept for participants considering individual differences. To account for non-linear time trends in craving (PACS), time was dummy coded (t = 1: 1-day follow-up, t = 0: other timepoints). For each secondary outcome questionnaire, we compared i) the null model; ii) a model with only time (number of the measurement: 1–8 and days between measurements: −14/-6 days to +190 days); iii) group (psilocybin vs. placebo), iv) using dummy coding for comparison between pre (t = 0) and post (t = 1) measurements; and v) a full model with time (number of the measurement: 1–8), group, and their interaction. Time, group, and their interaction were treated as fixed effects. For visualization, the mean time between measurements was used. To prevent overfitting, we used the Bayesian Information Criterion (BIC) to obtain the best parsimonious model. Overall, either the null or dummy-coded model (pre vs. post dosing visit) was best suited and subsequently reported. The normality of residuals for each model was checked visually using qq-plots. For the clinical scores all data up to the 6-month follow-up was analyzed.

Psychopathological symptoms (SCL-90-R) were assessed with differences between baseline and 4-week follow-up, using Welch’s t-tests. Information on corresponding R-packages used, protocol deviations, follow-up drug use, and therapeutic support are discussed in the Supplementary methods. The trial was monitored by an independent data monitoring committee.

### Role of funding source

The funder of the study had no role in study design, data collection, data analysis, data interpretation, or writing of the report.

## Results

Sixty-five participants consented to participate in the study between June 8, 2020, and August 16, 2023, with 46 being enrolled. Nine participants were excluded or dropped out during the study, resulting in a final sample size of 37 participants (psilocybin: 19, placebo: 18) who completed the 4-week follow-up and were included in the analysis (Fig. 1).

The primary endpoints were duration of abstinence and mean alcohol use in the 4-week period following the dosing visit. Self-report timeline followback data up to 28 days post-dosing visit from a sample of N = 37 was used. Notably, two of the participants withdrew from the study after the 4-week follow-up, providing their last reported alcohol use data at 25 and 27 days, respectively. With a final sample size of n = 35 for the 6-month follow-up analysis, we assessed mean SU per day from the dosing visit until 6-month follow-up (180 days post dosing visit, for one participant in the psilocybin group +161 days).

**Fig. 2:**
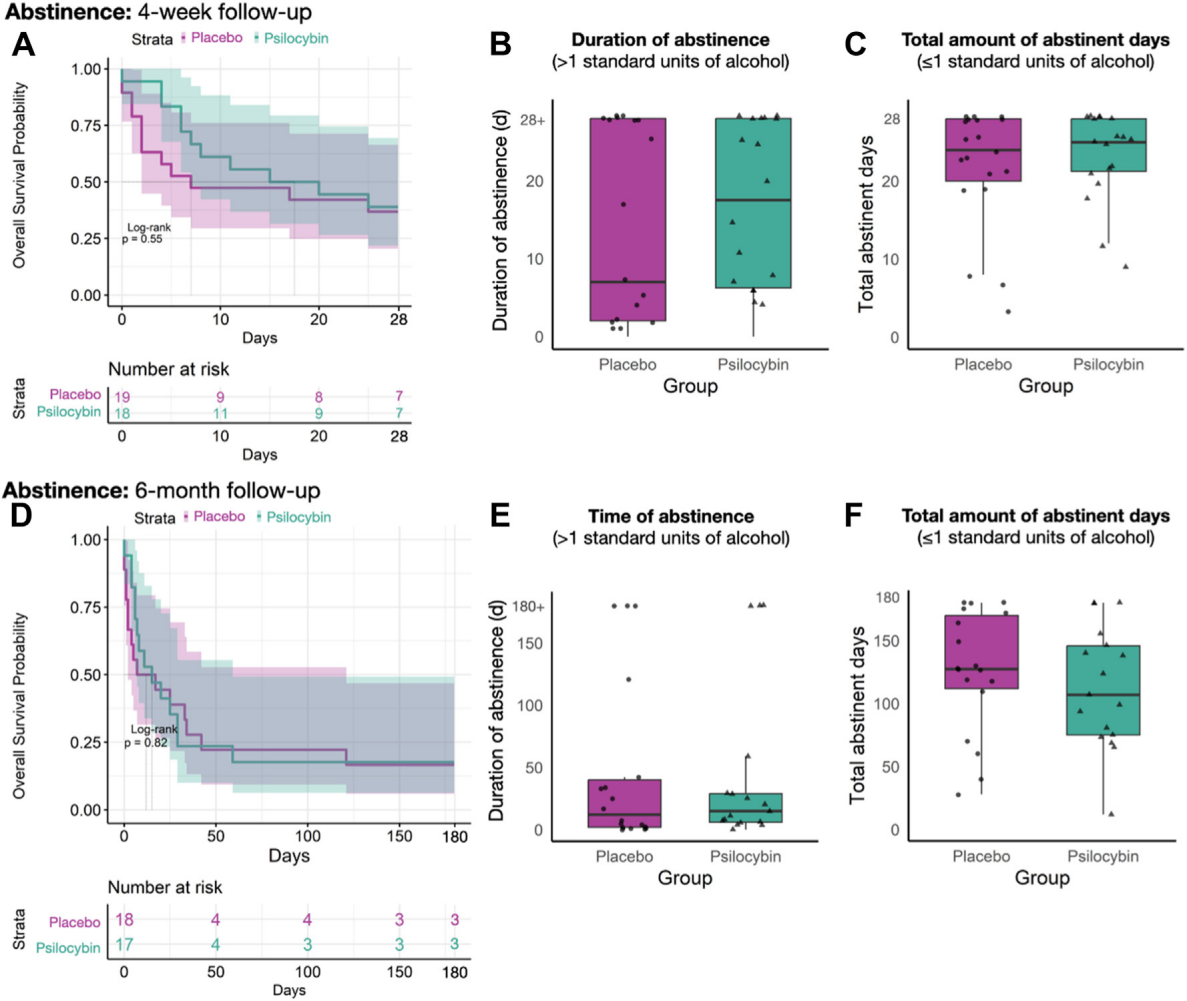
Abstinence between dosing visit and 4-week (N = 37, A–C) and 6-month follow-up (n = 35, D–F). Relapse is defined as more than 1 standard unit of alcohol in a day. A: Kaplan–Meier survival plot showing time until relapse occurred. B: Boxplot illustrating time of abstinence until the first relapse occurred. If no relapse occurred, the participant is shown on the y-axis at day 28+. One participant in the psilocybin group reported abstaining from alcohol until 25 days post-substance administration with no further data available after this timepoint and is shown on day 25. Psilocybin: mean = 16.61 days, *SD* = 10.71; placebo: mean = 13.79 days, *SD* = 12.68. C: Boxplot showing total abstinent days in the 4-week follow-up period. Psilocybin: mean = 23.28 days, *SD* = 5.61; Placebo: mean = 21.84 days, *SD* = 7.76. D: Kaplan–Meier survival plot showing time until relapse occurred. E: Boxplot illustrating time of abstinence until the first relapse occurred. If no relapse occurred, the participant is shown on the y-axis on day 180+. Psilocybin: mean = 44.88 days, *SD* = 66; placebo mean = 46.33 days, *SD* = 67.83. F: Boxplot showing total abstinent days in the 6-month follow-up period. Psilocybin: mean = 112.88 days, *SD* = 48.08; placebo: mean = 125.39 days, *SD* = 48.58. No significant group differences were observed. In each boxplot, the horizontal line inside the box indicates the median value. The lower and upper bounds of the box represent the first and third quartiles, respectively. The ‘whiskers’ extend to the minimum and maximum values within 1.5 times the IQR from the lower and upper quartiles, respectively.

Contrary to our hypothesis, the groups showed no significant difference in duration of abstinence (*p* = 0.55, psilocybin mean = 16.8 days, 95% CI: 14.31–19.29; placebo mean = 13.8 days, 95% CI: 10.97–16.63, see Fig. 2A) utilizing the Kaplan–Meier survival analysis. After testing the proportional hazards assumption, we used Cox regression to obtain an effect size estimate (reported in the Supplementary results). The hazard rate of the cox regression for the psilocybin group was 0.78 (95% CI: 0.34–1.76), indicating that the psilocybin group had a 22% lower likelihood of relapse compared to the placebo group. However, due to the limited sample size, these estimates should be interpreted with caution. Overall, 39% of the psilocybin and 37% of the placebo group stayed abstinent within 4 weeks post dosing visit. Correspondingly, duration of abstinence (*p* = 0.37, Cohen’s *d* = 0.151) and total abstinent days (*p* = 0.76, Cohen’s *d* = 0.05) did not significantly differ between the two groups (see Fig. 2B–C). Similarly, the results for the 6-month follow-up analysis revealed no group differences and are presented in the Supplementary results and Fig. 2D–F.

**Fig. 3:**
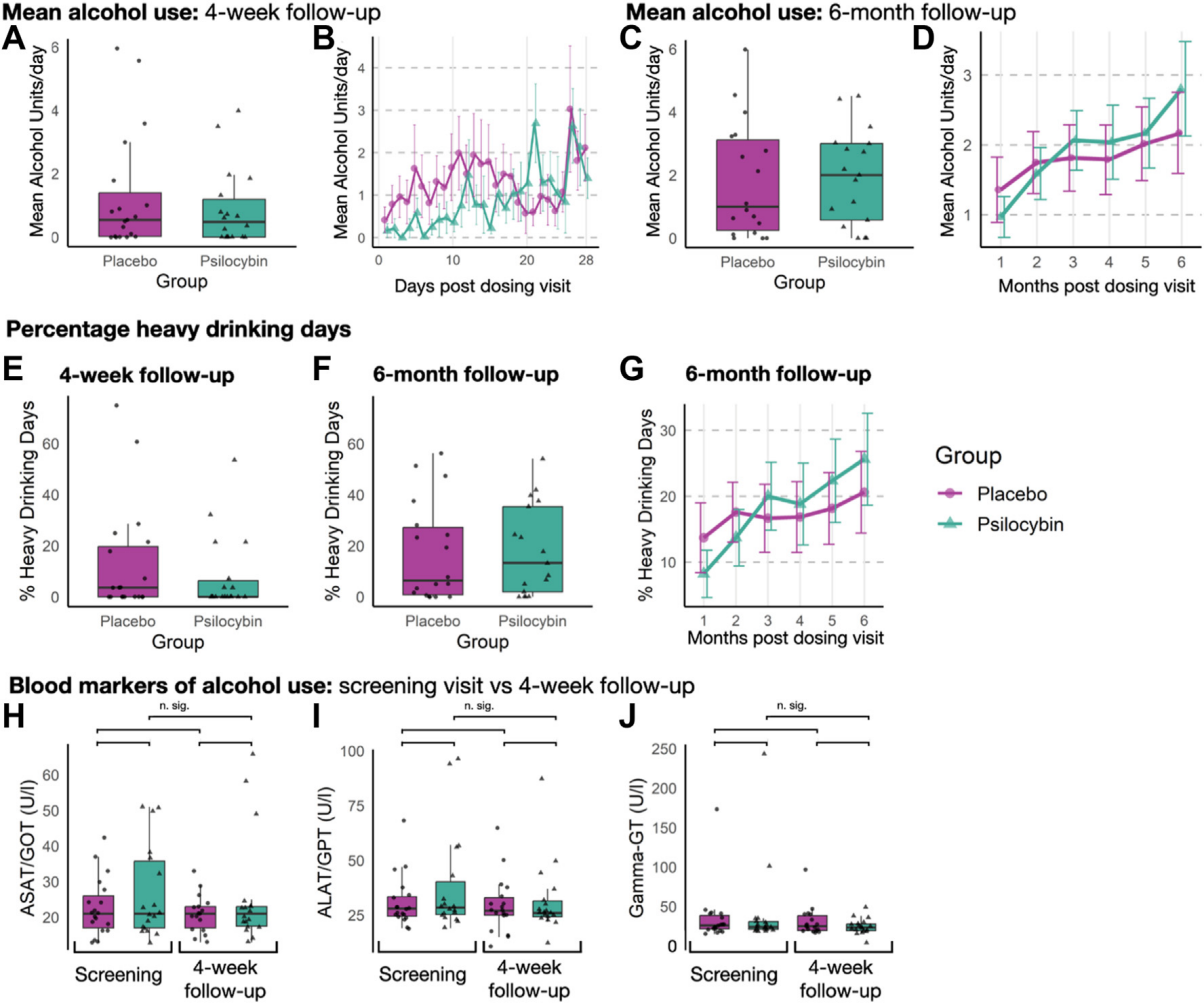
Alcohol use characteristics. Mean alcohol use between dosing visit and 4-week (N = 37, A, B, E) and 6-month follow-up (n = 35, C, D, F, G). One participant in the psilocybin group reported timeline followback data for 25 days instead of the full 28 days, while one participant in the placebo group reported data for 27 days instead of the full 28 days. One participant in the psilocybin group returned the timeline followback data for 161 days following dosing visit (instead of 180 days). A, C, E, F: Boxplot showing mean values for the corresponding time period. B, D, G: Mean ± SEM alcohol units per day over time (day of dosing visit = 0, 4-week follow-up = 28 days, 6-month follow-up = 180 days post dosing visit). No differences between the groups were observed. H–J: Boxplot showing values of blood markers of alcohol consumption aspartate aminotransferase (ASAT/GOT [U/l]), alanine aminotransferase (ALAT/GPT [U/l]), and gamma-glutamyltransferase (Gamma-GT [U/l]). As indicated, there were no significant differences observed between psilocybin and placebo neither at screening nor at 4-week follow-up and across the groups between the two timepoints. In each boxplot, the horizontal line inside the box indicates the median value. The lower and upper bounds of the box represent the first and third quartiles, respectively. The ‘whiskers’ extend to the minimum and maximum values within 1.5 times the IQR from the lower and upper quartiles, respectively.

As therapy goals such as abstinence or a reduction of alcohol use can vary over time and across participants, we also analyzed the mean alcohol use per day post-dosing visit in both groups. The psilocybin group showed no significant difference in mean SU per day compared to the placebo group between the dosing visit and the 4-week follow-up (*p* = 0.51, Cohen’s *d* = 0.111, psilocybin: median = 0.48 SU, range: 0–3.99 SU, placebo: median = 0.54 SU, range: 0–5.96 SU (Fig. 3A–B)) or the 6-month follow-up (*p* = 0.67, Cohen’s *d* = 0.075, psilocybin: median = 2.01 SU, range: 0–4.51 SU; placebo: median = 1 SU, range: 0–5.98 SU (Fig. 3C–D). We found no significant effect of sex on alcohol use characteristics at 4-week or 6-month follow-up.

We repeated the primary outcome analysis at 4-week follow-up including all randomized participants using an intention-to-treat (ITT) approach. This analysis incorporated timeline followback data for one additional participant through the 2-week follow-up, resulting in a total sample size of n = 38 (psilocybin: n = 18, placebo: n = 20). Consistent with the main survival analysis, we found no significant difference in duration of abstinence between groups (*p* = 0.445, psilocybin mean = 16.8 days; placebo mean = 13.3 days). Similarly, the analysis revealed no significant differences in duration of abstinence (*p* = 0.276, Cohen’s *d* = 0.177), total abstinent days (*p* = 0.601, Cohen’s *d* = 0.085), or mean alcohol use per day (*p* = 0.391, Cohen’s *d* = 0.139). All reported *p*-values in this section are uncorrected.

To compare our findings with prior research by Bogenschutz et al.,^13^ we also calculated percentage heavy drinking days per participant (>4 SU for females and >5 SU for males) following dosing visit and found no significant differences between groups (Fig. 3E–G and Supplementary results).

In line with self-report data, we found no difference between groups regarding blood markers of alcohol use (Fig. 3H–J and Supplementary results). Additionally, a secondary analysis revealed that standardizing mean alcohol units per day by body weight as well as body mass index did not change the results (Supplementary Fig. S3C–H).

Therefore, the current data reveal no significant impact of psilocybin-assisted therapy on abstinence or alcohol use levels after alcohol withdrawal. Notably, alcohol use before withdrawal treatment prior to study inclusion did not differ between groups (*p* = 0.85, Cohen’s *d* = 0.03, psilocybin: median = 4.38 SU, range: 1.13–29 SU, placebo: median = 5.07 SU, range: 0.57–15 SU, Supplementary Fig. S3B).

**Fig. 4:**
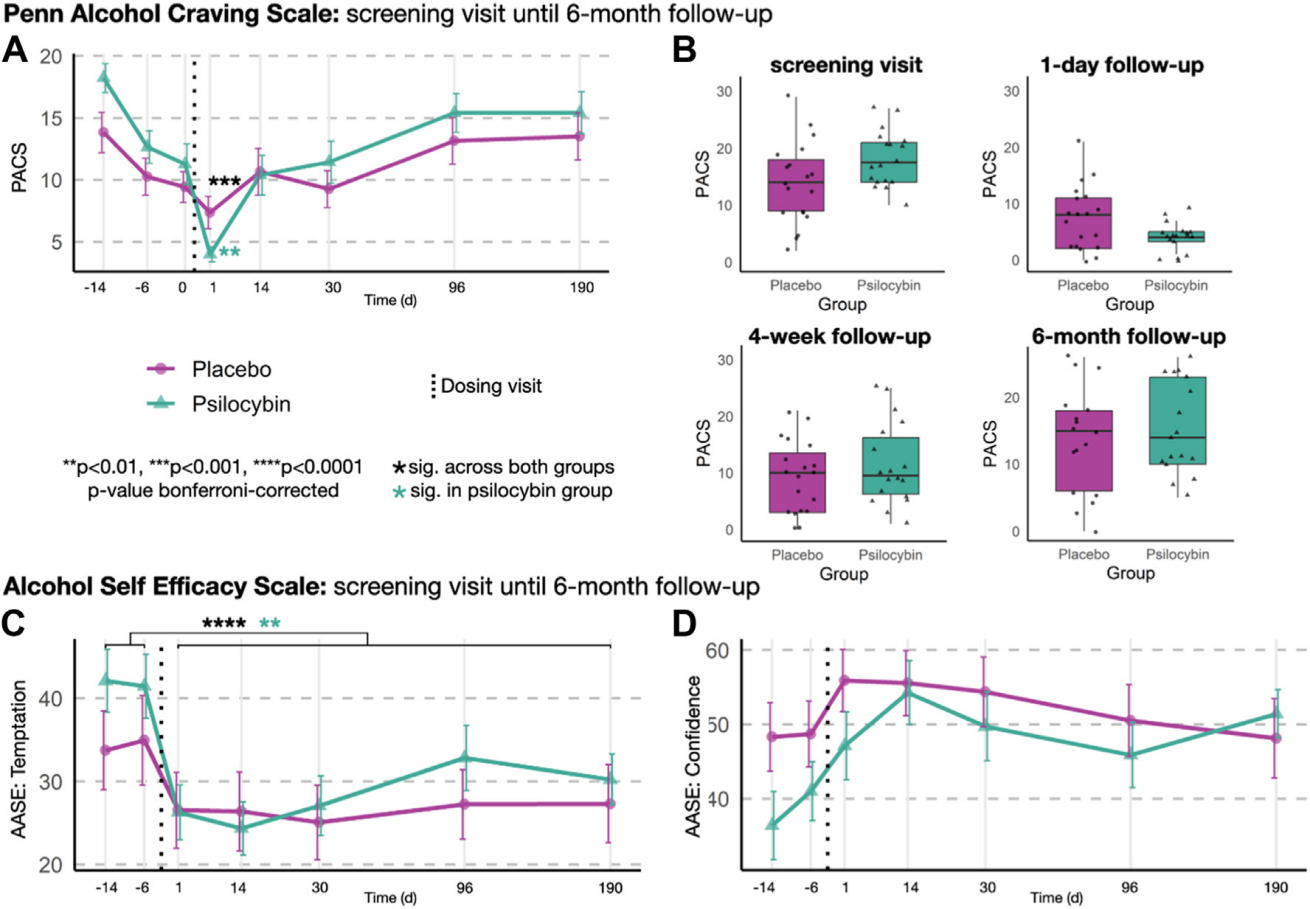
Penn alcohol craving scale and alcohol self-efficacy scale between screening and 6-month follow-up. A: Change in craving over time between screening and 6-month follow-up. Black asterisks indicate significance across both groups. Asterisks in green represent additional significant decrease in the psilocybin group. B: Boxplots depicting mean craving for individual timepoints (screening visit: approx. 14 days prior to dosing visit; 1-day follow-up: 1 day post dosing visit; 4-week follow-up: post-treatment 3, approx. 30 days post dosing visit; 6-month follow-up, approx. 190 days post dosing visit). N = 37 at timepoint −14 days, -6 days, day 0, +1 day, +30 days; n = 36 at +14 days; n = 35 at +96 days; n = 34 at +190 days. C: Change in alcohol self-efficacy subscale “temptation to drink alcohol”. Black asterisks indicate significance across both groups. Asterisks in green represent additional significant increase in the psilocybin group. D: Alcohol self-efficacy scale subscale “confidence to refrain from drinking alcohol in specific situations”. In each boxplot, the horizontal line inside the box indicates the median value. The lower and upper bounds of the box represent the first and third quartiles, respectively. The ‘whiskers’ extend to the minimum and maximum values within 1.5 times the IQR from the lower and upper quartiles, respectively. Error bars indicate the SEM. N = 37 at timepoint −14 days, −6 days, +1 day, +30 days; n = 36 at +14 days; n = 35 at +96 days; n = 34 at +190 days.

To assess the relationship between the craving scores (PACS) and time, considering the impact of group (psilocybin vs. placebo) we utilized a random coefficient model. The analysis revealed a statistically significant effect at one day follow-up (β = −4.12, CI(95%) = −6.36 to −1.87, *p* = 0.00037). This indicates a significant decrease in craving scores in both groups one day post drug administration compared to the other timepoints, with an additional significant reduction of craving in the psilocybin group (β = −5.40, CI(95%) = −8.62 to −2.19, *p* = 0.0011, Fig. 4A–B, Supplementary Fig. S4 for prediction graph, and Supplementary Table S1 for detailed model estimates).

Additionally, we conducted a random coefficient model to explore the impact of group on AASE temptation and confidence pre vs. post dosing visit. Both groups reported significantly decreased temptation to drink alcohol (β = −7.16, CI(95%) = −10.66 to −3.67, *p* < 0.0001), with an additional decrease in the psilocybin group (β = −7.71, CI(95%) = −11.71 to −1.70, *p* = 0.0089). Regarding confidence to abstain from drinking alcohol, we found no significant effect of any intervention pre to post dosing visit (Fig. 4C–D, Supplementary Fig. S4 for prediction graph, and Supplementary Table S2 for detailed model estimates). Furthermore, we repeated this analysis with an ITT approach, which revealed no substantial differences from the main results (see Supplementary results and Supplementary Tables S1 and S2).

Regarding secondary efficacy endpoints at 6-month follow-up, we observed a significant decrease in depressive symptoms below remission threshold, hopelessness, suppressive emotion regulation strategy, and negative affect following psilocybin administration in comparison to baseline and to the placebo group. More specifically, we found a decrease in depressive symptoms following the psilocybin administration (β = −4.71, 95% CI = −7.54 to −1.89, *p* = 0.0012), but not placebo (β = −0.51, 95% CI = −2.48 to 1.47, *p* = 0.61; Supplementary Fig. S5 for changes in BDI scores and prediction graph, and Supplementary Table S3 for detailed model estimates).

Participants in the psilocybin group reported significantly decreased feelings of hopelessness (β = −1.54, 95% CI = −2.91 to −0.17, *p* = 0.028) compared to baseline and the placebo group. Mean scores and prediction graph of hopelessness are shown in Supplementary Fig. S6 and detailed model estimates are reported in Supplementary Table S4.

Expressive suppression, a maladaptive emotion regulation strategy, decreased in the psilocybin group following dosing visit in comparison to the placebo group (β = −2.29, 95% CI = −4.12 to −0.47, *p* = 0.014), while an increase was observed from pre to post dosing visit in the placebo group (β = 1.43, 95% CI = 0.15–2.70, *p* = 0.028). We found no change in cognitive reappraisal. The mean scores of emotion regulation strategies and the prediction graphs for expressive suppression are shown in Supplementary Fig. S7 and model details are reported in Supplementary Table S5.

Regarding affect, compared to baseline, the negative affect significantly decreased following psilocybin (β = −2.24, 95% CI = −4.04 to −0.44, *p* = 0.015), but not placebo administration (β = −1.19, 95% = −2.45 to 0.07, *p* = 0.063). We found no changes throughout the study in positive affect. The change in positive and negative affect and prediction graph for negative affect are presented in Supplementary Fig. S8 and detailed model estimates in Supplementary Table S6.

In line with findings above, quality of life was significantly increased after the dosing visit in the psilocybin, but not the placebo group. More specifically, an increase was found in overall quality of life (β = 4.93, 95% CI = 0.74–9.13, *p* = 0.021) and marginally in physical health (β = 0.97, 95% CI = 0.09–1.85, *p* = 0.032), and environment (β = 0.71, 95% CI = 0.15–1.27, *p* = 0.013). We found no significantly changed scores in psychological health and social relationships. The change in quality of life over the study participation is presented in Supplementary Fig. S9, prediction graphs in Supplementary Fig. S10, and model estimates in Supplementary Table S7.

We found no difference in anhedonia or symptom clusters, as reported in the Supplementary results, Supplementary Figs. S11 and S12 and Tables S8–S10.

The acute subjective effects observed were comparable to those reported in previous studies involving healthy controls, despite those studies using smaller doses (115–125 μg/kg)^22^ (Supplementary Fig. S13). This finding aligns with previous reports suggesting that individuals with AUD may require higher doses than healthy controls to experience the same strength of subjective effects.^12^^,^^23^ Interestingly, we found only marginal associations between acute subjective effects (5D-ASC) and post-treatment alcohol use characteristics, which would not survive multiple comparison correction (Supplementary results and Supplementary Fig. S13).

Following drug administration, we observed a total of 13 AEs in the psilocybin and 7 in the placebo group. Additionally, 1 SAE in the psilocybin and 4 in the placebo group occurred, all of which were inpatient withdrawal treatments. This finding suggests that participants in the placebo group may have been at a heightened risk for severe withdrawal episodes, however larger studies are needed to further evaluate safety endpoints. Safety outcomes are reported in Supplementary Table S11. The psilocybin dose (25 mg) administered in our study was well-tolerated.

Participants correctly guessed their treatment assignment in 91.89% of cases. Therapists correctly guessed the treatment assignment in 97.30% of cases (Supplementary Fig. S1B).

As half of the participants reported previous psychedelic use, we exploratively examined the potential influence of previous psychedelic use on the primary outcome measures. Although underpowered, results indicated that participants in the psilocybin group with no prior psychedelic experience (n = 8) demonstrated longer durations of abstinence and lower mean alcohol use at the 4-week follow-up compared to those with prior psychedelic use (n = 10). Notably, significant differences in primary outcomes were observed between the psilocybin (n = 8) and placebo (n = 10) group when focusing solely on participants without prior psychedelic use. These findings, described in detail in the Supplementary results and Supplementary Fig. S14, suggest that prior psychedelic experience may influence treatment outcomes and should be systematically assessed in future, larger-scale studies.

## Discussion

In this RCT, we examined the efficacy of psilocybin-assisted therapy after an alcohol withdrawal to prevent relapse in individuals with AUD. 37 participants with AUD received either a single dose of psilocybin (25 mg) or placebo in combination with 5.5 h of psychotherapy.^14^ Contrary to our hypothesis, we found no significant differences between the psilocybin and placebo group in abstinence, mean alcohol use, or percentage of heavy drinking days at either the 4-week nor 6-month follow-up. However, both groups reported reduced craving immediately after the dosing visit, with the psilocybin group showing an additional decrease in craving compared to placebo. Participants in both groups felt less tempted to drink alcohol following the dosing visit in comparison to baseline, with an additional decrease in the psilocybin compared to the placebo group. Consistent with prior research, participants in the psilocybin group demonstrated reduced depressive symptoms, feelings of hopelessness, negative affect, emotion suppression, and an increase in quality of life following the dosing visit, which was not observed in the placebo group.

A previous RCT by Bogenschutz and colleagues^13^ found that two doses of psilocybin in combination with 12 psychotherapeutic sessions reduced the percentage of heavy drinking days and mean alcohol use in people with AUD. Our study did not replicate these results, however, our study design differed in several key aspects: participants were required to be abstinent at study inclusion, most underwent inpatient withdrawal treatment or required medical support during withdrawal (which was not part of our study procedure), and abstinence was a primary outcome. We administered only a single dose of psilocybin and provided five sessions of accompanying psychotherapy. Additionally, participants in our study reported higher mean alcohol use per day at baseline. Recently, preclinical studies demonstrated that the alcohol relapse severity was negatively correlated with neural responsivity to psilocybin treatment, suggesting that treatment outcome may be less effective for severe AUD cases.^24^ These differences in study design, therapeutic regimen, and patient population may account for the different outcomes of psilocybin-assisted therapy on drinking behavior.

For example, in animal studies, mice showed reduced ethanol consumption following treatment with 50 μg of LSD compared to a placebo, whereas this effect was not observed with 25 μg of LSD.^25^ In a study with rats testing the potential of psilocybin and LSD for relapse prevention after an initial phase of abstinence, neither LSD nor psilocybin had lasting effects on relapse or other markers of alcohol use, even when administered at doses translatable to those used in previous human studies.^26^ Therefore, our current results are in line with prior animal studies, suggesting that psilocybin-assisted therapy may be effective in reducing alcohol use in active drinkers, but may not prevent relapse after withdrawal.

Participants in an open-label pilot study reported increased confidence to abstain from alcohol at 5 weeks and decreased temptation at 9 weeks following psilocybin administration.^12^ Additionally, they showed decreased craving at 8–12 weeks post psilocybin administration. Given that the open label study included no placebo group, it remains unknown whether the psilocybin-assisted therapy decreased craving and increased self-efficacy to a stronger extent than placebo-assisted therapy itself. In our study, altered temptation and craving occurred immediately after the dosing visit and were visible in both groups, with an additional decrease in the psilocybin group.

Given that AUD is typically comorbid with other psychiatric symptoms,^27^ we also assessed secondary clinical outcomes. Participants reported improvements in clinical outcome measures following psilocybin administration, but not placebo. First, in line with previous findings demonstrating decreased depressive symptom severity following a single dose of psilocybin-assisted therapy in depression,^15^^,^^28^ the participants undergoing psilocybin-assisted therapy exhibited reduced depressive symptoms compared to baseline and the placebo group. Specifically, participants receiving psilocybin exhibited a decline below remission thresholds, which was not observed in the placebo group. This suggests that the antidepressant effects of psilocybin in depressive patients may extend to patients with AUD. Second, in line with previously reported increased quality of life in depression and anxiety patients following psilocybin-assisted therapy,^28^^,^^29^ we found improved quality of life post psilocybin administration in AUD patients.

While improvements in depressive symptoms and quality of life are important indicators of overall recovery, these changes did not translate into significant reductions in alcohol use outcomes. The absence of an impact on alcohol use, despite improvements in related psychopathology, highlights the potential need for targeted psychotherapeutic approaches when using psilocybin-assisted therapy in patients with AUD. This specificity of treatment effects requires further investigation and suggests that a more resource-intensive psilocybin-assisted therapy regimen may be necessary for efficacy in this population. This could involve repeated dosing sessions, longer-term follow-up psychotherapeutic sessions, or pairing psychedelic-assisted therapy with additional training such as neurofeedback or virtual reality.^30^^,^^31^ This approach is particularly relevant for a relapse prevention framework aiming for continued abstinence in moderately to severely affected patients with AUD.

Several limitations of this clinical trial should be noted. First, ∼90% of participants correctly guessed their treatment allocation, which may have compromised the integrity of the placebo-controlled, double-blind design. The primary outcome measures relied on self-report data, introducing the potential for bias. That said, objective alcohol use markers replicated the observed participant-reported outcomes. Most participants were Caucasian, reflecting the population distribution in Switzerland. To ensure the generalizability of findings, larger trials incorporating more diverse populations are warranted. Additionally, participants underwent fMRI scanning for 30 min after drug administration, which may have interfered with the therapeutic action of psilocybin. However, participants could focus on the effects of psilocybin for the remainder of the trip and reported pronounced subjective alterations in consciousness. Due to Covid-19 restrictions, we fell short of our recruitment goals, limiting the interpretation of our results. While the study may have been underpowered to detect smaller effects, the consistent lack of group differences in all alcohol use measures suggests that psilocybin-assisted therapy, as implemented here, is not effective for preventing relapse in AUD.

While a previous RCT reported promising outcomes regarding the efficacy of psilocybin-assisted therapy for reducing heavy drinking days in active alcohol users, the current results suggest that psilocybin-assisted therapy following alcohol withdrawal may not be effective in preventing relapse. Although our study did not find significant improvements in abstinence or other alcohol use parameters, we did observe a transient reduction in craving and temptation, and significant improvements in secondary clinical measures including reductions in depressive symptoms, hopelessness, negative affect, and emotion suppression, along with an overall increase in quality of life. Despite these improvements, our findings suggest that achieving sustained benefits in patients with moderately to severe AUD may require a more intensive pharmaco-psychotherapeutic approach than was implemented in this study. Future research should explore the impact of different dosing regimens, alongside more intensive or prolonged psychotherapeutic interventions, on treatment efficacy.

## Contributors

All authors have full access to the data in the study, take responsibility for the integrity of the data, contributed to and have approved the final manuscript.

*Concept and design:* Preller, Herdener, Vollenweider, Rieser.

*Acquisition, analysis, or interpretation of data:* Rieser, Bitar, Halm, Rossgoderer, Ort, Gubser, Thévenaz, Kreis, Nordt, Moujaes, Engeli, von Rotz, Preller, Herdener.

*Drafting of the manuscript:* Rieser, Preller, Herdener.

*Critical review of the manuscript for intellectual content:* All authors.

*Statistical analysis:* Rieser, Preller.

*Verified underlying data:* Visentini, Rieser.

*Obtained funding:* Preller, Vollenweider.

*Administrative, technical, or material support:* Rieser, Preller, Herdener, Gubser.

*Supervision:* Preller, Vollenweider, Herdener.

## Data sharing statement

Data and data dictionary will be shared upon reasonable request. Data include individual de-identified participant data and de-identified individual responses to self-report assessments, interviews, and physiological data. Related documents (study protocol and informed consent) will be made available upon publication.

## Declaration of interests

Katrin H. Preller is currently an employee of Boehringer Ingelheim GmBH & CO KG, Chief Scientist and on the Board of Directors of the Heffter Research Institute, and scientific advisor for the MIND Foundation.

Marcus Herdener has received consulting fees from Boehringer-Ingelheimer and serves as a board member of Swiss Society for Addiction Medicine and Swiss Society for Biological Psychiatry.

Franz X. Vollenweider is currently on the Board of Directors of the Heffter Research Institute, and scientific advisor for the USONA Institute and the MIND foundation.

Robin von Rotz is an employee of and owns stock in Reconnect Labs AG.

Erich Seifritz has received consulting fees, payments, or honoraria for presentations from Lundbeck, Janssen, OM Pharma, Schwabe Pharma, Takeda, Sandoz, Salmon, Mepha, Recordati, Zeller Pharma, Idorsia Pharma, Servier, and Otsuka. Payment for expert testimony was received from Janssen. Support for attending meetings was provided by Schwabe Pharma. Erich Seifritz has participated in data safety monitoring boards or advisory boards for Lundbeck, Janssen, OM Pharma, Schwabe Pharma, Otsuka, and Recordati. Leadership or fiduciary roles have been held in the Swiss Society of Psychiatry and Psychotherapy, Swiss Mental Health Care, Swiss Society of Anxiety and Depression, and the Swiss Conference of Academic Psychiatry. The author holds stocks in AbCellera and Idorsia.

All other authors declare no competing interests.

## Appendix ASupplementary data

Clinical Study Protocol

Supplementary materials

Informed Consent
