# Supplementary material for: Psilocybin-assisted therapy for relapse prevention in alcohol use disorder: a phase 2 randomized clinical trial
Source: eClinicalMedicine. 2025 Mar 14;82:103149. doi: 10.1016/j.eclinm.2025.103149 (PMC11937691; doi:10.1016/j.eclinm.2025.103149)
Supplement: Clinical Study Protocol [file mmc1.pdf]

Clinical Study Protocol

PHASE II, RANDOMIZED, DOUBLE BLIND, PLACEBO CONTROLLED, PARALLEL GROUP, SINGLE CENTER STUDY OF PSILOCYBIN EFFICACY AND MECHANISM IN ALCOHOL USE DISORDER

"Clinical and Mechanistic Effects of Psilocybin in Alcohol Addicted Patients"

Study Type:	Intervention with Investigational Medicinal Product (IMP)
Study Categorisation:	Clinical Trial with IMP Category C
Study Registration:	ClinicalTrials.gov (intended registry)
Study Identifier:	PSIALC-133 (institutional identifier)
Sponsor:	Prof. Dr. med. Franz X. Vollenweider<br>Department of Psychiatry, Psychotherapy and Psychosomatics<br>Neuropsychopharmacology and Brain Imaging<br>Psychiatric Hospital, University of Zurich<br>Lenggstrasse 31<br>CH-8032 Zürich,<br>Switzerland<br>E-mail: vollen@bli.uzh.ch<br>Phone: +41 58 384 24 04
Principal Investigator:	PD Dr. med. Marcus Herdener<br>Department of Psychiatry, Psychotherapy and Psychosomatics<br>Center for Addiction Disorders<br>Selnaustrasse 9<br>CH-8001 Zürich,<br>Switzerland<br>E-mail: marcus.herdener@bli.uzh.ch<br>Phone: +41 44 205 58 11
Investigational Product:	Psilocybin
Protocol Version and Date:	Version 7, 30.01.2023

CONFIDENTIAL

The information contained in this document is confidential and the property of the Psychiatric University Hospital Zürich. The information may not - in full or in part - be transmitted, reproduced, published, or disclosed to others than the applicable Independent Ethics Committee(s) and Competent Authority(ies) without prior written authorization from the Psychiatric University Hospital Zürich, except to the extent necessary to obtain informed consent from those participants who will participate in the study.

Signature Page(s)

**Study number**	ClinicalTrials.gov (intended registry)
**Study Title**	PHASE II, RANDOMIZED, DOUBLE BLIND, PLACEBO CONTROLLED, PARALLEL GROUP, SINGLE CENTER STUDY OF PSILOCYBIN EFFICACY AND MECHANISM IN ALCOHOL USE DISORDER

The Sponsor and the Principal Investigator have approved the protocol version 7 of 30.01.2023, and confirm hereby to conduct the study according to the protocol, current version of the World Medical Association Declaration of Helsinki, ICH-GCP guidelines or ISO 14155 norm if applicable and the local legally applicable requirements.

Sponsor

Prof. Dr. med. Franz X. Vollenweider

Zürich, 30.01.2023

Place/Date Signature

Principal Investigator:

PD Dr. med. Marcus Herdener

Zürich, 30.01.2023

Place/Date Signature

Local Principal Investigator at study site:

I have read and understood this trial protocol and agree to conduct the trial as set out in this study protocol, the current version of the World Medical Association Declaration of Helsinki, ICH-GCP guidelines or ISO 14155 norm and the local legally applicable requirements.

**Site**	Department of Psychiatry, Psychotherapy and Psychosomatics<br>Neuropsychopharmacology and Brain Imaging<br>Psychiatric Hospital, University of Zurich<br>Lenggstrasse 31<br>CH-8032 Zürich

**Principal investigator**	PD Dr. med. Marcus Herdener

Zürich, 30.01.2023	
Place/Date	Signature

Table of Contents

STUDY SYNOPSIS 7
ABBREVIATIONS 11
STUDY SCHEDULE 13
1. STUDY ADMINISTRATIVE STRUCTURE 16
1.1 Sponsor 16
1.2 Principal Investigator (PI) 16
1.3 Investigators 16
1.4 Statistician ("Biostatistician") 17
1.5 Laboratory 18
1.5.1 On-site laboratory 18
1.5.2 Collaborating laboratory 18
1.6 Monitoring institution 19
1.7 Data Safety Monitoring Committee 19
1.8 Any other relevant Committee, Person, Organisation, Institution 19
2. ETHICAL AND REGULATORY ASPECTS 20
2.1 Study registration 20
2.2 Categorisation of study 20
2.3 Competent Ethics Committee (CEC) 20
2.4 Competent Authorities (CA) 20
2.5 Ethical Conduct of the Study 20
2.6 Declaration of interest 21
2.7 Patient Information and Informed Consent 21
2.8 Participant privacy and confidentiality 21
2.9 Early termination of the study 21
2.10 Protocol amendments 22
3. BACKGROUND AND RATIONALE 23
3.1 Background and Rationale 23
3.2 Investigational Product and Indication 25
3.3 Preclinical Evidence 25
3.4 Clinical Evidence to Date 26
3.5 Dose Rationale 26
3.6 Explanation for choice of comparator (or placebo) 26
3.7 Risks / Benefits 27
3.7.1 Potential Risks 27
3.7.2 Mitigation of risks 29
3.7.3 Potential benefits 30
3.8 Justification of choice of study population 30
4. STUDY OBJECTIVES 31
4.1 Overall Objective 31
4.2 Primary Objective 31
4.3 Secondary Objectives 31
4.4 Safety Objectives 31
5. STUDY OUTCOMES 33
5.1 Primary Outcome 33
5.2 Secondary Outcomes 33

5.3 Other Outcomes of Interest ... 36
5.4 Safety Outcomes ... 36
6. STUDY DESIGN ... 37
6.1 General study design and justification of design ... 37
6.2 Methods of minimising bias ... 37
6.2.1 Randomisation ... 37
6.2.2 Blinding procedures ... 37
6.2.3 Other methods of minimising bias ... 38
6.3 Unblinding Procedures (Code break) ... 38
7. STUDY POPULATION ... 39
7.1 Eligibility criteria ... 39
7.2 Recruitment and screening ... 40
7.3 Pilot Study ... 40
7.4 Assignment to study groups ... 41
7.5 Criteria for withdrawal / discontinuation of participants ... 41
8. STUDY INTERVENTION ... 42
8.1 Identity of Investigational Products ... 42
8.1.1 Experimental Intervention ... 42
8.1.2 Control Intervention ... 42
8.1.3 Packaging, Labelling and Supply (re-supply) ... 42
8.1.4 Storage Conditions ... 43
8.2 Administration of experimental and control interventions ... 43
8.2.1 Experimental Intervention ... 43
8.2.2 Control Intervention ... 43
8.3 Dose / Device modifications ... 43
8.4 Compliance with study intervention ... 43
8.5 Data Collection and Follow-up for withdrawn participants ... 44
8.6 Trial specific preventive measures ... 44
8.7 Concomitant Interventions (treatments) ... 45
8.8 Study Drug / Medical Device Accountability ... 45
8.9 Return or Destruction of Study Drug ... 45
9. STUDY ASSESSMENTS ... 46
9.1 Study flow chart / table of study procedures and assessments ... 46
9.2 Assessments of outcomes ... 48
9.2.1 Assessment of primary outcome ... 48
9.2.2 Assessment of secondary outcomes ... 48
9.2.3 Assessment of other outcomes of interest ... 49
9.2.4 Assessment of safety outcomes ... 49
9.2.5 Assessments in participants who prematurely stop the study ... 50
9.3 Procedures at each visit ... 50
9.3.1 Pre-Screening (Phone Call) ... 50
9.3.2 Visit 1/Screening Visit ... 50
9.3.3 Visit 2/Pre-treatment and Baseline Visit ... 50
9.3.4 Visit 3/Treatment Visit ... 51
9.3.5 Visit 4/Post-treatment visit ... 51
9.3.6 Visit 5/Post-treatment visit ... 52

9.3.7 Visit 6/Post-treatment visit ... 52
9.3.8 Visit 7/Follow-up (Survey) ... 52
9.3.9 Visit 8/Follow-up (Survey) ... 53
10. SAFETY ... 53
10.1. Definition and assessment of (serious) adverse events and other safety related events ... 53
10.2. Reporting of serious adverse events (SAE) and other safety related events ... 54
10.3. Follow up of (Serious) Adverse Events ... 55
11. STATISTICAL METHODS ... 56
11.1 Hypothesis ... 56
11.2 Determination of Sample Size ... 56
11.3 Statistical criteria of termination of trial ... 56
11.3.1 Criteria of stopping for benefit ... **Error! Bookmark not defined.**
11.3.2 Criteria of stopping for futility ... 56
11.3.3 Criteria of stopping for harm ... 56
11.4 Planned Analyses ... 56
11.4.1 Datasets to be analysed, analysis populations ... 56
11.4.2 Primary Analysis ... 56
11.4.3 Secondary Analyses ... 56
11.4.4 Interim analyses ... 57
11.4.5 Safety analysis ... 57
11.4.6 Deviation(s) from the original statistical plan ... 57
11.5 Handling of missing data and drop-outs ... 57
12. QUALITY ASSURANCE AND CONTROL ... 58
12.1 Data handling and record keeping / archiving ... 58
12.1.1 Case Report Forms ... 58
12.1.2 Specification of source documents ... 58
12.1.3 Record keeping / archiving ... 59
12.2 Data management ... 59
12.2.1 Data Management System ... 59
12.2.2 Data security, access and back-up ... 59
12.2.3 Analysis and archiving ... 60
12.2.4 Electronic and central data validation ... 60
12.3 Monitoring ... 60
12.4 Audits and Inspections ... 60
12.5 Confidentiality, Data Protection ... 60
12.6 Storage of biological material and related health data ... 61
13. PUBLICATION AND DISSEMINATION POLICY ... 62
14. FUNDING AND SUPPORT ... 63
14.1 Funding ... 63
15. INSURANCE ... 63
16. REFERENCES ... 64

STUDY SYNOPSIS

**Sponsor**	Prof. Dr. med. Franz X. Vollenweider
**Study Title:**	Phase II, randomized, double blind, placebo controlled, parallel group, single center study of psilocybin efficacy and mechanism in alcohol use disorder
**Short Title / Study ID:**	Clinical and Mechanistic Effects of Psilocybin in Alcohol Addicted Patients / PSIALC-133
**Protocol Version and Date:**	Version 6 from 28.01.2022
**Trial registration:**	ClinicalTrials.gov (intended registry)
**Study category and Rationale**	Clinical study with IMP Category C
**Clinical Phase:**	Clinical Phase II
**Background and Rationale:**	Two billion people globally consume alcohol, leading in 2016 to 2.8 million deaths (5.2% of all deaths) and 99.2 million Disability Adjusted Life Years (DALYs) lost (4.2% of all DALYs). Of all the diseases, conditions, and injuries attributable to alcohol use, alcohol use disorders (AUDs) create the largest health burden globally. However, approved pharmacological treatments for alcoholism are limited in their effectiveness. A recent proof-of-concept study testing psilocybin in ten alcohol dependent patients provides encouraging efficacy results and safety data. We, therefore, propose to test the efficacy of psilocybin for treating alcohol use disorder and study its underlying neurobiological mechanisms in a randomized, placebo controlled, double blind study.
**Objective(s):**	To evaluate effects of psilocybin on alcohol use behaviour, clinical symptoms, neurocognitive and emotional measures in patients with alcohol use disorder.<br>A pilot study with healthy participants will be conducted to test feasibility of fMRI tasks.
**Outcome(s):**	Primary outcome measures:<br>Alcohol use behaviour measured with the Time-Line Follow-Back<br><br>Secondary outcome measures:<br>• changes in functional connectivity during resting-state, changes in cue-reactivity, and autobiographic memory assessed with fMRI,<br>• changes in empathy assessed with the Multifaceted Empathy Task,<br>• changes in personality, self-perception, and patient-therapist relationship assessed with questionnaires, and<br>• epigenetic mechanisms and in vitro neural profile<br>• additional assessments of alcohol drinking behaviour<br>Pilot study: feasibility of fMRI tasks
**Study design:**	Randomized, double blind, placebo controlled, parallel group, single center study<br>Pilot study: open-label, not randomized

**Inclusion / Exclusion criteria:**	Participants fulfilling all of the following <u>inclusion</u> criteria are eligible for the study:<br>- Informed Consent as documented by signature (Appendix Informed Consent Form)<br>- Male and female in- and outpatients 18 years to 60 years of age<br>- Right-handedness according to Oldfield (1971) performed during the telephone screening, laterality index ≥ 0.2<br>- DSM-IV-diagnosis of alcohol use disorder (based on clinical assessment and confirmed by the SCID Interview)<br>- Having undergone withdrawal treatment from alcohol use or have stopped using alcohol within 6 weeks prior to enrolment in the study<br>- Drug free from any psychotropic and serotonergic medication for at least five days before administration of the study drug or placebo<br>- No alcohol use between withdrawal treatment and administration of study drug or placebo<br>- Good physical health with no unstable medical conditions, as determined by medical history, physical examination, routine blood labs, electrocardiogram, urine analysis, and urine toxicology<br>- Normal level of language comprehension (German or Swiss-German)<br>- Willing to refrain from drinking caffeinated drinks during the testing days and from consuming psychoactive substances after enrolling in the study until visit 6<br>- Women of childbearing potential must be using an effective, established method of contraception for the entire study duration, such as oral, injectable, or implantable contraceptives, or intrauterine contraceptive devices. Note: female participants who are surgically sterilised / hysterectomised or post-menopausal for longer than 2 years are not considered as being of child bearing potential.<br>- Have a family member or friend who can pick them up and stay with them overnight after the psilocybin administration sessions (driving is forbidden at drug treatment days)<br>- No other medication than reported at study inclusion is allowed until visit 6, except for emergencies<br><br>The presence of any one of the following <u>exclusion</u> criteria will lead to exclusion of the participant, for example:<br>- Allergy, hypersensitivity, or other adverse reaction to previous use of psilocybin or other hallucinogens<br>- Uncorrected Hypertension (assessed at screening day: higher than 139 systolic and 89 diastolic)<br>- Women who are pregnant or breast feeding<br>- Intention to become pregnant during the course of the study,<br>- Lack of safe contraception, defined as: Female participants of childbearing potential, not using and not willing to continue using a medically reliable method of contraception for the entire study duration, such as oral, injectable, or implantable contraceptives, or intrauterine contraceptive devices, or who are not using any other method considered sufficiently reliable by the investigator in individual cases (Female participants who are surgically sterilised / hysterectomised or post-menopausal for longer than 2 years are not considered as being of child bearing potential)<br>- Known or suspected non-compliance<br>- Inability to follow the procedures of the study, e.g. due to language problems, psychological disorders, dementia, etc. of the participant,<br>- Previous enrolment into the current study

	- Enrolment of the investigator, his/her family members, employees and other dependent persons<br>- Lifetime history of bipolar disorder (I, II, not otherwise specified) based on our clinical interview at screening visit<br>- Lifetime history of schizophrenia, schizoaffective disorder, or psychosis not otherwise specified based on our clinical interview at screening visit, expect psychotic symptoms under acute alcohol intoxication or during withdrawal treatment<br>- History of DSM-IV drug dependence other than alcohol (except for caffeine or nicotine) within two months prior to enrolment<br>- Comorbid Axis I anxiety and depression disorders diagnoses as well as post-traumatic stress disorder and ADHD will be permitted if they do not require treatment after inclusion in the study<br>- Family history of schizophrenia or schizoaffective disorder, or bipolar disorder type 1 (first or second degree relatives)<br>- Violent behaviour within last 2 years or history of suicidal behaviour<br>- Lifetime history of hallucinogen use on more than 10 occasions within last 10 years<br>- Getting psychotherapeutic or psychological treatment from third parties during the study until visit 6 is forbidden<br>- Abnormal electrocardiogram<br>- Any unstable illness as determined by history or laboratory tests<br>- BMI <17 or >30<br>- Uncorrected hypo- or hyperthyroidism<br>- Contraindications to magnetic resonance imaging (MRI safety form)<br>- During the study, new use or dose changes of already existing concomitant medication without prior informing the investigators is forbidden<br>- High risk of adverse emotional or behavioural reaction based on investigator’s clinical evaluation (e.g., evidence of serious personality disorder, antisocial behaviour, serious current stressors, lack of meaningful social support)<br>- Participation in another study with investigational drug within the 30 days preceding and during the present study (until visit 6)<br>- Taking medications that are known to modulate uridine diphosphate glucuronosyltransferase enzyme<br>- Inhibitors of UGT1A9 and 1A10 should be discontinued at least five half-lives prior to the administration of psilocybin<br>- Monoamine oxidase and aldehyde or alcohol dehydrogenase inhibitors should be discontinued at least 5 half-lives prior to the dose of psilocybin

**Measurements and procedures:**	Patients with alcohol use disorder who have undergone withdrawal treatment within the last 6 weeks will be investigated in a single-centre, double-blind, placebo-controlled, parallel-group design clinical trial contrasting the acute and persisting effects of psilocybin to those of placebo. Patients will be randomly assigned to psilocybin or placebo group with a 1:1 allocation ratio.<br>The study comprises a total of 6 visits during 6 weeks and two follow-up online surveys (3 and 6 months after treatment). The following visits will be conducted:<br>• Screening visit<br>• Pre-treatment visit<br>• Treatment visit<br>• Post-treatment visit 1<br>• Post-treatment visit 2<br>• Post-treatment visit 3<br>In addition, two follow-up surveys that can be completed from home will guarantee monitoring of long-lasting changes in symptomology and ensure all potential side-effects can be captured.<br>On the treatment visit, a single dose of psilocybin (25mg) or placebo will be administered. Patients will be monitored until all effects have worn off.
**Study Product / Intervention:**	Psilocybin (25 mg orally); treatment arm 1; one-time application of a single dose
**Control Intervention (if applicable):**	Placebo (100% mannitol orally); treatment arm 2; one-time application of a single dose
**Number of Participants with Rationale:**	30 patients in arm 1 and 30 patients in arm 2, total N=60 (determined by power analysis)<br>Pilot study: a maximum of 15 participants
**Study Duration:**	3 years
**Study Schedule:**	First-patient-in: 01/2020 (planned)<br>Last-patient-out: 12/2023 (planned)
**Investigator(s):**	PD Dr. med. Marcus Herdener<br>Department of Psychiatry, Psychotherapy and Psychosomatics<br>Centre for Addictive Disorders,<br>Selnaustrasse 9<br>CH-8001 Zürich<br>E-Mail: marcus.herdener@bli.uzh.ch<br>Phone: +41 44 205 58 00<br><br>Dr. med. Raoul Bitar<br>Department of Psychiatry, Psychotherapy and Psychosomatics<br>Centre for Addictive Disorders, Special Ward E1 for Dual Diagnoses<br>Psychiatric Hospital, University of Zurich<br>Lenggstrasse 31<br>CH-8032 Zürich,<br>E-mail: raoul.bitar@puk.zh.ch<br>Phone: +41 58 384 33 77<br><br>Dr. med. Christina Rossgoderer Pinto da Silva<br>Psychiatric Hospital, University of Zurich<br>Lenggstrasse 31<br>CH-8032 Zürich,<br>Switzerland<br>E-mail: christina.rossgoderer@pukzh.ch<br>Phone: +41 58 384 23 29

	M.Sc. Nathalie Rieser<br>Department of Psychiatry, Psychotherapy and Psychosomatics<br>Neuropsychopharmacology and Brain Imaging<br>Psychiatric Hospital, University of Zurich<br>Lenggstrasse 31<br>CH-8032 Zürich,<br>E-mail: nathalie.rieser@bli.uzh.ch<br>Phone: +41 58 384 33 24<br><br>Pract. Med. Simon Halm<br>Department of Psychiatry, Psychotherapy and Psychosomatics<br>Centre for Addictive Disorders,<br>Selnaustrasse 9<br>CH-8001 Zürich<br>E-Mail: simon.halm@pukzh.ch<br>Phone: +41 44 205 58 60<br><br>M.Sc. Robin von Rotz<br>Department of Psychiatry, Psychotherapy and Psychosomatics<br>Neuropsychopharmacology and Brain Imaging<br>Psychiatric Hospital, University of Zurich<br>Lenggstrasse 31<br>CH-8032 Zürich,<br>E-mail: robin.vonrotz@bli.uzh.ch<br>Phone: +41 58 384 2616<br><br>Dr. med. Andres Ort<br>Department of Psychiatry, Psychotherapy and Psychosomatics<br>Neuropsychopharmacology and Brain Imaging<br>Psychiatric Hospital, University of Zurich<br>Lenggstrasse 31<br>CH-8032 Zürich,<br>E-mail: andres.ort@bli.uzh.ch<br>Phone: +41 58 384 26 03
**Study Centre(s):**	Single centre: Department of Psychiatry, Psychotherapy and Psychosomatics, Neuropsychopharmacology and Brain Imaging, Psychiatric Hospital, University of Zurich, Switzerland
**Statistical Considerations:**	Sample size was determined by power analysis (G-Power 3.1, repeated-measures ANOVA with between factor, alpha error probability = 0.05, power = 0.95) based on effect sizes reported by Bogenschutz et al. (2015) revealing a minimum sample size per treatment arm of n=24. Therefore, the planned sample size is 30 participants per treatment arm.<br>Comparisons between psilocybin and placebo will be performed using a mixed-model analysis of variance (ANOVA)/two-sample t-tests to compare primary and secondary endpoint change from baseline at the different time points (1, 14, 28 days post-treatment). Results will be Bonferroni corrected for the number of comparisons.<br>Pilot study data will not be statistically analysed.
**GCP Statement:**	This study will be conducted in compliance with the protocol, the current version of the Declaration of Helsinki, the ICH-GCP or ISO EN 14155 (as far as applicable) as well as all national legal and regulatory requirements.

ABBREVIATIONS

AE	Adverse Event
BASEC	Business Administration System for Ethical Committees, (https://submissions.swissethics.ch/en/)
CA	Competent Authority (e.g. Swissmedic)
CEC	Competent Ethics Committee
CRF	Case Report Form
ClinO	Ordinance on Clinical Trials in Human Research *(in German: KlinV, in French: OClin, in Italian: OSRUm)*
eCRF	Electronic Case Report Form
fMRI	Functional Magnetic Resonance Imaging
DSUR	Development safety update report
DTI	Diffusion Tensor Imaging
GCP	Good Clinical Practice
IB	Investigator's Brochure
Ho	Null hypothesis
H1	Alternative hypothesis
HRA	Federal Act on Research involving Human Beings *(in German: HFG, in French: LRH, in Italian: LRUm)*
IMP	Investigational Medicinal Product
ISF	Investigator Site File
IIT	Investigator-initiated Trial
ISO	International Organisation for Standardisation
ITT	Intention to treat
PI	Principal Investigator
SDV	Source Data Verification
SOP	Standard Operating Procedure
SPC	Summary of product characteristics
SUSAR	Suspected Unexpected Serious Adverse Reaction
TMF	Trial Master File
5-HT	Serotonin

STUDY SCHEDULE

Flow Chart of Study Schedule

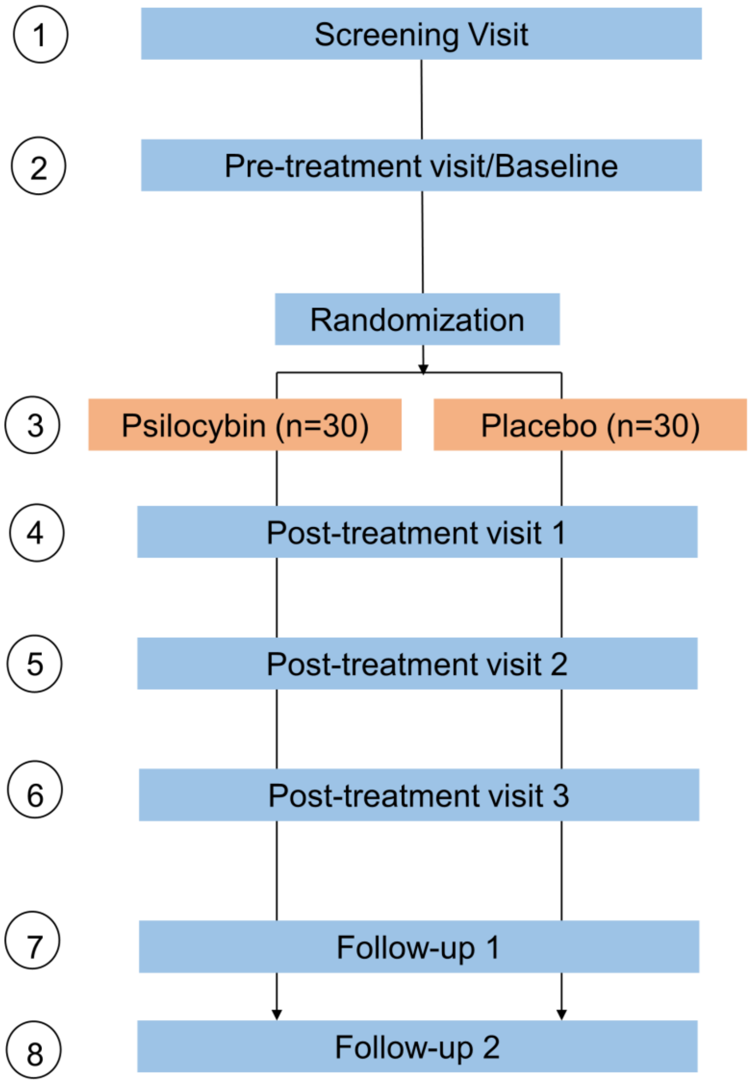

Tabular Listing of Study Schedule

Study Periods	Screening/Pre-treatment Visits		Treatment Period	Post-treatment Visits			Follow-up surveys	
Visit	1	2	3	4	5	6	7	8
Time	t0-14d +/-5d	t0-5d +/-3d	t0	t0+1d	t0+14d +/- 5d	t0+28d +/- 3d	t0+90d +/- 10d	t0+180d +/- 15d
Patient Information and Informed Consent	x							
Demographics, Medical/Psychiatric History, including Family History	x							

Breath Sample for Blood Alcohol Content	x	x	x	x	x	x		
Time-Line Follow Back	x	x	x		x	x	x	x
Alcohol Self-Efficiency Scale	x	x		x	x	x	x	x
Penn Alcohol Craving Scale	x	x	x	x	x	x	x	x
Quality of Life Scale	x	x		x	x	x	x	x
Drinker Inventory of Consequences	x					x	x	x
Beck Depression Inventory	x	x		x	x	x	x	x
Hamilton Anxiety Scale	x	x		x	x	x	x	x
Stress Questionnaire	x					x	x	x
Symptom Checklist	x			x	x	x	x	x
Positive and Negative Mood Schedule	x	x	x	x	x	x	x	x
Structured Clinical Interview (SCID I and II)	x							
Diagnosis of Alcohol Use Disorder Severity based on DSM-5	x							
Verbal IQ (MWT-B)	x							
NEO-FFI	x						x	x
In- /Exclusion Criteria	x							
MRI Safety Form	x	x	x			x		
Physical Examination	x							
Echocardiogram	x							
Vital Signs	x	x	x	x	x	x		
Body Weight	x							
Concomitant Medication Form	x	x	x	x	x	x	x	x
Drug Use Questionnaire	x	x	x	x	x	x	x	x
Drug Urine Test	x	x	x			x		
Routine Laboratory Tests[1]	x							
Laboratory Tests for Alcohol Use Markers[2]	x					x		
Urine Pregnancy Test[3] (Women only)	x	x	x			x		
Randomization		x						
Study Medication Administration			x					
fMRI Resting-State		x	x			x		
fMRI Cue-Reactivity		x	x			x		
fMRI Autobiographic Memory		x	x			x		
Multifaceted Empathy Test		x		x		x		
Altered States of Consciousness Questionnaire			x					

Psychological Support (tape recorded) and BRENDA program		x	x	x	x	x		
Stundenbogen (STEPP, STEPT)		x		x	x	x		
(Epi)Genetics		x		x		x		
Induced pluripotent stem cell		x						
Frankfurt Self-Concept Scale		x		x	x	x	x	x
Emotion-Regulation Questionnaire		x		x	x	x	x	x
Snaith-Hamilton-Pleasure Scale		x		x	x	x	x	x
Savoring Beliefs Inventory		x		x	x	x	x	x
Temporary Experience of Pleasure		x		x	x	x	x	x
Hopelessness Scale		x		x	x	x	x	x
Adverse Events	x	x	x	x	x	x		
Therapy Questionnaire		x					x	x

[1]Hemoglobin, white blood cell count (WBC), Red blood cell count (MCV, MCH), Blood clotting (INR), sodium (Na), potassium (K), calcium (Ca), glucose, creatinine, thyroid stimulating hormone (TSH)

[2] Ethylglucuronid, aspartate aminotransferase (AST), alanine aminotransferase (ALT), gamma-glutamyltransferase (GGT)

[3]human corionic gonadotropin (hCG)

1. STUDY ADMINISTRATIVE STRUCTURE

1.1 Sponsor

Prof. Dr. med. Franz X. Vollenweider
Department of Psychiatry, Psychotherapy and Psychosomatics
Neuropsychopharmacology and Brain Imaging
Psychiatric Hospital, University of Zurich
Lenggstrasse 31
CH-8032 Zürich,
Switzerland
E-mail: vollen@bli.uzh.ch
Phone: +41 58 384 24 04

1.2 Principal Investigator (PI)

PD Dr. med. Marcus Herdener
Department of Psychiatry, Psychotherapy and Psychosomatics
Center for Addiction Disorders
Selnaustrasse 9
CH-8001 Zürich,
Switzerland
E-mail: marcus.herdener@bli.uzh.ch
Phone: +41 44 205 58 11

Dr. F.X. Vollenweider is employed by the Psychiatric Hospital, University of Zurich with a workload of 40%. As Sponsor, he takes the responsibility for the initiation, management, and financing of the clinical trial. Due to this reduced workload, PD Dr. med. Marcus Herdener takes PI responsibility and will ensure the conduction of the study in a timely and correct manner. PD Dr. med. Marcus Herdener has extensive experience with patients with AUD and clinical trials.

1.3 Investigators

Dr. med. Raoul Bitar
Department of Psychiatry, Psychotherapy and Psychosomatics
Centre for Addictive Disorders, Special Ward E1 for Dual Diagnoses
Psychiatric Hospital, University of Zurich
Lenggstrasse 31
CH-8032 Zürich,
Switzerland
E-mail: raoul.bitar@pukzh.ch
Phone: +41 58 384 33 77
Role: Dr. med. Raoul Bitar will be the study physician involved in all aspects of recruitment, screening, treatment, data collection and analysis.

Dr. med. Christina Rossgoderer Pinto da Silva
Department of Psychiatry, Psychotherapy and Psychosomatics
Centre for Depression, Anxiety Disorders and Psychotherapy
Psychiatric Hospital, University of Zurich
Lenggstrasse 31
CH-8032 Zürich,
Switzerland
E-mail: christina.rossgoderer@pukzh.ch
Phone: +41 58 384 23 29
Role: Dr. med. Christina Rossgoderer Pinto da Silva will be the second study physician involved in all aspects of recruitment, screening, treatment, data collection and analysis.
Pract. med. Simon Halm

Department of Psychiatry, Psychotherapy and Psychosomatics
Centre for Addictive Disorders,
Selnaustrasse 9
CH-8001 Zürich
E-Mail: simon.halm@pukzh.ch
Phone: +41 44 205 58 60
Role: Pract. med. Simon Halm will be the third study physician involved in all aspects of recruitment, screening, treatment, data collection and analysis.

M.Sc. Nathalie Rieser
Department of Psychiatry, Psychotherapy and Psychosomatics
Neuropsychopharmacology and Brain Imaging
Psychiatric Hospital, University of Zurich
Lenggstrasse 31
CH-8032 Zürich,
Switzerland
E-mail: nathalie.rieser@bli.uzh.ch
Phone: +41 58 384 33 24
Role: M.Sc. Nathalie Rieser will be an investigator in this study involved in the organization of the clinical trial, recruitment, screening, patient support, data collection and analysis.

M.Sc. Robin von Rotz
Department of Psychiatry, Psychotherapy and Psychosomatics
Neuropsychopharmacology and Brain Imaging
Psychiatric Hospital, University of Zurich
Lenggstrasse 31
CH-8032 Zürich,
Switzerland
E-mail: robin.vonrotz@bli.uzh.ch
Phone: +41 58 384 26 16
Role: M.Sc. Robin von Rotz will be an investigator in this study involved in the organization of the clinical trial, recruitment, screening, patient support, data collection and analysis.

Dr. med. Andres Ort
Department of Psychiatry, Psychotherapy and Psychosomatics
Neuropsychopharmacology and Brain Imaging
Psychiatric Hospital, University of Zurich
Lenggstrasse 31
CH-8032 Zürich,
Switzerland
E-mail: andres.ort@bli.uzh.ch
Phone: +41 58 384 26 03
Role: Dr. med. Andres Ort will support patient screening and provide additional medical support in case of emergencies.

1.4 Statistician ("Biostatistician")

PD Dr. Katrin Preller
Department of Psychiatry, Psychotherapy and Psychosomatics
Neuropsychopharmacology and Brain Imaging
Psychiatric Hospital, University of Zurich
Lenggstrasse 31
CH-8032 Zürich,
Switzerland
E-mail: preller@bli.uzh.ch
Phone: +41 58 384 24 18
Role: PD Dr. Katrin Preller will be our external advisor and statistician

1.5 Laboratory

1.5.1 On-site laboratory

Albina Nowak, PD Dr. (Head)
University Hospital of Psychiatry
Medical Centre/ Laboratory
Lenggstrasse 31
CH-8032 Zürich,
Switzerland
Phone +41 (0)58 384 23 21

Our on-site laboratory will analyse routine laboratory tests, drug urine test, and pregnancy test. These laboratory tests are used for medical screening and study protocol adherence (drug urine repeat) purposes. All samples taken will be immediately destroyed after analysis. Routine laboratory tests include the following parameters:

- Hemoglobin
- Red blood cell count (MCV, MCH)
- Blood clotting (INR)
- white blood cell count (WBC)
- sodium (Na)
- potassium (K)
- calcium (Ca)glucose
- creatinine
- thyroid stimulating hormone (TSH)

Additionally, our on-site laboratory will analyse alcohol use markers at visit 1 and 6. All samples taken will be immediately destroyed after analysis. The following parameters will be assessed to monitor changes is alcohol use behaviour:

- Ethylglucuronid
- aspartate aminotransferase (AST)
- alanine aminotransferase (ALT)
- gamma-glutamyltransferase (GGT)

1.5.2 Collaborating laboratory

PD Dr. Stephanie Witt
Head of the Molecular Genetic Laboratory and Biobank
Dept. of Genetic Epidemiology in Psychiatry
Central Institute of Mental Health
D-68159 Mannheim
Phone +49621 1703-6056
Email: stephanie.witt@zi-mannheim.de

Prof. Dr. Markus Nöthen
Life&Brain GmbH
Venusberg-Campus 1
Gebäude 76
D-53127 Bonn
Phone +49228 6885100
Email: markus.noethen@uni-bonn.de

Our collaborating laboratories will conduct genome-wide genetic analyses to investigate the association between gene variants and treatment outcomes. Genome-wide changes in epigenetic markers of treatment response will be analysed before and after psilocybin administration (visit 2, 4, and 6). Furthermore, to investigate the *in vitro* neuronal profile of psilocybin in alcohol dependent individuals

blood cells (induced pluripotent stem cells) before psilocybin administration (visit 2) will be differentiated into cortical neurons (see 5.2 for details). These analyses are unrelated to any safety issues for this study.

1.6 Monitoring institution

M.Sc. Monika Visentini Gretler
Psychiatric Clinical Trial Center
University Hospital of Psychiatry Zurich
Lenggstrasse 31
CH-8032 Zürich
Switzerland
Phone: +41 (0)58 384 26 07
E-mail: monika.visentinigretler@bli.uzh.ch

Due to the new indication for psilocybin and the vulnerable population an intermediate risk-based monitoring (risk category K2) is appropriate. All source has to be available for monitoring. The monitor will review 100% of the written informed consents, SAEs, the drug accountability and source data verification of the first participant as well as further key data of the following participants. The Monitoring will be performed by the study manger (Monika Visentini) of the psychiatric Clinical Trials Center (psCTC) of the Psychiatric University Hospital Zürich and will occur on-site before, during and after the trial. This will help to follow up the progress of the clinical study, to assure uttermost accuracy of the data and to detect possible errors at an early time point. A Monitoring plan is listed in a separate document. A written report will be submitted to the Sponsor after each trial-site visit by the monitor.

1.7 Data Safety Monitoring Committee

DSMC is not needed, as the drug is in clinical phase II and drug safety is sufficiently well known. Also, in this small, monocentric study, issues with safety would be brought to the sponsor's and PI attention immediately and would be followed up if necessary.

1.8 Any other relevant Committee, Person, Organisation, Institution

This project is funded under the framework of Neuron Cofund. Three independent animal studies will address complementary mechanistic questions in mice.

These studies are led by:

Rainer Spanagel, PhD
Central Institute of Mental Health (CIMH; Zentralinstitut für Seelische Gesundheit)
Institute for Psychopharmacology
Heidelberg University, Germany

Roberto Ciccocioppo
Pharmacology Unit
School of Pharmacy,
University of Camerino, Italy

Mickael Naassila
Department of Clinical Pharmacology &
Evidence-Based Medicine
INSERM U1247 Research Group on
Alcohol & Pharmacodependences (GRAP)
Université de Picardie Jules Verne, France

These research projects are independent and the research groups have no role in study design, data

collection or analysis of the current study.

2. ETHICAL AND REGULATORY ASPECTS

Before the study will be conducted, the protocol, the proposed patient information and consent form as well as other study-specific documents shall be submitted to a properly constituted Competent Ethics Committee (CEC) and competent authorities (Swissmedic/BAG) in agreement with local legal requirements, for formal approval. Any amendment to the protocol must as well be approved (if legally required) by these institutions.

The decision of the CEC and Swissmedic/foreign competent authority concerning the conduct of the study will be made in writing to the Sponsor before commencement of this study. The clinical study can only begin once approval from all required authorities has been received. Any additional requirements imposed by the authorities shall be implemented.

2.1 Study registration

The study will be registered in the international trial registry ClinicalTrials.gov (clinicaltrials.gov). Additionally, the study will be registered in a national language in the Swiss National Clinical Trial Portal (SNCTP via BASEC).

2.2 Categorisation of study

This clinical trial with IMP comes under the Category C (ClinO Art. 19) because psilocybin is not authorized as a medical product in Switzerland.

2.3 Competent Ethics Committee (CEC)

Approval from the appropriate constituted Competent Ethics Committee is sought for each study site in the clinical trial. The reporting duties and allowed time frame are respected. No substantial changes are made to the protocol without prior Sponsor, CEC, CA approval, except where necessary to eliminate apparent immediate hazards to study participants.

Premature study end or interruption of the study is reported within 15 days. The regular end of the study is reported to the CEC within 90 days, the final study report shall be submitted within one year after study end. Amendments are reported according to chapter 2.10.

2.4 Competent Authorities (CA)

The Sponsor will obtain approval from Swissmedic and the Bundesamt für Gesundheit (BAG) before the start of the clinical trial. Reporting will be done within the allowed time frame.

Planned or premature study end are reported within 90 and 15 days, respectively. The final report will be submitted to the CA within one year after the end of the study. Amendments are reported according to chapter 2.10.

2.5 Ethical Conduct of the Study

The study will be carried out in accordance to the protocol and with principles enunciated in the current version of the Declaration of Helsinki, the guidelines of Good Clinical Practice (GCP) issued by ICH, the Swiss Law and Swiss regulatory authority’s requirements. The CEC and regulatory authorities will receive annual safety and interim reports and be informed about study stop/end in agreement with local requirements.

2.6 Declaration of interest

All investigators declare no conflict of interest.

2.7 Patient Information and Informed Consent

The investigators will explain to each participant the nature of the study, its purpose, the procedures involved, the expected duration, the potential risks and benefits and any discomfort it may entail. Each participant will be informed that the participation in the study is voluntary and that he/she may withdraw from the study without providing any reason at any time and that withdrawal of consent will not affect his/her subsequent medical assistance and treatment.

The participant must be informed that his/her medical records may be examined by authorised individuals other than their treating physician.

All participants for the study will be provided a participant information sheet and a consent form describing the study and providing sufficient information for participant to make an informed decision about their participation in the study. Before participants make their final decision on study participation, they must have a reasonable amount of time for consideration.

The formal consent of a participant, using the approved consent form, must be obtained before the participant is submitted to any study procedure.

The participant should read and consider the statement before signing and dating the informed consent form, and should be given a copy of the signed document. The consent form must also be signed and dated by the investigator (or his designee) at the same time as the participant sign, and it will be retained as part of the study records. The informed consent can be withdrawn by the participant at any time without explanation. Data collected up to this point will be analysed.

Prior to their enrolment, participants will be asked for consent concerning the potential re-use of their data. This serves to maximize the scientific value of the acquired data and to avoid unnecessary repetitions of measurements, thus reducing the burden on the participants should they wish to take part in future studies. The informed consent can be withdrawn by the participant at any time without explanation. Data collected up to this point will be analysed.

Patients will not be compensated for their participation. However, costs for travel to the study site and meals at the study site will be reimbursed.

2.8 Participant privacy and confidentiality

The investigator affirms and upholds the principle of the participant's right to privacy and that they shall comply with applicable privacy laws. Especially, anonymity of the participants shall be guaranteed when presenting the data at scientific meetings or publishing them in scientific journals.

Individual subject medical information obtained as a result of this study is considered confidential and disclosure to third parties is prohibited. Subject confidentiality will be further ensured by utilising subject identification code numbers to correspond to treatment data in the computer files.

For data verification purposes, authorised representatives of the Sponsor, a competent authority (e.g. Swissmedic), or an ethics committee may require direct access to parts of the medical records relevant to the study, including participants' medical history

2.9 Early termination of the study

The Sponsor may terminate the study prematurely according to certain circumstances, for example:

- ethical concerns,
- insufficient participant recruitment,
- when the safety of the participants is doubtful or at risk, respectively,
- alterations in accepted clinical practice that make the continuation of a clinical trial unwise,
- early evidence of benefit or harm of the experimental intervention

2.10 Protocol amendments

The Sponsor (Prof. Dr. Franz Vollenweider) and the Principal Investigator, PD Dr. med. Marcus Herdener, are allowed to amend the protocol or to provide suggestions for a protocol amendment.

Substantial amendments are only implemented after approval of the CEC and CA respectively. Important protocol modifications (e.g., changes to eligibility criteria, outcomes, analyses) will be communicated to all relevant parties (e.g., investigators, CEC, competent authorities, trial participants, trial registries, journals, regulators), if applicable.

Under emergency circumstances, deviations from the protocol to protect the rights, safety and well-being of human subjects may proceed without prior approval of the sponsor and the CEC/CA. Such deviations shall be documented and reported to the sponsor and the CEC /CA as soon as possible.within 7 days.

All non-substantial amendments are communicated to the CA as soon as possible if applicable and to the CEC within the Annual Safety Report (ASR).

3. BACKGROUND AND RATIONALE

3.1 Background and Rationale

Alcohol use disorders (AUDs) create the largest health burden globally

Two billion people globally consume alcohol, leading in 2016 to 2.8 million deaths (5.2% of all deaths) and 99.2 million Disability Adjusted Life Years (DALYs) lost (4.2% of all DALYs) (Collaborators, 2017). Of all the diseases, conditions, and injuries attributable to alcohol use, alcohol use disorders (AUDs) create the largest health burden globally (Collaborators, 2017). AUDs are diagnostically characterized in the Diagnostic and Statistical Manual of Mental Disorders (DSM5) by a strong desire or compulsion to drink alcohol despite knowledge or evidence of its harmful consequences, and by difficulty in controlling drinking in terms of onset, termination or levelling of its use, physiological withdrawal symptoms, and development of tolerance. People who fulfil these criteria are addicted to alcohol and 23 million Europeans are suffering from alcohol addiction (Rehm et al., 2015).

Limited effectiveness of approved treatments

Currently, disulfiram (Antabuse®), naltrexone, and acamprosate (Campral®) have been approved by most regulatory bodies for the treatment of alcohol addiction and are available in most countries across the globe (Spanagel & Vengeliene, 2013). Nalmefene (Selincro®) has recently been approved for the reduction of alcohol consumption. However, these approved pharmacological treatments for alcoholism are limited in their effectiveness (Litten, Falk, Ryan, Fertig, & Leggio, 2018), and new drugs that can be translated into the clinic are warranted. However, drug development for alcohol dependence is highly challenging due to methodological issues of alcohol pharmacotherapy trials (Litten et al., 2018; Yardley & Ray, 2017) as well as placebo responses. The placebo response can vary across trials and is negatively correlated with the treatment effect size (Litten et al., 2013). Furthermore, several novel mechanisms and compounds that appeared to hold great promise based on preclinical data failed to translate to the human condition (Litten et al., 2018).

The rationale to study the efficacy of psilocybin to treat alcohol addiction and the underlying neurobiology

Here we propose to test the efficacy of psilocybin for treating alcohol addiction and study its underlying neurobiological mechanisms. Psilocybin is a prodrug and is rapidly dephosphorylated to psilocin, which acts as a partial agonist primarily at serotonin (5-HT) 2A receptors in humans (Kometer, Schmidt, Jancke, & Vollenweider, 2013; Preller & Vollenweider, 2018). Fifty years ago there was extensive research on hallucinogens in the treatment of addiction. However, due to the misuse of hallucinogens during the counterculture movement these substances could not further be developed as medicinal products (Vollenweider & Kometer, 2010). The past decade has seen a renaissance of research in potential clinical applications of the classic hallucinogen psilocybin. In healthy participants, it has been shown that the administration of psilocybin was related to long-lasting positive effects on participants' relationship to nature, and appreciation of aesthetic experiences, as well as to increases in the personality openness (Preller & Vollenweider, 2018). Furthermore, in clinical populations, only one or two administrations of psilocybin have been shown to significantly and long-lastingly (i.e., up to six months) reduce symptoms in randomized controlled trials with terminally ill patients suffering from depression and anxiety due to cancer, as well as in proof-of-concept, safety and tolerability studies in patients suffering from major depression, OCD and nicotine dependence (Carhart-Harris et al., 2016; Griffiths et al., 2016; M. W. Johnson, Garcia-Romeu, Cosimano, & Griffiths, 2014; Moreno, Wiegand, Taitano, & Delgado, 2006; Ross et al., 2016).

Importantly, a recent proof-of-concept study testing psilocybin in ten alcohol dependent patients (Bogenschutz et al., 2015) provides encouraging efficacy results (**Fig. 1**) and safety data. Participants exhibited significant improvement in drinking, with large pre–post effect sizes, as well as significant changes in psychological measures relevant to drinking after the administration of psilocybin (0.3 mg/kg and 0.4mg/kg). No significant treatment-related adverse events were recorded (Bogenschutz et al., 2015). These data are in line with the results of online surveys assessing changes in alcohol consumption following psychedelic use in non-clinical settings showing that psychedelic use may lead to cessation or reduction in problematic alcohol use (Garcia-Romeu et al., 2019). Together, these beneficial efficacy, safety, and tolerability results provide a rationale for a controlled trial with larger samples to investigate efficacy (primary purpose) and the mode of action (secondary purpose) of psilocybin for the treatment of alcohol use disorder.

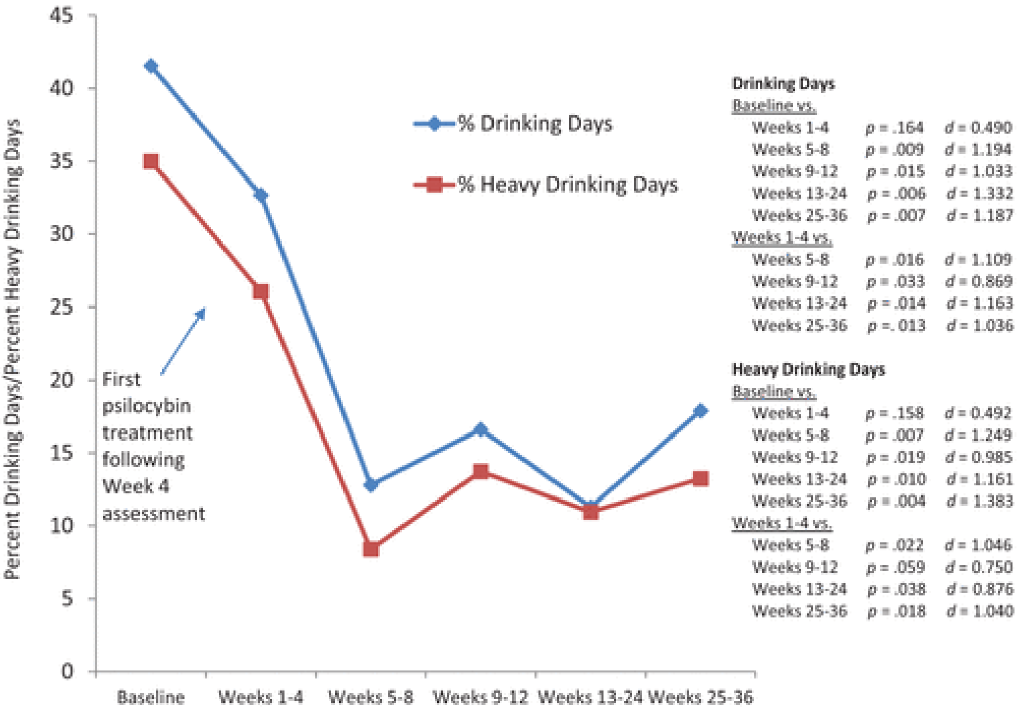

Figure 1. Drinking outcomes and effect sizes after the administration of psilocybin (N=10). From Bogenschutz et al., (2015).

Primary research question: Does psilocybin reduce relapse after alcohol withdrawal?

Our first main objective is to study the efficacy of psilocybin on relapse behaviour in alcohol dependent patients who recently underwent alcohol withdrawal. Relapse rates are reported to be as high as 80 – 92 % after treatment for alcohol addiction and therefore represent a major threat to treatment efficacy (Brandon, Vidrine, & Litvin, 2007). As outlined above, the beneficial efficacy, safety, and tolerability results presented in previous proof-of-concept studies (Bogenschutz et al., 2015), provide a rationale for a controlled trial with larger samples to investigate efficacy of psilocybin on relapse behaviour in alcohol addiction.

Secondary research question: What are the mechanisms underlying psilocybin's potential efficacy in relapse prevention in alcohol dependent patients?

Despite the urgent need of novel treatment approaches, the development of novel medication in psychiatry has stagnated for decades. The main reason for this is the lack of biological understanding of psychiatric disorders and the lack of understanding of the biological mechanisms underlying pharmacological treatment response (Abi-Dargham & Horga, 2016; Doyle, Mehta, & Brammer, 2015; Insel et al., 2013). Therefore, we will address this question by investigating the changes in brain connectivity and reactivity via functional magnetic resonance imaging (fMRI) before, during, and after psilocybin administration. We and others have shown that psilocybin reduced the processing of negative stimuli, while at the same time enhances connectedness and empathy, as well as in the long-term increases the participants' relationship to nature, appreciation of aesthetic experiences, and the personality openness (Kraehenmann et al., 2015; Kraehenmann et al., 2016; Pokorny, Preller, Kometer, Dziobek, & Vollenweider, 2017; Preller et al., 2016; Preller & Vollenweider, 2018). Furthermore, it has been shown that psychedelics increase prefrontal top-down control over limbic regions and alters connectivity between the ventral striatum and cortical brain areas (Preller et al., 2018; Preller, Razi, et al., 2019). It is therefore conceivable that altered reward processing, in particular a reduced processing of negative stimuli including drug cues combined with reinstatement of drug-unrelated rewards such as social interaction, underlies the potential therapeutic effect of psilocybin in addiction disorders.

Besides brain network and reactivity restoration, epigenetic effects are a possible candidate mechanism underlying psilocybin's potential efficacy. Persistent alterations in gene expression following compulsive alcohol consumption imply the importance of epigenetic mechanisms in alcohol addiction (Spanagel,

2009). In particular, histone modifications and DNA methylation are important regulators of gene expression and epigenetic signatures of brain regions critical to reward processing and alcohol addiction in both animal and human studies have already been described (Berkel & Pandey, 2017). Interestingly, activation of the 5-HT_{2A} receptor - the primary molecular site of action of psilocin - represses the promoter activity of the histone deacetylase 2 gene in mouse and in human frontal cortex (Holloway & Gonzalez-Maeso, 2015). This finding and the fact that a single application of psilocybin has long-lasting effects on disease trajectories and quality of life (Kyzar, Nichols, Gainetdinov, Nichols, & Kalueff, 2017) provide a rationale for studying epigenetic mechanisms that may underlie psilocybin's potential efficacy. This project is funded under the framework of Neuron Cofund, therefore, three independent animal studies will address complementary mechanistic questions in mice. These research projects are independent and the research groups have no role in study design, data collection or analysis of the current study. A pilot study will be conducted to evaluate the feasibility of fMRI paradigms in this study.

3.2 Investigational Product and Indication

Investigational Product Name: Psilocybin
Chemical Name: 3-(2-(dimethylammonio)ethyl)-1H-indol-4-yl hydrogen phosphate
Synonyms: CY-39, Indocybin®, CHEBI:8614
Chemical Family: zwitterionic alkaloid
Molecular Weight: 284.25 g/mol
Chemical Formula: C12H17N2O4P
Form: crystalline powder
Color: white to off-white
Odor: odorless
Physical and Chemical Properties:
PH: 5,2 in 50 % aq. ethanol
Melting Point: 210°C-230°C
Solubility: soluble in water and methanol, not soluble in non-polar organic solvents
Stability: stable under standard conditions (tightly closed at 2°C to 8°C)

Psilocybin is the major of two hallucinogenic components of Teonanacatl, the sacred mushroom of Mexico. Psilocybin was first isolated from psilocybe mushrooms by Hofmann in 1957, and later synthesized by him in 1958 (Passie, Seifert, Schneider, & Emrich, 2002). Soon after, synthetic psilocybin was marketed by Sandoz under the name Indocybin® for basic psychopharmacological and therapeutic clinical research (Vollenweider & Kometer, 2010). Psilocybin is a potent 5-HT_{2A} agonist that has demonstrated safety and tolerability in both preclinical and clinical studies (Tyls, Palenicek, & Horacek, 2014). For details, see the Investigator's Brochure.

3.3 Preclinical Evidence

Psilocybin was marketed by Sandoz under the name Indocybin® for basic pharmacological and clinical research (Vollenweider & Kometer, 2010). Psilocybin belongs to a class of drugs referred to as hallucinogens or psychedelics. Specifically, it is a serotonergic hallucinogen, along with other tryptamines such as dimethyltryptamine (DMT), ergolines, such as LSD and phenethylamines, such as mescaline. Currently, research in human and nonhuman animals suggests that serotonergic hallucinogens produce most of their effects as a result of 5-HT_{2A} agonism, with contributions also from agonism at $5HT_{2C}$ and $5HT_{1A}$ receptors (Nichols, 2004). Like other members of this class, acute administration of psilocybin induces profound changes in perception, cognition and emotion during 4-6 hours after oral intake. Psilocybin was used in psychiatric and psychological research and as a valuable adjunct in psychodynamic-oriented psychotherapy during the early to mid-1960s up until its scheduling in 1970 in the US (Leuner, 1982). Research into the effects of psilocybin resumed in the mid-1990s, and it is currently the preferred compound for use in studies of the effects of serotonergic hallucinogens (Baumeister, Barnes, Giaroli, & Tracy, 2014). Since 1990, our and other laboratories have conducted more than 50 preclinical and clinical studies investigating the neurocognitive and emotional effects of psilocybin (Carhart-Harris, Erritzoe, et al., 2012; Carhart-Harris, Leech, et al., 2012; Kometer et al., 2013; Kraehenmann et al., 2015; Lewis et al., 2017; Pokorny et al., 2017; Preller et al., 2016; Umbricht et al., 2003). We and others have shown that psilocybin reduced the processing of negative stimuli, while at the same time enhances connectedness and empathy, as well as in the long-term increases the participants' relationship to nature, appreciation of aesthetic experiences, and the personality

openness (Kraehenmann et al., 2015; Kraehenmann et al., 2016; Pokorny et al., 2017; Preller et al., 2016; Preller & Vollenweider, 2018). Furthermore, it has been shown that psychedelics increase prefrontal top-down control over limbic regions and alters connectivity between the ventral striatum and cortical brain areas (Preller et al., 2018; Preller, Razi, et al., 2019). It is therefore conceivable that altered reward processing, in particular a reduced processing of negative stimuli including drug cues combined with reinstatement of drug-unrelated rewards such as social interaction, underlies the potential therapeutic effect of psilocybin in addiction disorders.

3.4 Clinical Evidence to Date

The majority of clinical studies with psilocybin took place between the mid-1950s and 1960s shortly after its synthesis by the Swiss chemist Albert Hofmann in 1958. Most clinical studies were using Sandoz's Indocybin®, and several thousand scientific papers published by 1965 described positive results in more than 40'000 patients who had taken psychedelics with minimal side effects and a high level of safety (Grinspoon & Bakalar, 1981). Up to now, approximately 2000 subjects have received single or repeated doses of psilocybin for treatment of neurotic disorder, alcohol dependence, autism, and anxiety related to terminal cancer. For example, in a recent clinical studies, psilocybin was given as an anxiolytic and antidepressant in terminally ill cancer patients without clinically significant side effects (Griffiths et al., 2016; Grob et al., 2011; Ross et al., 2016). Clinical trials have also shown that psilocybin might be effective in the treatment of obsessive-compulsive disorder (OCD) and major depression, with therapeutic effects lasting up to six months (Carhart-Harris et al., 2016; Moreno et al., 2006). Furthermore, it has been shown that 12 of 15 participants (80%) were abstinent from smoking based on biological verification after treatment with psilocybin to support smoking cessation (M. W. Johnson et al., 2014).

In the 1950s through early 1970s there was extensive research on the use of hallucinogens, especially of LSD – which is like psilocybin a partial agonist primarily at 5-HT_{2A} receptors - in the treatment of alcohol addiction. A recent meta-analysis (Krebs & Johansen, 2012) examined the six published randomized trials of LSD treatment of alcoholism from that time. A total of 325 participants received active treatment with LSD, and 211 received control treatment. At the first posttreatment follow-up (ranging from 1 month to 12 months) the odds ratio for improvement was 1.96, favoring LSD. This is a highly significant result achieved by a single administration of a hallucinogenic drug. Additionally, online surveys assessing changes in alcohol consumption following psychedelic use in non-clinical settings showed that psychedelic use may lead to cessation or reduction in problematic alcohol us (Garcia-Romeu et al., 2019). Given these promising results, there is now renewed interest in the use of hallucinogens for the treatment of alcohol addiction and especially the application of psilocybin is now in the focus for the development of a medicinal product. For drug development psilocybin has indeed several advantages compared to LSD or other hallucinogens: (i) psilocybin produces very low harm compared to other drugs of abuse, (ii) psilocybin has a shorter half-life than LSD and as such it is more practical for the intervention session; especially for the therapeutic team, (iii) and it has a more distinct pharmacology at serotonin receptors than LSD which has many more targets (Kyzar et al., 2017). A recent single-group proof-of-concept study on psilocybin in ten alcohol addicted patients who received orally administered psilocybin in one or two supervised sessions showed a significant increase in abstinence days (compared to a baseline assessment) which was maintained at follow-up to 36 weeks (**Fig. 1**) (Bogenschutz et al., 2015). Importantly, no significant treatment-related adverse events were recorded (Bogenschutz et al., 2015).

3.5 Dose Rationale

A single dose of 25mg will be used. This dose corresponds to the doses administered in a previous study investigating the effect of psilocybin in alcohol addiction (.3mg/kg and .4mg/kg) and that has been shown to be clinically effective and well tolerated (Bogenschutz et al., 2015).

3.6 Explanation for choice of comparator (or placebo)

This study investigates the effects of psilocybin in patients with alcohol use disorder. Therefore, pharmaceutically inactive placebo is used in a parallel group randomized double-blind study design. All patients entering the trial will be off psychotropic medications for the duration of the trial, in order to

prevent confounding effects by concomitant medication. Because of the relatively short trial duration (5 weeks from study entry till closure visit) and the counselling sessions at each trial visit, adequate and sufficient psychological support is provided for both the active and the comparator group.

3.7 Risks / Benefits

3.7.1 Potential Risks

3.7.1.1 Risks of experimental medications

In this study psilocybin will be used as the putatively active treatment. Psilocybin will be given as a single, moderate dose orally. Psilocybin has been used safely in numerous previous human studies (Tyls et al., 2014). In carefully conducted clinical research settings, with careful screening, preparation and support, the risks of psilocybin administration are low (Bogenschutz et al., 2015; Moreno et al., 2006; Studerus, Kometer, Hasler, & Vollenweider, 2011; Tyls et al., 2014). Classic hallucinogens including psilocybin have the lowest physiological toxicity and lowest dependence potential of all well-known drugs of abuse (Gable, 2004). However, there are some rare but potentially adverse reactions that need to be appropriately addressed to maximize safety. In general, adverse reactions are clearly dose-dependent and mostly occur at higher doses than the dose used in this study (Hasler, Grimberg, Benz, Huber, & Vollenweider, 2004).

- Experiences with hallucinogens may provoke a variety of positive and negative emotional responses following the acute drug experience, which can be unsettling. Anxiety and dysphoria may occur during psilocybin intoxication. These symptoms generally respond well to psychological support and reassurance. A single dose of Temesta® (lorazepam) 1x 1-3mg will be administered orally for severe anxiety.
  - Increased blood pressure and heart rate during intoxication (mean increase of approximately 20 mm Hg systolic, 12 mm Hg diastolic, mean increase in pulse of approximately 10 bpm) (Griffiths, Richards, McCann, & Jesse, 2006). This side effect is common, but unlikely to be clinically significant with careful screening for hypertension and cardiovascular disease. In case of a clinically significant increase in blood pressure (>160 systolic and >100 diastolic), a study doctor will decide (based on anamnesis and clinical aspects), whether a single dose of Adalat retard® (nifedipine) 1x 20mg will be administered orally for hypertension.
- Transient psychotic symptoms (delusions, paranoia, hallucinations) have been reported during psilocybin intoxication. We are unaware of any reports of persistent psychotic symptoms associated with the use of psilocybin, but such symptoms have been reported following use of LSD (Preller & Vollenweider, 2018). Individuals with history of psychosis or vulnerability (e.g., family history of schizophrenia) are thought to be at higher risk for psychotic reactions to hallucinogens and will be excluded. A single dose of Zyprexa® (Olanzapine) 1x 5-10 mg will be administered orally for psychotic symptoms that pose a significant danger.
- Hallucinogen persisting perception disorder (HPPD, “flashbacks”) can be caused by hallucinogen use. The incidence of this disorder is thought to be very low, and associated primarily with use of LSD (Halpern & Pope, 2003). To date, however, no cases of HPPD have occurred in volunteers given psilocybin in current research studies (Studerus et al., 2011). The risk of HPPD occurring after psilocybin administration can be reduced by screening participants through excluding people reporting HPPD or other significant adverse events after prior use of hallucinogens, as done in this study.
- Headaches are common following psilocybin administration (M. Johnson, Richards, & Griffiths, 2008; Studerus et al., 2011). The headaches are not severe, resolve within a day, and respond to over-the-counter pain medications (M. Johnson et al., 2008).
- Psilocybin, like other classic hallucinogens, is an abusable drug. It is theoretically possible that a participant could develop a pattern of psilocybin misuse due to his/her positive experience in the trial, although this has not been reported in previous studies in which psilocybin or other classic hallucinogens have been administered to humans (Studerus et al., 2011). Psilocybin has the lowest dependence potential of all well-known drugs of abuse (Gable, 2004).

There is considerable clinical experience with psilocybin over more than 50 years, including its use in randomized controlled trials with terminally ill patients suffering from depression and anxiety due to cancer, as well as in proof-of-concept, safety and tolerability studies in patients suffering from major

depression, OCD, nicotine and alcohol dependence (Bogenschutz et al., 2015; Carhart-Harris et al., 2016; Griffiths et al., 2016; M. W. Johnson et al., 2014; Moreno et al., 2006; Ross et al., 2016). Few safety issues were noted in all these studies. Importantly, no significant treatment-related adverse events were recorded in the previous study investigating the effects of psilocybin in alcohol-dependent participants (Bogenschutz et al., 2015).

3.7.1.2 Risks of rescue medications

Use of these medications is expected to occur uncommonly if at all in this study. Acute adverse reactions to psilocybin initially will be managed by increasing psychological support ("talking down"). Rescue medication will be available for onetime administration if needed at all:

- Adalat retard® (nifedipine) 1x 20mg administered orally for hypertension in case of a clinically significant increase in blood pressure (>160 systolic and >100 diastolic), which will be decided by a study doctor (based on anamnesis and clinical aspects) as a single dose. Common side effects include headache, constipation, vasodilatation, and edema
- Temesta® (lorazepam) 1x 1-3mg administered orally for severe anxiety. The most common effects are sedation, dizziness, weakness, and unsteadiness. Rare but serious adverse events that could occur with a single administration include respiratory depression, apnea, and anaphylaxis.
- Zyprexa® (olanzapine) 1x 5-10 mg administered orally for psychotic symptoms (delusions, hallucinations, disorganized behaviour) that pose a significant danger. Common side effects are somnolence, dizziness, hypotension, akathisia, extrapyramidal symptoms, dry mouth, tremor, nausea, and vomiting. Rare but serious side effects include seizures, neuroleptic malignant syndrome and tardive dyskinesia (the latter two are extremely unlikely with one or two small doses)

3.7.1.3 Risks of blood draws

For most people, needle punctures for blood draws do not cause any serious problems. It is a routine medical procedure. However, in rare cases, local haematoma or infection can occur, which do not pose serious health risks. Blood draws will not exceed 18 ml per visit. The following amounts of blood will be collected at the respective visits:

Visit 1: 11 ml (in- and exclusion criteria and markers of alcohol use)
Visit 2: 18 ml (genetics, epigenetics, and induced pluripotent stem cells)
Visit 4: 10 ml (epigenetics)
Visit 6: 13.5 ml (epigenetics and markers of alcohol use)

3.7.1.4 Risks of assessment procedures

There are no known psychological risks associated with the questionnaires used in the study, all of which have been used extensively in clinical populations. It is possible that discussion of biographical history, significant life events, relationship issues, substance use, and psychiatric symptoms may cause emotional discomfort in some participants. To minimize such discomfort, the following steps will be taken. The consent form will fully inform the participants about the nature of the information to be disclosed in the protocol, and the participants will be informed in the consent form that they can refuse to answer any questions or withdraw from the study at any time. Participants will be informed that all information is confidential. One of the investigators of the project will be available to meet with any participant who becomes distressed about any aspect of the protocol and wishes to discuss this.

3.7.1.5 Risks of functional magnetic resonance imaging (fMRI)

The fMRI investigation is a modern, multi-tested and established non-invasive technique which uses specifically designed equipment. According to current knowledge, the fMRI investigation does not bear any health risks. Although the fMRI investigation does not expose subjects to radiation, metal parts or electronic implants in the body may bear a safety risk because of the electromagnetic field in the scanner and are therefore contraindicated for this investigation (an MRI safety screening will be performed before

each scanning session). Scanners with 3.0Tesla field strength have been routinely being used in hospitals for many years. To date, no adverse health effects have occurred. Still, subjects should avoid abrupt movements whilst lying in the scanner because this could temporarily induce dizziness or metallic taste in the mouth. Occasionally, subjects may briefly see light flashes ("phosphenes"). For the applied radio waves, limit values similar to mobile phones are applied and are strictly adhered to. Thereby, potential heating of the body is avoided. To protect against knocking noise, all subjects will use appropriate ear protection. During scanning, subjects can communicate with the investigator via intercom at any time. Although no side effects for unborn children have occurred, no fMRI investigation should be performed at healthy, pregnant subjects for research purposes. Therefore, female participants will complete a pregnancy test prior to study inclusion. All participants will be informed about possible risks.

3.7.1.6 Risks to confidentiality

Medical records, the investigator's study related files and correspondence, and the informed consent documentation which identify subjects may be inspected by the competent authority or CEC, respectively for quality assurance purposes. Because of the need to release information to these parties, absolute confidentiality cannot be guaranteed. The results of this research project may be presented at meetings or in publications. However, the identity of individual subjects will not be disclosed in those presentations.

3.7.2 Mitigation of risks

3.7.2.1 Management of risks due to experimental medications

Risks of hallucinogen administration are thought to be minimized by a) careful selection of participants, b) extensive preparation c) presence of qualified study personnel during the psilocybin sessions, d) a safe and reassuring physical environment, and e-g) follow-up for possible residual adverse effects. These preventive measures are incorporated into our protocol as follows (see also Section 8.6):

a) Participant selection: Exclusion criteria are designed to exclude patients who would be at elevated risk for adverse events due to psilocybin. These criteria are listed in Section 7.1., and include both medical exclusions and psychiatric exclusions for serious psychopathology, history of violent or suicidal behaviour, and family history of psychosis.

b) Participant preparation: In addition to the information and discussion provided in the informed consent process during the initial screening visit, participants' will be psychologically prepared by one of the investigators. Preparation will include 1) open-ended questions to establish rapport, learn about the participant's history, belief system, and values, assess motivation and expectations for the study, and discuss any previous experience with hallucinogens; 2) detailed information about the physiological and psychological effects of psilocybin; 3) emphasis that the purpose of the psilocybin sessions is to help overcome alcohol use disorder symptomatology via psilocybin's pharmacological and psychological effects, and discussion of how this could work; 4) advice as to how to deal with dysphoric reactions to psilocybin, should they occur; and 5) discussion of ground rules for the session, including adherence to the protocol (compliance with dietary restrictions, e.g. alcohol, caffeinated drinks, and other psychoactive substances; effective contraception; restricted use of concomitant medication; transport and care by significant others after psilocybin session).

c) One of the investigators will serve as the monitor who will attend and interact with the participant during the psilocybin session. All investigators will be trained by the PI and the sponsor. A physician will always be on site in case of emergencies. After the treatment visit, participants will only be released after having been examined by a physician. Patients will be picked up by a family member or friend who can stay with them over night after the treatment visit. Details on the release procedures are documented in an SOP.

d) The psilocybin session will take place on-site in an environment that is appropriate for hallucinogen administration sessions. To the extent possible, the room used for the session will be specially prepared for the session to provide a warm, quiet and home-like rather than a stark clinical quality because of the large influence that setting can have on the subjective effects of hallucinogens. Patients will be allowed to lie on a couch or to move freely. Interaction with the patients will be supportive and non-directional. To establish a quiet, relaxing, introspective

ambience, no cell phones, laptops, or other electronic devices will be allowed during the session.

e) During treatment sessions, study personnel will be responsible for monitoring participants for possible clinical deterioration or other problems, and for implementing appropriate courses of action.

f) Post-treatment visits will be conducted one day, 14 days (+/- 5 days), and 28 days (+/- 3 days) after substance administration. Standardized preparation before and follow-up after the psilocybin administration sessions is based on the BRENDA Model developed for the treatment of alcohol use disorders (Starosta, Leeman, & Volpicelli, 2006). Furthermore, they will have time to discuss the treatment visit with the investigator. Changes in symptomatology or need for therapeutic interventions will be monitored.

g) An online survey three and six months after the substance administration will ensure monitoring of subtle changes in symptomology and the subjective need for therapeutic interventions over a longer time-course.

3.7.2.2 Minimization of risk to confidentiality

Confidentiality of research material will be ensured by storing the research materials in locked cabinets. Material will be available only to project staff, and only as needed. All project staff will be thoroughly trained in issues relating to confidentiality. Participants will be identified in case report forms (CRFs) by an identification code.

3.7.3 Potential benefits

3.7.3.1 Potential benefits to participants

Participants may or may not experience clinical benefit from this study. Aspects of study participation likely to be beneficial include free medical and psychiatric evaluations, the attention and support of participating in a clinical trial, and standardized psychological support.

3.7.3.2 Risk benefit assessment

Risks to individual participants appear to be balanced by the likely benefits of study participation, and outweighed by the potential benefits to others.

3.7.3.3 Importance of the knowledge to be gained

There is an urgent need to develop more effective methods to help people who suffer from alcohol use disorder. The knowledge gained through this study could point the way to an entirely new treatment for alcohol addiction. As indicated above, these potential benefits (in addition to the potential benefits to individual participants) appear to justify the risks to individual participants.

3.8 Justification of choice of study population

Because this study investigates whether psilocybin has therapeutic efficacy with regard to relapse prevention in participants suffering from alcohol use disorder, the choice of study population is restricted to this clinical population and cannot be obtained from healthy volunteers without compromising study results. Only patients capable of giving informed consent will be included.

4. STUDY OBJECTIVES

4.1 Overall Objective

The overall objective of this study is to test the efficacy and mechanism of the 5-HT$_{2A}$ agonist psilocybin compared to placebo for relapse prevention in adult alcohol addicted patients who recently underwent alcohol withdrawal.

Two billion people globally consume alcohol, leading in 2016 to 2.8 million deaths (5.2% of all deaths) and 99.2 million Disability Adjusted Life Years (DALYs) lost (4.2% of all DALYs) (Collaborators, 2017). Of all the diseases, conditions, and injuries attributable to alcohol use, alcohol use disorders (AUDs) create the largest health burden globally (Collaborators, 2017). Relapse rates are reported to be as high as 80 – 92 % after treatment for alcohol addiction and therefore represent a major threat to treatment efficacy (Brandon et al., 2007). Currently approved pharmacological treatments for alcoholism are limited in their effectiveness and novel mechanisms and compounds that appeared to hold great promise based on preclinical data failed to translate to the human condition (Litten et al., 2018). Thus, there is a high need for studying novel pharmacological compounds that might have clinical efficacy and increasing our understanding of the underlying neurobiological mechanisms.

A recent proof-of-concept study testing psilocybin in ten alcohol dependent patients (Bogenschutz et al., 2015) provides encouraging efficacy results (**Fig. 1**) and safety data. Participants exhibited significant improvement in drinking, with large pre–post effect sizes, as well as significant changes in psychological measures relevant to drinking after the administration of psilocybin. No significant treatment-related adverse events were recorded (Bogenschutz et al., 2015). These data are in line with the results of online surveys assessing changes in alcohol consumption following psychedelic use in non-clinical settings showing that psychedelic use may lead to cessation or reduction in problematic alcohol use (Garcia-Romeu et al., 2019). Together, these preliminary uncontrolled studies provide beneficial efficacy, safety, and tolerability results that warrant the investigation of psilocybin in a randomized controlled trial investigating efficacy (primary purpose) and the mode of action (secondary purpose) of psilocybin for relapse prevention in alcohol use disorder.

4.2 Primary Objective

The study seeks primarily to determine effects of a single dose of psilocybin (25mg) compared to placebo on relapse behaviour in adult alcohol addicted patients who underwent alcohol withdrawal.

4.3 Secondary Objectives

The secondary objective is to determine the mechanisms underlying psilocybin's potential clinical efficacy via the investigation of

- changes in functional connectivity during resting-state, changes in cue-reactivity, and autobiographic memory assessed with fMRI,
- changes in empathy assessed with the Multifaceted Empathy Task,
- changes in personality, self-perception, and patient-therapist relationship assessed with questionnaires, and
- epigenetic mechanisms and in vitro neural profile

before, during, and after psilocybin compared to placebo administration.

Before the start of the study, a pilot study will be conducted to evaluate the feasibility of the fMRI paradigms.

4.4 Safety Objectives

Although this study is not a systematic investigation of safety, this study will also clinically assess safety and tolerability of psilocybin treatment of alcohol dependent patients after physical detoxification. In this study psilocybin will be used as the active treatment. Psilocybin will be given as a single, moderate dose

orally. Psilocybin has been used safely in numerous previous human studies (Tyls et al., 2014). Recent clinical studies using psilocybin have shown that the risks of psilocybin administration are low (Bogenschutz et al., 2015; Griffiths et al., 2016; Ross et al., 2016; Tyls et al., 2014). The following safety endpoints will be used at each visit: occurrence of adverse events, psychological well-being, vital signs, and use of concomitant medication.

5. STUDY OUTCOMES

5.1 Primary Outcome

This study primarily seeks to determine whether psilocybin compared to placebo will reduce alcohol use. The primary endpoint will be alcohol use as measured with the Time-Line Follow-Back (Sobell & Sobell, 1995) at screening, pre-treatment visit, and following psilocybin administration (visits 5, 6, and follow-ups). The primary efficacy value will be alcohol use (average of daily standard units of alcohol use between visit 3 and visit 6) between psilocybin administration and visit 6 (four weeks after psilocybin administration). The Time-Line Follow-Back is a standardized and validated instrument to assess drinking behaviour and is used routinely in clinical trials. Re-instatement of alcohol drinking behaviour > 1 standard drink will be defined as relapse. Relapse will be compared between the placebo and psilocybin group in the time period four weeks after visit 3.

In addition, treatment outcome will also be assessed with the Alcohol Abstinence Self-Efficacy Scale (DiClemente, Carbonari, Montgomery, & Hughes, 1994), and the Penn Alcohol Craving Scale (Flannery, Volpicelli, & Pettinati, 1999) capturing alcohol craving. Both questionnaires will be administered at screening, pre-treatment visit, and following psilocybin administration (visits 5, 6, and follow-ups). The Penn Alcohol Craving Scale will additionally be administered at the treatment visit before substance administration.

5.2 Secondary Outcomes

The effects of psilocybin compared to placebo on the mechanisms underlying changes in alcohol use behaviour is of additional interest in this study. The effects of psilocybin on these secondary endpoints will be measured by the Snaith-Hamilton-Pleasure Scale, the Emotion-Regulation Questionnaire, the Hopelessness Scale, the Frankfurt Self-Concept Scale, the Savoring Believes Inventory, the Temporary Experience of Pleasure Scale. These questionnaires will be administered at baseline (visit 2), and following psilocybin administration (visit 4,5,6 and follow-ups). Furthermore, the Beck Depression Inventory, the Hamilton Anxiety Scale, and the Quality of Life Scale will be administered at screening (visit 1), at baseline (visit 2), and following psilocybin administration (visit 4,5,6 and follow-ups) to assess changes in psychopathology. Mood will be assessed at each visit using the Positive and Negative Affect Schedule. The Stress Questionnaire, the Drinker Inventory of Consequences, and the Symptom Check List will be administered at the screening visit, at visit 6, and at Follow-ups to assess general and alcohol-specific psychopathology. The therapist-patient relationship will be assessed at visits 2,4,5, and 6 using the STEPP/STEPT. The Therapy Questionnaire, capturing the subjective need and effective utilization of therapeutic services will be administered at follow-ups.

a) **The Snaith-Hamilton-Pleasure Scale (SHAPS)** is a 14-item self-report scale evaluating anhedonia, i.e. the inability to experience pleasure or related reduction of ability to react to pleasurable stimuli (Franz et al., 1998).
b) **The Emotion Regulation Questionnaire (ERQ)** is a 10-item self-report scale that differentiates between suppression and reappraisal as main strategies regarding the regulation of emotions.
c) **The Hopelessness Scale (HS-Krampen)** is a 20-item self-report scale evaluating negative expectations about oneself, the environment, and the future life (G. Krampen, 1994).
d) **The Frankfurt Self-Concept Scale (FSCS)** seeks to grasp self-concept changes using items and scales that are sensitive to change as well as graduated answers with 6 alternatives (Deusinger, 1982).
e) **The Savoring Believes Inventory (SBI)** is designed to assess individuals' perceptions of their ability to derive pleasure through anticipating upcoming positive events, savoring positive moments, and reminiscing about past positive experiences were conducted (Bryant, 2003).
f) **The Temporary Experience of Pleasure Scale (TEPS)** is a short 18-item questionnaire, which requires the rating of statements relating to pleasure on a 6-point Likert scale. The scale has a two-factor structure separated into anticipatory and consummatory pleasure (TEPS-ANT and TEPS-CON) (Simon et al., 2018).
g) **Beck Depression Inventory (BDI)** is a validated questionnaire to asses depressive symptoms (Kuhner, Burger, Keller, & Hautzinger, 2007).
h) **The Hamilton Anxiety Scale (HAMA)** is a 14-item clinician-rated scale evaluating anxiety states and changes (Hamilton, 1959).
i) The **Quality of Life Scale (WHOQOL-BREF)** The WHO defines Quality of Life as an individual's perception of their position in life in the context of the culture and value systems in which they live and in relation to their goals, expectations, standards and concerns. It is a broad ranging concept affected

in a complex way by the person's physical health, psychological state, personal beliefs, social relationships and their relationship to salient features of their environment. The WHOQOL-BREF is a validated instrument to assess this concept (Organisation, 2019).

j) **The Positive and Negative Affect Schedule (PANAS)** is a validated questionnaire that consists of two 10-item scales to measure both positive and negative affect. Each item is rated on a 5-point scale of 1 (not at all) to 5 (very much).

k) **The Stress Questionnaire (CQ)** is 4-item self-report scale that measures potential stressors, the burden it elicits and the progress done regarding this issue. It is adapted from the Stress & Coping Inventory (Satow, 2012).

l) **The Drinker Inventory of Consequences (DrInc)** assess the consequences of drinking in five domains (Forcehimes, Tonigan, Miller, Kenna, & Baer, 2007).

m) **The Symptom Checklist** (SCL-90-R) contains 90 items to evaluate a broad range of psychological problems and symptoms of psychopathology within the last seven days. It contains nine syndrome scales: Somatization, Obsession, Insecurity in social contact, Depression, Anxiety, Aggressiveness, Phobic anxiety, Paranoid thinking, and psychoticism (Franke, 1995).

n) **The Stundenbogen (STEPP/STEPT)** are short standardized questionnaires for formative evaluation of psychological counseling. Both questionnaires include 12 items which have to be answered by the patient or client or by the psychotherapist or counselor after a psychotherapy or counseling session. Items are constructed complementarily for therapists and patients and refer to (a) the motivational clarification and development of the patient, (2) the active help for problem-solving by the patient and (3) the therapeutic support experienced by the patient (patient questionnaire) versus that perceived by the therapist (therapist questionnaire) (Günter Krampen & Wald, 2001).

o) **Therapy-Questionnaire (TQ)** is a 9-item self-report scale that aims to capture the subjective need and effective utilization of therapeutic services.

p) Psychological support and counselling sessions at visits 1 – 6 will be tape recorded for qualitative and quantitative analyses of patient reports regarding treatment outcome and quality of the psychological experience on the treatment visit.

q) **Time-Line Follow-Back** will also be used to assess the *Number of Heavy Drinking Days* (HDDs), which was defined as five or more standard units of alcohol in a day for a man and four or more standard units of alcohol in a day for a woman. Furthermore, we evaluate the *Time to Relapse*, which is the amount of self-reported abstinent days post IMP-administration until relapse occurs (relapse is defined as > 1 standard drink).

In addition, the study aims at characterizing the effects of psilocybin compared to placebo on neurocognitive and brain functioning markers of alcohol use disorder at baseline (visit 2), and following psilocybin administration (visit 3 or 4 and visit 6).

a) The **Multifaceted Empathy Task (MET)** is a PC-assisted test that assesses cognitive and emotional aspects related to empathy. It consists of 40 photorealistic stimuli showing people in different emotionally charged situations (20 positive, 20 negative). Each picture is presented 3 times with a different question to assess the 3 different components of empathy. Cognitive empathy is operationalized by the question "What is this person feeling?" and participants have to identify the correct mental state from a list of 4 choices. Explicit emotional empathy is operationalized by the question "How concerned are you for this person" (negative valence pictures) and "How happy are you for this person" (positive valence pictures) with a 9-point Likert scale (1=not at all; 9=very much), respectively. To allow for the measurement of emotional empathy while reducing subjects' tendencies to give socially desirable answers, an implicit emotional empathy condition was also included, which is operationalized by the question "How calm/aroused does this picture make you feel?" with a 9-point Likert scale (1=very calm; 9=very aroused). The MET will be at baseline (visit 2), and following psilocybin administration (visit 4 and visit 6):

b) **Functional Magnetic Resonance Imaging (fMRI)** will be used to measure BOLD signal changes at baseline (visit 2), and following psilocybin administration (visit 3 and 6).

a. **Resting State fMRI.** The resting state is a task free procedure. Participants are instructed to rest without engaging in any specific task or mental activity. Under such "task-free" conditions, it is assumed that BOLD changes are caused by spontaneous and intrinsic neuronal activity within a network of brain regions (Fox & Raichle, 2007). Accumulating evidence indicates that resting-state brain activity is consistently disrupted in addiction disorders (Fede, Grodin, Dean, Diazgranados, & Momenan, 2019) and can be predictive of relapse (Camchong, Stenger, &

Fein, 2013). We have shown that psilocybin alters resting-state connectivity in healthy volunteers (Preller, Duerler, et al., 2019). The measurement will take about 10 minutes.

b. **Cue Reactivity fMRI**. The cue-reactivity task was developed to simulate craving inducing situations in an experimental setting (Jasinska, Stein, Kaiser, Naumer, & Yalachkov, 2014). Altered brain activity due to alcohol stimuli exposure were found in the orbitofrontal cortex, anterior cingulate cortex, the dorsolateral & ventromedial prefrontal cortex, the insula, nucleus accumbens, amygdala, ventral striatum, thalamus and hippocampus (Due, Huettel, Hall, & Rubin, 2002; George et al., 2001; Schneider et al., 2001; Smolka et al., 2006; Vollstadt-Klein et al., 2011; Wrase et al., 2002). Cue-elicited alterations in brain activation are thought to represent a core component of alcohol-induced neuropathology (Heinz, Beck, Grusser, Grace, & Wrase, 2009) and are linked to self-reported craving (Myrick et al., 2004) and the amount of alcohol consumption after relapse (Grusser et al., 2004). Cue-elicited activation has therefore repeatedly been used as an index of treatment efficacy (De Cesarei & Codispoti, 2006; Hermann et al., 2006; Lang, Bradley, & Cuthbert, 1997; Myrick et al., 2008).Therefore, a cue-reactivity task consisting of three visual stimuli types will be conducted: Alcohol cues, positive, and neutral pictures will be presented to participants while they undergo fMRI scanning to induce and assess craving-related brain activity. The paradigm will take about 20 minutes.

Furthermore, pre and post the MRI scan, the participant report their alcohol craving. We want to assess, whether our cue-reactivity task can induce craving.

c. Autobiographical memory fMRI. Current research reported an altered or impaired autobiographical memory retrieval in patients with alcohol use disorders (Nandrino, Gandolphe, & El Haj, 2017). Therefore, we will conduct an autobiographical memory task, designed by Spreng and Grady (2010). This task includes two experimental conditions (remembering; autobiographical memory / theory-of-mind reasoning) and a control condition. Photographs from the International Affective Picture System (Lang et al., 1997) with an associated word will be presented to the participants in the MRI. Followed by the instructions to either remember an event, e.g. “Remember a time when you went out with your family” (remembering), “Imagine what the father in the picture was thinking and feeling” (theory-of-mind reasoning), or “control stimulus”. The patients will retrieve their memory or reasoning within 10 sec and, lastly, rate the clarity of their recollection. The task will last about 30minutes.

d. Diffusion Tensor Imaging (DTI). The DTI is also a task free procedure and is used to map the white matter tractography in the brain. Recent research reported changed fractional anisotropy (FA) in patients with AUD compared to healthy controls (Pandey et al., 2018; Hampton et al. 2019). Pandey et al. (2018) showed that these changes correlated with visuospatial memory and problem-solving ability-related impairments observed in AUD. There are no published studies, about the effect of Psilocybin or lifetime alcohol use on white matter in the brain, yet. The procedure will last for about 12minutes.

c) **Blood samples** will be collected to analyse predictors as well as markers of alcohol use. Blood samples will always be drawn in the morning.

a. **Markers of alcohol use.** In addition to breath samples for alcohol content, Ethylglucuronid, aspartate aminotransferase (AST), alanine aminotransferase (ALT) and gamma-glutamyltransferase (GGT) will be analysed from blood samples (except for Ethylglucuronid which will be analysed in urine samples) collected at the screening visit and visit 6 as objective markers of alcohol use.

b. **Genetic predictors and epigenetic markers.** Genome-wide genetic analyses will be conducted to investigate association between gene variants and treatment outcomes. Genome-wide changes in epigenetic markers of treatment response will be analysed before and after psilocybin administration (visit 2, 4, and 6). Blood samples will be analysed at the following laboratories: PD Dr. Stephanie Witt, Head of the Molecular Genetic Laboratory and Biobank, Dept. of Genetic Epidemiology in Psychiatry, Central Institute of Mental Health, D-68159 Mannheim and Prof. Dr. Markus Nöthen, Life&Brain GmbH, Venusberg-Campus 1, Gebäude 76, D-53127 Bonn.

c. **Induced pluripotent stem cells.** To investigate the *in vitro* neuronal profile of psilocybin in alcohol dependent individuals blood cells before psilocybin administration (visit 2) will be differentiated into cortical neurons. Parameters related to morphology (dendritic branching, quantity and morphology of synapses, synaptic protein content), molecular biology (density of surface 5-HT_{2A} receptors) and network activity (Ca^{2+}-imaging, electrophysiological properties) will be assessed. The collected data will then be correlated to primary treatment outcomes. Blood samples will be analysed at the following laboratory: PD Dr. Stephanie Witt, Head of the

Molecular Genetic Laboratory and Biobank, Dept. of Genetic Epidemiology in Psychiatry, Central Institute of Mental Health, D-68159 Mannheim.

5.3 Other Outcomes of Interest

A routine lab, medical history, ECG, vital signs, and clinical interview will be conducted and assessed at the screening day to ensure mental and physical health. Urine drug and pregnancy tests will be performed at screening and visits 2,3, and 6. Verbal IQ (MWT-B) and personality traits (NEO-FFI) will be assessed at baseline. The Five-Dimension Altered State of Consciousness Questionnaire (5D-ASC) (Dittrich, 1998) will be administered to assess subjective drug effects at the treatment visit. In the pilot study, paradigms described in 5.2 b) will be evaluated for feasibility.

5.4 Safety Outcomes

In this study, the efficacy of psilocybin treatment of alcohol dependent patients after physical detoxification will be systematically assessed. Although this study is not a systematic investigation of safety, this study will also assess safety and tolerability of psilocybin treatment in these patients. The following safety endpoints will be used at each visit: occurrence of adverse events (AEs), psychological and physical well-being, vital signs, and use of concomitant medication. All observed or volunteered abnormal safety endpoints will be recorded in the CRF. The investigator will promptly review documented AEs or other abnormal safety endpoints to determine if

- the abnormal safety endpoint should be classified as an AE,
- if there is a reasonable possibility that the AE was caused by the investigational drug, and
- if the AE meets the criteria for a serious AE (SAE).

The Investigator is responsible for SAE reporting to the CEC.

6. STUDY DESIGN

6.1 General study design and justification of design

2 x 30 patients diagnosed with alcohol use disorder who underwent alcohol withdrawal within the last 6 weeks (30 participants for each study arm) will be investigated in a single-centre, double-blind, placebo-controlled, parallel-group design clinical trial contrasting the acute and persisting effects of psilocybin to those of placebo. Participants will be randomly assigned to psilocybin or placebo group with a 1:1 allocation ration as per a computer generated randomisation schedule using the method of minimization, with age (≤45 years and >45 years), gender, and severity of alcohol use disorder based on the DSM-5 assessment at the screening visit (mild and moderate vs. severe) as binary minimization variables. In the psilocybin treatment arm, participants will receive a single medium dose of psilocybin (25mg). The drug will be administered during an 8 hour sessions in an outpatient setting under close medical and psychological monitoring. The drug administration sessions will occur in the context of an unstructured psychological counselling intervention, with the addition of standardized preparation before and follow-up after the psilocybin administration sessions based on the BRENDA Model developed for the treatment of alcohol use disorders (Starosta et al., 2006). Extensive screening and baseline assessment will be completed, including thorough safety screening and assessment of participant characteristics that could potentially moderate treatment response. Clinical outcomes and changes in several potential mediators of treatment effect, including neurocognition (MET), brain connectivity, cue reactivity, and autobiographic memory (FMRI), and treatment response markers (epigenetic markers, in vitro neuronal profile, alcohol use markers), will be measured at baseline and following treatment. The study procedures comprise a total of 6 visits during a total duration of 6 weeks and includes the following visits: screening visit (5h), pre-treatment visit (5h), treatment visit (8h), post-treatment visits (3, 3 and 4h). The total amount of time needed for the whole study is about 31 hours. Two online surveys after three and six months after substance administration will complement the clinical assessment by providing the opportunity to capture long-term changes in symptomology and the subjective need for therapeutic interventions.

The pilot study (see 7.3) will include a screening session (in case of psilocybin administration: Routine blood analysis, ECG, Structured Clinical Interviews and family history of mental illnesses, drug urine screening, drug pregnancy test (women only), MRI safety questionnaire; no psilocybin administration: MRI safety questionnaire) and a test day including MR scanning. MR scanning will not exceed two hours. The participants will be supervised and supported during the test day in a similar way as patients with alcohol use disorder.

6.2 Methods of minimising bias

The study will be performed in a placebo-controlled, parallel-group randomized (random allocation of treatment arm) and double-blind manner. The pilot study will not be placebo-controlled or randomized since it only serves to evaluate feasibility and data will not be statistically analysed.

6.2.1 Randomisation

A lab member who will have no other role in the study and will not communicate with the patients will randomly allocate the patients to either the psilocybin or the placebo treatment arm with a 1:1 allocation ratio as per a computer generated randomisation schedule using the method of minimization, with age (≤45 years and >45 years), gender, and severity of alcohol use disorder based on the DSM-5 assessment at the screening visit (mild and moderate vs. severe) as binary minimization variables. For the pilot study, participants will not be randomized. Participants will not receive a substance until feasibility of the paradigms without substance administration is confirmed. The next participants will receive psilocybin to ensure feasibility after substance administration.

6.2.2 Blinding procedures

All individual-related data will be encrypted. The subject code will be generated by the investigator at study inclusion and will be individually allocated. Psilocybin and placebo will be administered in gelatine capsules of identical appearance. The blinding will be conducted sequentially, thus preparation of the drug kit for each patient individually upon patient inclusion. A lab member implementing the blinding, will have no other role in the study and will not communicate with the subjects and the investigators about the study and keep a list of the randomization codes, which will allow rapid de-codification in case of emergency.

An emergency code break will be available to the investigators (see 6.3). The pilot study is not blinded.

To determine blinding integrity, the investigators assess and document their own and the patients' assumption about having received psilocybin or placebo at V4. Accuracy of these assumptions will be evaluated after the study has been unblinded.

6.2.3 Other methods of minimising bias

All questionnaires and MR-sequences applied in this study are widely used and well-validated measures.

6.3 Unblinding Procedures (Code break)

In circumstances under which unblinding is permissible, e.g. for SAEs or in case of a medical emergency, an Emergency Code Break in sealed envelopes will be available to the investigators. This Code Break should be opened only in emergency situations when the identity of the investigational product must be known by the investigator in order to provide appropriate medical treatment.

7. STUDY POPULATION

7.1 Eligibility criteria

Participants fulfilling all of the following inclusion criteria are eligible for the study:

- Informed Consent as documented by signature (Appendix Informed Consent Form)
- Male and female in- and outpatients 18 years to 60 years of age
- Right-handedness according to Oldfield (1971) performed during the telephone screening, laterality index ≥ 0.2
- DSM-IV-diagnosis of alcohol use disorder (based on clinical assessment and confirmed by the SCID Interview)
- Having undergone withdrawal treatment from alcohol use or have stopped consuming alcohol within 6 weeks prior to enrolment in the study
- Drug free from any psychotropic and serotonergic medication for at least five days before administration of the study drug or placebo
- No alcohol use between withdrawal treatment and administration of study drug or placebo
- Good physical health with no unstable medical conditions, as determined by medical history, physical examination, routine blood labs, electrocardiogram, urine analysis, and urine toxicology
- Normal level of language comprehension (German or Swiss-German)
- Willing to refrain from drinking caffeinated drinks during the testing days and from consuming psychoactive substances after enrolling in the study until visit 6
- Women of childbearing potential must be using an effective, established method of contraception for the entire study duration, such as oral, injectable, or implantable contraceptives, or intrauterine contraceptive devices. Note: female participants who are surgically sterilised / hysterectomised or post-menopausal for longer than 2 years are not considered as being of child bearing potential.
- Have a family member or friend who can pick them up and stay with them overnight after the psilocybin administration sessions (driving is forbidden at drug treatment days)
- No other medication than reported at study inclusion is allowed until visit 6, except for emergencies

The presence of any one of the following exclusion criteria will lead to exclusion of the participant, for example:

- Allergy, hypersensitivity, or other adverse reaction to previous use of psilocybin or other hallucinogens
- Uncorrected Hypertension (assessed at screening day: higher than 139 systolic and 89 diastolic)
- Women who are pregnant or breast feeding
- Intention to become pregnant during the course of the study,
- Lack of safe contraception, defined as: Female participants of childbearing potential, not using and not willing to continue using a medically reliable method of contraception for the entire study duration, such as oral, injectable, or implantable contraceptives, or intrauterine contraceptive devices, or who are not using any other method considered sufficiently reliable by the investigator in individual cases (Female participants who are surgically sterilised / hysterectomised or post-menopausal for longer than 2 years are not considered as being of child bearing potential)
- Known or suspected non-compliance
- Inability to follow the procedures of the study, e.g. due to language problems, psychological disorders, dementia, etc. of the participant,
- Previous enrolment into the current study
- Enrolment of the investigator, his/her family members, employees and other dependent persons
- Lifetime history of bipolar disorder (I, II, not otherwise specified) based on our clinical interview at screening visit
- Lifetime history of schizophrenia, schizoaffective disorder, or psychosis not otherwise specified based on our clinical interview at screening visit, expect psychotic symptoms under acute alcohol intoxication or during withdrawal treatment
- History of DSM-IV drug dependence other than alcohol (except for caffeine or nicotine) within two months prior to enrolment

- Comorbid Axis I anxiety and depression disorders diagnoses as well as post-traumatic stress disorder and ADHD will be permitted if they do not require treatment after inclusion in the study
- Family history of schizophrenia or schizoaffective disorder, or bipolar disorder type 1 (first or second degree relatives)
- Violent behaviour within last 2 years or history of suicidal behaviour
- Lifetime history of hallucinogen use on more than 10 occasions within last 10 years
- Getting psychotherapeutic or psychological treatment from third parties during the study until visit 6 is forbidden
- Abnormal electrocardiogram
- Any unstable illness as determined by history or laboratory tests
- BMI <17 or >30
- Uncorrected hypo- or hyperthyroidism
- Contraindications to magnetic resonance imaging (MRI safety form)
- During the study, new use or dose changes of already existing concomitant medication without prior informing the investigators is forbidden
- High risk of adverse emotional or behavioural reaction based on investigator's clinical evaluation (e.g., evidence of serious personality disorder, antisocial behaviour, serious current stressors, lack of meaningful social support)
- Participation in another study with investigational drug within the 30 days preceding and during the present study (until visit 6)
- Taking medications that are known to modulate uridine diphosphate glucuronosyltransferase enzyme
- Inhibitors of UGT1A9 and 1A10 should be discontinued at least five half-lives prior to the administration of psilocybin
- Monoamine oxidase and aldehyde or alcohol dehydrogenase inhibitors should be discontinued at least 5 half-lives prior to the dose of psilocybin

7.2 Recruitment and screening

Participants will be recruited from referrals from the Center for Addiction Disorders (ZAE), Zurich, led by PD Dr. Marcus Herdener, other clinical institutions specialized in the treatment of substance use disorders, social media and with study flyers in places open to public. Patients who underwent alcohol withdrawal within the last six weeks and who are interested in participating in the study will be pre-screened using IRB-approved pre-screening forms including basic demographic data and questions related to inclusion and exclusion criteria. Those who pass pre-screening will be invited to a screening visit. Details of the prescreening and screening process are described in section 9.3. Participants for the pilot study will be recruited by word of mouth. Prior to every visit at the Psychiatric University Clinic Zurich, we will phone the patients and ask them about their risk of COVID-19 exposure. This will include infection relevant symptoms, body temperature and contact with people with COVID-19 or people in quarantine. If there is a risk of COVID-19 infection, the visit will be postponed for ten days. These measures reflect the guidelines from the direction of the Psychiatric University Clinic Zurich. They will be updated regularly according to the local situation of the pandemic and will strictly follow the current guidelines of the Bundesamt für Gesundheit (BAG).

7.3 Pilot Study

Prior to the clinical trial involving patients suffering from alcohol use disorder, we will conduct a pilot study to test the administered paradigms and train the investigators. We will recruit up to 15 healthy participants according to the same In- and Exclusion criteria as described in 7.1 except for DSM-IV-diagnosis of alcohol use disorder and withdrawal treatment from alcohol use. If necessary, a subset of these pilot participants will receive psilocybin to test the feasibility of paradigms. Other than ensuring feasibility, the results of the pilot study will not have any impact on the clinical trial involving patients. The data will not be statistically analysed. Participants will be informed about whether they receive psilocybin or not. The pilot study will include a screening session (in case of psilocybin administration: Routine blood analysis, ECG, Structured Clinical Interviews and family history of mental illnesses, drug urine screening, drug pregnancy test (women only), MRI safety questionnaire; no psilocybin administration: MRI safety questionnaire) and a test day including MR scanning. MR scanning will not exceed two hours. The participants will be supervised and supported during the test day in a similar way as patients with alcohol use disorder. Participants in this pilot study will receive separate participant

information and informed consent forms (Appendix 3b).

7.4 Assignment to study groups

Participants will be randomly assigned to psilocybin or placebo group with a 1:1 allocation ration as per a computer generated randomisation schedule using the method of minimization, with age (≤45 years and >45 years), gender, and severity of alcohol use disorder based on the DSM-5 assessment at the screening visit (mild and moderate vs. severe) as binary minimization variables as binary minimization variables. Each participant will be assigned a randomisation code which corresponds to numbers printed on the kit containing the study drug, and which will be matched to a confidential treatment allocation number by a lab member not involved in the study to assign participants either to psilocybin or to placebo. Allocation concealment will be ensured, as the randomisation codes will not be released until the patient has been recruited into the trial, which takes place after all baseline measurements have been completed. The participants, investigators, and sponsor personnel will be masked to treatment allocation. Drug kits will be prepared and labelled with the randomisation code by a lab member not involved in the study. The psilocybin and placebo capsules will be similar in number, colour, shape, size, texture, and taste. To guarantee masking throughout the study, the treatment allocation number will be kept in a confidential key-locked place on-site and will only be accessible by a lab member not involved in the study. In case of a medical emergency the treatment allocation number can be accessed by the investigators (see 6.3). Access to the treatment allocation number will be controlled and documented.

7.5 Criteria for withdrawal / discontinuation of participants

The study may be discontinued by the participant at any time without disclosure of reasons. The study may also be discontinued by the investigator if the participant does not comply with the study-specific agreements, if further study participation would bear a risk to the health of the participant, or in case of pregnancy. If the study is discontinued, drop-out date and reasons must be documented in the case report form (CRF). Participants who discontinue the study will be replaced until the determined sample size of participants completing the whole study is reached. Please see section 9.2.5 for description of follow-up procedures.

8. STUDY INTERVENTION

8.1 Identity of Investigational Products

Participants will be randomly allocated to one of two study arms and will receive either a single dose of active drug (psilocybin) or placebo (100% mannitol) at visit no. 3 (see section 9.1). No other treatments will be administered during the study. Psilocybin and placebo will be administered orally as white, oval-shaped, smooth, tasteless gelatine capsules. The psilocybin and placebo capsules will be similar in number, colour, shape, size, texture, and taste. Psilocybin will be administered as a single dose of 25mg. Drug preparation and labelling of drug kits will be done by a lab member not involved in the study.

8.1.1 Experimental Intervention

Pharmaceutically pure psilocybin will be used. Psilocybin will be provided by Usona Institute, 2800 Woods Hollow Rd., Madison, WI 5371, USA, with the authorization of the Federal Office of Public Health (BAG), Bern. The investigator will pay for the product (no contract). The drug was synthesized according to Good Manufacturing Practice.

8.1.2 Control Intervention

Placebo (100% mannitol) will be obtained from Dr. Cédric Hysek, Apotheke Dr. Hysek AG, Bözingenstrasse 162, 2500 Biel, Switzerland.

8.1.3 Packaging, Labelling and Supply (re-supply)

All capsules will be of identical appearance. The capsules will be taken in charge of a lab member not involved in the study who will prepare and label the participant-specific drug kits. Drug kits will contain one capsule and be labelled according to Good Manufacturing Practice (EUDRALEX Volume 4 – Medicinal Products for Human and Veterinary Use). Labels will be attached to the drug kit (dark tight-closed plastic bags) and will contain the following information:

Psychiatrische Universitätsklinik Zürich

PHASE II, RANDOMIZED, DOUBLE BLIND, PLACEBO CONTROLLED, PARALLEL GROUP, SINGLE CENTER STUDY OF PSILOCYBIN EFFICACY AND MECHANISM IN ALCOHOL USE DISORDER– PSIALC-133

Psilocybin 25mg oder Placebo Kapsel

Probanden-Nr.: XXX   Randomisierungs-Nr. XXXX

Dosierung: 25mg einmalig oral einnehmen

Chargennummer:XXXXXX   Verfallsdatum: XX.XX.XXXX

Für Kinder unzugänglich aufbewahren

Bei 16° bis 24° C fest verschlossen lichtgeschützt lagern

Nur für klinischen Versuch

Sponsor: Prof. Dr. med. Franz X. Vollenweider, Psychiatrische Universitätsklinik Zürich, Lenggstrasse 31, 8032 Zürich, Tel.: +41 (0)58 384 2604 (direkt) oder +41 (0)58 384 2404 (Sekretariat)

Upon receipt of the test drug, an inventory will be performed and a drug receipt log filled out and signed

by the person accepting the shipment. It is important that the designated study staff counts and verifies that the shipment contains all the items noted in the shipment inventory. Any damaged or unusable study drug in a given shipment (active drug or comparator) will be documented in the study files. Active or comparator drugs will be assigned to each participant by a lab member not involved in the study according to treatment allocation (see 7.4). To implement masking, drug kits will be prepared and labelled by this lab member who then forwards the drug kits to the investigator. The investigator will then dispense the study drug to the participants. Regular study drug reconciliation will be performed to document drug assigned; drug consumed, and drug remaining. This reconciliation will be logged on the drug accountability form, and signed and dated by the study team. Both empty drug kits (plastic bags with label) and unused study drug will be returned to the lab member responsible for drug preparation. This will also be logged on the drug accountability form.

8.1.4 Storage Conditions

Both psilocybin and placebo will be stored in tightly sealed plastic bottles and kept in a locked device at 16-24°C in the Medical Centre Pharmacy of the Psychiatric Hospital Zurich with restricted access onsite. The temperature will be regularly controlled by the Medical Centre Pharmacy.

8.2 Administration of experimental and control interventions

8.2.1 Experimental Intervention

In the experimental treatment arm, participants will receive a single dose of psilocybin at visit no. 3 (see section 9.1). No other treatments or diagnostic agents will be administered during the study. Psilocybin will be administered orally as white, oval-shaped, smooth, tasteless gelatine capsules. Psilocybin dose will be 25 mg. This dose is based on a previous study showing that doses between 20 and 30 mg of psilocybin significantly reduced drinking behaviour in alcohol addicted patients, with large pre–post effect sizes, as well as produced significant changes in psychological measures relevant to drinking. Importantly, no significant treatment-related adverse events were recorded (Bogenschutz et al., 2015).

Participants exhibited significant improvement in drinking, with large pre–post effect sizes, as well as significant changes in psychological measures relevant to drinking after the administration of psilocybin (0.3 mg/kg and 0.4mg/kg). No significant treatment-related adverse events were recorded (Bogenschutz et al., 2015). In all previous studies investigating the effects of psilocybin in healthy and clinical populations moderate and even high doses of psilocybin were well-tolerated, and none of the volunteers reported persisting adverse effects (Bogenschutz et al., 2015; Carhart-Harris et al., 2016; M. W. Johnson et al., 2014; Preller et al., 2016; Ross et al., 2016; Studerus, Gamma, Kometer, & Vollenweider, 2012; Studerus et al., 2011).

8.2.2 Control Intervention

In the control treatment arm, participants will receive a single dose of placebo at visit no. 3 (see section 9.1). No other treatments or diagnostic agents will be administered during the study. Placebo will be administered orally as white, oval-shaped, smooth, tasteless gelatine capsules.

8.3 Dose / Device modifications

This study comprises a onetime application of a single dose. Therefore, no dose modifications are planned.

8.4 Compliance with study intervention

This study comprises a onetime application of a single dose of psilocybin, which will be directly dispensed to the participants by the investigators at visit no. 3 (see section 9.1). No other treatments or diagnostic agents will be administered during the study. Therefore, there is no need to monitor compliance with treatment (psilocybin). To exclude current use of psychoactive drugs a urine test will be done at the beginning of each test session. Furthermore, a urine pregnancy test will be done for all women capable of bearing children.

8.5 Data Collection and Follow-up for withdrawn participants

For whatever reason a participant or an investigator decides that a participant should withdraw from the study, the investigator will make efforts to conduct an exit visit to assess the safety and well-being of the participant. The exit visit will include vital signs, safety assessments, and reasons for withdrawal. The exit visit will be performed as soon as possible after the withdrawal. If a participant does not agree to an exit visit, a telephone interview will be performed instead, and the participant will be questioned on physical well-being, safety issues, and reasons for withdrawal. When a participant withdraws from the study, the data collected on the participant to the point of withdrawal remains part of the study database and will not be removed.

8.6 Trial specific preventive measures

Risks are minimized by a) careful selection of participants, b) extensive preparation, c) presence of qualified study personnel during the psilocybin sessions, d) a safe and reassuring physical environment, and e-g) follow-up for possible residual adverse effects. These preventive measures are incorporated into our protocol as follows:

a) Participant selection: Exclusion criteria are designed to exclude patients who would be at elevated risk for adverse events due to psilocybin. These criteria are listed in Section 7.1., and include both medical exclusions and psychiatric exclusions for serious psychopathology, history of violent or suicidal behaviour, and family history of psychosis.

b) Participant preparation: In addition to the information and discussion provided in the informed consent process during the initial screening visit, participants' will be psychologically prepared by one of the investigators. Preparation will include 1) open-ended questions to establish rapport, learn about the participant's history, belief system, and values, assess motivation and expectations for the study, and discuss any previous experience with hallucinogens; 2) detailed information about the physiological and psychological effects of psilocybin; 3) emphasis that the purpose of the psilocybin sessions is to help overcome alcohol use disorder symptomatology via psilocybin's pharmacological and psychological effects, and discussion of how this could work; 4) advice as to how to deal with dysphoric reactions to psilocybin, should they occur; 5) discussion of ground rules for the session, including adherence to the protocol (compliance with dietary restrictions, e.g. alcohol, caffeinated drinks, and other psychoactive substances; effective contraception; restricted use of concomitant medication; transport and care by significant others after psilocybin session); 6) .psychosocial counselling based on the BRENDA program (Starosta et al., 2006).

c) One of the investigators will serve as the monitor who will attend and interact with the participant during the psilocybin session. All investigators will be trained by the PI and the sponsor. A physician will always be on site in case of emergencies. After the treatment visit, participants will only be released after having been examined by a physician. Patients will be picked up by a family member or friend who can stay with them over night after the treatment visit. Details on the release procedures are documented in an SOP.

d) The psilocybin session will take place on-site in an environment that is appropriate for hallucinogen administration sessions. To the extent possible, the room used for the session will be specially prepared for the session to provide a warm, quiet and home-like rather than a stark clinical quality because of the large influence that setting can have on the subjective effects of hallucinogens. Patients will be allowed to lie on a couch or to move freely. Interaction with the patients will be supportive and non-directional. To establish a quiet, relaxing, introspective ambience, no cell phones, laptops, or other electronic devices will be allowed during the session.

e) During treatment sessions, study personnel will be responsible for monitoring participants for possible clinical deterioration or other problems, and for implementing appropriate courses of action.

f) Post-treatment visits will be conducted one day, 14 days, and 28 days after substance administration. During these visits, participants will receive psychosocial counselling based on the BRENDA method specifically designed for the treatment of alcohol use disorder (Starosta et al., 2006). Furthermore, they will have time to discuss the treatment visit with the investigator. Changes in symptomatology or need for therapeutic interventions will be monitored.

g) An online survey three and six months after the substance administration will ensure monitoring of subtle changes in symptomology and the subjective need for therapeutic interventions over a longer time-course.

8.7 Concomitant Interventions (treatments)

Concomitant interventions will be assessed at study entry and during the study in order to prevent confound or undesirable impact on patients and study endpoint measures, respectively. In general, concomitant interventions, either before or during the study, are forbidden. In particular, the following drugs or procedures are forbidden:

a) Psychotropic medication for at least five days before administration of the study drug or placebo

b) New use or dose changes of already existing concomitant medication without prior information of the investigators during the study

c) Drinking alcohol before visit 3, or drinking alcohol and caffeinated drinks during testing days

d) Getting psychotherapeutic or psychological treatment from third parties during the study (up to visit 6)

e) Participation in another study with investigational drug or psychological intervention within the 30 days preceding and during the present study

f) Any other drugs or procedures which may influence study performance or endpoint measures.

g) Taking medications that are known to modulate uridine diphosphate glucuronosyltransferase enzyme

h) Inhibitors of UGT1A9 and 1A10 should be discontinued at least five half-lives prior to the administration of psilocybin

i) Monoamine oxidase and aldehyde or alcohol dehydrogenase inhibitors should be discontinued at least 5 half-lives prior to the dose of psilocybin

The following drugs or procedures are allowed, but must be discussed with or communicated to the study personnel:

a) Medical drugs or interventions unrelated to alcohol use or other psychiatric problems before or during the study

b) Rescue drugs during the study. Acute adverse reactions to psilocybin initially will be managed by increasing psychological support (“talking down”). Rescue medication will be available for onetime administration if needed to treat: 1) hypertension (Adalat retard® 20mg oral); 2) anxiety (Temesta® 1-3 mg oral); or 3) acute psychosis posing a danger to the participant or others (Zyprexa® 5-10 mg oral). Referral for emergency treatment and/or hospitalization will be available on site. A study clinician will be available by pager or phone at all times during study participation. Use of these mediations is expected to occur uncommonly if at all in this study.

All concomitant and/or rescue treatment(s) have to be recorded in the CRF.

8.8 Study Drug / Medical Device Accountability

Active or comparator drugs, which will be provided to the Sponsor, will be kept in a secure, limited access storage area under the recommended storage conditions (see Section 8.1.4). Regular study drug reconciliation will be performed to document receipt of test drug; drug assigned; drug consumed; drug remaining; and drug destruction. Drug accountability will be accurately and adequately recorded, including dates, lot number, quantities received/ returned/ missing/ destructed (see Section 8.1.3).

8.9 Return or Destruction of Study Drug

At the completion of the study, there will be a final reconciliation of drug shipped, drug consumed, and drug remaining. This reconciliation will be logged on the drug accountability form, signed and dated. Any discrepancies noted will be investigated, resolved, and documented prior to destruction of unused study drug. Drug destroyed on site will be documented in the study files.

9. STUDY ASSESSMENTS

9.1 Study flow chart / table of study procedures and assessments

Study flow chart

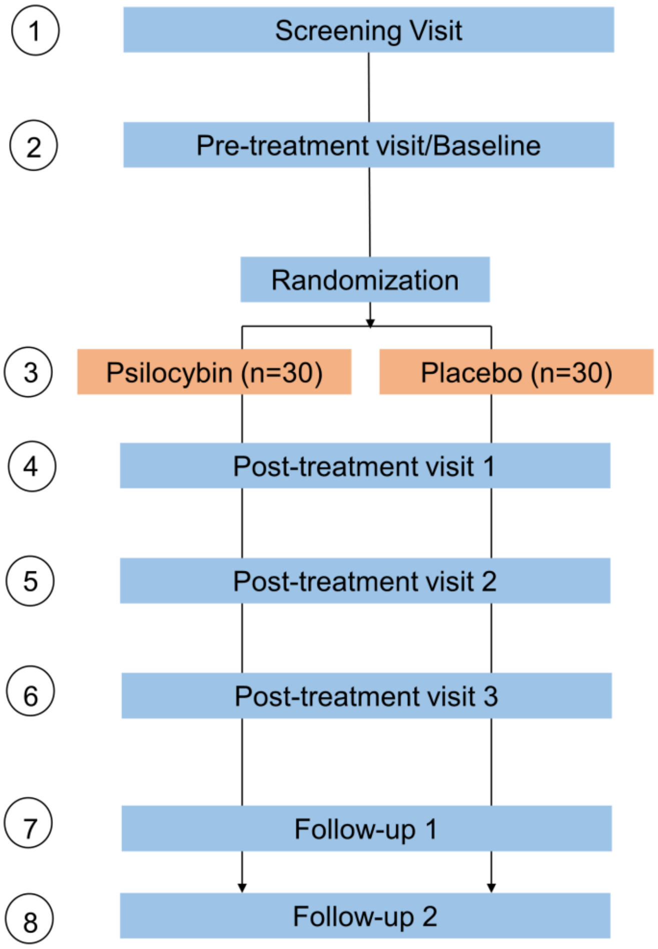

Table of study procedures and assessments

Study Periods	Screening/Pre-treatment Visits		Treatment Period	Post-treatment Visits			Follow-up surveys	
Visit	1	2	3	4	5	6	7	8
Time	t0-14d +/-5d	t0-5d +/-3d	t0	t0+1d	t0+14d +/- 5d	t0+28d +/- 3d	t0+90d +/- 10d	t0+180d +/- 15d
Patient Information and Informed Consent	x							
Demographics, Medical/Psychiatric History, including Family History	x							
Breath Sample for Blood Alcohol Content	x	x	x	x	x	x		
Time-Line Follow Back	x	x	x		x	x	x	x

Alcohol Self-Efficacy Scale	x	x		x	x	x	x	x
Penn Alcohol Craving Scale	x	x	x	x	x	x	x	x
Quality of Life Scale	x	x		x	x	x	x	x
Drinker Inventory of Consequences	x					x	x	x
Beck Depression Inventory	x	x		x	x	x	x	x
Hamilton Anxiety Scale	x	x		x	x	x	x	x
Stress Questionnaire	x					x	x	x
Symptom Checklist	x			x	x	x	x	x
Positive and Negative Mood Schedule	x	x	x	x	x	x	x	x
Structured Clinical Interview (SCID I and II)	x							
Diagnosis of Alcohol Use Disorder Severity based on DSM-5	x							
Verbal IQ (MWT-B)	x							
NEO-FFI	x						x	x
In- /Exclusion Criteria	x							
MRI Safety Form	x	x	x			x		
Physical Examination	x							
Echocardiogram	x							
Vital Signs	x	x	x	x	x	x		
Body Weight	x							
Concomitant Medication Form	x	x	x	x	x	x	x	x
Drug Use Questionnaire	x	x	x	x	x	x	x	x
Drug Urine Test	x	x	x			x		
Routine Laboratory Tests[1]	x							
Laboratory Tests for Alcohol Use Markers[2]	x					x		
Urine Pregnancy Test[3] (Women only)	x	x	x			x		
Randomization		x						
Study Medication Administration			x					
fMRI Resting-State		x	x			x		
fMRI Cue-Reactivity		x	x			x		
fMRI Autobiographic Memory		x	x			x		
Multifaceted Empathy Test		x		x		x		
Altered States of Consciousness Questionnaire			x					
Psychological Support (tape recorded) and BRENDA program		x	x	x	x	x		

Stundenbogen (STEPP, STEPT)		x		x	x	x		
(Epi)Genetics		x		x		x		
Induced pluripotent stem cell		x						
Frankfurt Self-Concept Scale		x		x	x	x	x	x
Emotion-Regulation Questionnaire		x		x	x	x	x	x
Snaith-Hamilton-Pleasure Scale		x		x	x	x	x	x
Savoring Beliefs Inventory		x		x	x	x	x	x
Temporary Experience of Pleasure		x		x	x	x	x	x
Hopelessness Scale		x		x	x	x	x	x
Adverse Events	x	x	x	x	x	x		
Therapy Questionnaire		x					x	x

[1]Hemoglobin, white blood cell count (WBC), Red blood cell count (MCV, MCH), Blood clotting (INR), sodium (Na), potassium (K), calcium (Ca), glucose, creatinine, thyroid stimulating hormone (TSH)

[2]Ethylglucuronid, aspartate aminotransferase (AST), alanine aminotransferase (ALT), gamma-glutamyltransferase (GGT)

[3]human corionic gonadotropin (hCG)

9.2 Assessments of outcomes

9.2.1 Assessment of primary outcome

The primary endpoint of the study is will be alcohol use behaviour as measured with the Time-Line Follow-Back (Sobell & Sobell, 1995) at screening, pre-treatment visit, and following psilocybin administration (visits 5, 6, and follow-ups). The Time-Line Follow-Back will be filled in by the patients every day from screening to second follow up. New Time-Line Follow-Back calendars will be handed out to the participants at screening, pre-treatment visit, and following psilocybin administration (visits 5, 6) to avoid loss of data. At visit 6 patients, will receive Time-Line Follow-Back calendars for the following six months together with six standardised and stamped envelopes. Participants will send the calendar using these envelopes to “Nathalie Rieser, Psychiatrische Universitätsklinik Zürich, Lenggstr. 31, 8032 Zürich” once a month. The participant ID will be written on the calendar. No other personal information will be written on the calendar or the envelope. Alternatively, participants can send a photograph or scan of their Time-Line Follow-Back to our study mail (studie133@bli.uzh.ch) once a month. The Time-Line Follow-Back is a standardized and validated instrument to assess drinking behaviour and is used routinely in clinical trials. In addition, treatment outcome will also be assessed with the Alcohol Abstinence Self-Efficacy Scale (DiClemente et al., 1994), and the Penn Alcohol Craving Scale (Flannery et al., 1999) capturing alcohol craving. Both questionnaires will be administered at screening, pre-treatment visit, and following psilocybin administration (visits 5, 6, and follow-ups). The Penn Alcohol Craving Scale will additionally be administered at the treatment visit before substance administration.

9.2.2 Assessment of secondary outcomes

The effects of psilocybin compared to placebo on the mechanisms underlying changes in alcohol use behaviour is of additional interest in this study. Secondary endpoints include: The Snaith-Hamilton-Pleasure Scale, the Emotion-Regulation Questionnaire, and the Hopelessness Scale, the Frankfurt Self-Concept Scale, the Savoring Believes Inventory, the Temporary Experience of Pleasure Scale, and will be measured at baseline (visit 2), and following psilocybin administration (visit 4,5,6 and follow-ups). Furthermore, the Beck Depression Inventory, the Hamilton Anxiety Scale, and the Quality of Life Scale will be administered at screening (visit 1), at baseline (visit 2), and following psilocybin administration (visit 4,5,6 and follow-ups). Mood will be assessed at each visit using the Positive and Negative Affect

Schedule. The Stress Questionnaire, the Drinker Inventory of Consequences, and the Symptom Check List will be administered at the screening visit, at visit 6, and at Follow-ups to assess general and alcohol-specific psychopathology. The therapist-patient relationship will be assessed at visits 2,4,5, and 6 using the STEPP/STEPT. Psychological support and counselling sessions at visits 1 – 6 will be tape recorded for qualitative and quantitative analyses of patient reports regarding treatment outcome and quality of the psychological experience on the treatment visit. To report alcohol drinking behaviour in more detail, we use the Time-Line Follow-Back to assess *Number of Heavy Drinking Days* (HDDs, five or more standard units of alcohol in a day for a man and four or more standard units of alcohol in a day for a woman) and *Time to Relapse* post IMP-administration (relapse is defined as > 1 standard drink). In addition, the Multifaceted Empathy Test (MET) is being used to assess the effects of psilocybin compared to placebo on changes in empathy. The MET is a highly validated and standardized computer software and will be performed by the patients in a quiet testing room with access to a computer at baseline (visit 2), and following psilocybin administration (visit 4 and 6). Furthermore, functional Magnetic Resonance Imaging (fMRI) is being used to assess the effects of psilocybin compared to placebo on brain functioning markers of alcohol addiction. BOLD-signal changes at rest and in response to the paradigms described in 5.2 will be compared between psilocybin and placebo condition. FMRI measurement will be performed at baseline (visit 2), and directly after psilocybin administration (visit 3) and four weeks after psilocybin administration (visit 6). All FMRI measurements will be performed using the Philips Achieva 3T MR-System equipped with a 32-channel receive head coil and MultiTransmit parallel RF transmission located at the MRI center of the Department of Psychiatry, Psychotherapy and Psychosomatics, University Hospital for Psychiatry. Participants will be instructed to lie as still as possible in the scanner. FMRI data are acquired using a whole brain gradient-echo EPI sequence (TR=2500ms, TE=35ms, slice thickness 3mm, 40 axial slices, no slice gap, field of view 240x240mm2, in-plane resolution 3×3mm, SENSE reduction factor 2.0). Additionally, high-resolution anatomical images (voxel size=1×1×1mm) are acquired using a standard T1-weighted 3-D MP-RAGE sequence. Finally, the effects of psilocybin compared to placebo on genetic and epigenetic markers, will be assessed at baseline (visit 2), and post treatment (visit 4 and 6). Induced pluripotent stem cells will be investigated from blood samples at visit 2. Blood samples will always be drawn in the morning. Blood samples will be analysed at the following laboratories: PD Dr. Stephanie Witt, Head of the Molecular Genetic Laboratory and Biobank, Dept. of Genetic Epidemiology in Psychiatry, Central Institute of Mental Health, D-68159 Mannheim and Prof. Dr. Markus Nöthen, Life&Brain GmbH, Venusberg-Campus 1, Gebäude 76, D-53127 Bonn. Breath samples for blood alcohol content will be collected at every visit. Ethylglucuronid, aspartate aminotransferase (AST), alanine aminotransferase (ALT) and gamma-glutamyltransferase (GGT) will be analysed from blood samples (except for Ethylglucuronid which will be analysed from urine sample) collected at the screening visit and visit 6 as objective markers of alcohol use.

9.2.3 Assessment of other outcomes of interest

A routine lab, medical history, ECG, vital signs, and clinical interview will be conducted and assessed at the screening day to ensure mental and physical health. Urine drug and pregnancy tests will be performed at screening and visits 2,3, and 6. Verbal IQ (MWT-B) and personality traits (NEO-FFI) will be assessed at baseline. The Five-Dimension Altered State of Consciousness Questionnaire (5D-ASC) (Dittrich, 1998) will be administered to assess subjective drug effects at the treatment visit.

9.2.4 Assessment of safety outcomes

9.2.4.1 Adverse events

In this study, the efficacy of psilocybin on relapse behaviour in alcohol dependent patients will be systematically assessed. Although this study is not a systematic investigation of safety, this study will also assess safety and tolerability of psilocybin treatment in patients with alcohol use disorder. The following safety endpoints will be used at each visit: occurrence of adverse events (AEs), psychological and physical well-being, vital signs, and use of concomitant medication. Clinical study participants will be routinely questioned about AEs at study visits. The well-being of the participants will be ascertained by neutral questioning ("How are you?"). All observed or volunteered abnormal safety endpoints will be recorded in the CRF. The investigator will promptly review documented AEs or other abnormal safety endpoints to determine if

• the abnormal safety endpoint should be classified as an AE,

• if there is a reasonable possibility that the AE was caused by the investigational drug, and

• if the AE meets the criteria for a serious AE (SAE).

The Investigator is responsible for SAE reporting to the CEC (see Section 10 for AE definition and procedures).

9.2.4.2 Laboratory parameters

A routine lab will be conducted at the screening day to ensure physical health. Urine drug and pregnancy tests will be performed at screening and visits 2, 3, and 6.

9.2.4.3 Vital signs

Heart beat and blood pressure will be measured at each visit in supine position after 5 minutes resting. During drug treatment session, vital signs will be measured before and during treatment sessions in 30-60 minutes’ intervals.

9.2.5 Assessments in participants who prematurely stop the study

For whatever reason a participant or an investigator decides that a participant should withdraw from the study, the investigator will make efforts to conduct an exit visit to assess the safety and well-being of the participant. The exit visit will include vital signs, safety assessments, and reasons for withdrawal. The exit visit will be performed as soon as possible after the withdrawal. If a participant does not agree to an exit visit, a telephone interview will be performed instead, and the participant will be questioned on physical well-being, safety issues, and reasons for withdrawal. When a participant withdraws from the study, the data collected on the participant to the point of withdrawal remains part of the study database and will not be removed.

9.3 Procedures at each visit

9.3.1 Pre-Screening (Phone Call)

A telephone screening will be performed by an investigator or a trained study nurse prior to the first visit for initial screening and information purposes. The following procedures will be performed:

- Phone Screening Questionnaire

Data of participants who are not invited to the Screening Visit will be destroyed.

9.3.2 Visit 1/Screening Visit

Visit 1 will be scheduled 14 days (+/- 5 days) before the treatment visit and will last about 5 hours. The following exams/tests will be performed:

- Patient Information and Informed Consent
- Demographics, Medical/Psychiatric History, including Family History
- Breath Sample for Blood Alcohol Content
- Time-Line Follow Back
- Questionnaires: Alcohol Self-Efficacy Scale, Penn Alcohol Craving Scale, Quality of Life Scale, Drinker Inventory of Consequences, Beck Depression Inventory, Hamilton Anxiety Scale, Stress Questionnaire, Symptom Checklist, Positive and Negative Mood Schedule, Verbal IQ (MWT-B), NEO-FFI, MRI Safety Form, Concomitant Medication Form, Drug Use Questionnaire
- Interviews: Structured Clinical Interview (SCID I and II), Diagnosis of Alcohol Use Disorder Severity based on DSM-5
- Physical Examination: Echocardiogram, Vital Signs, Body Weight, Drug Urine Test, Routine Blood Test, Laboratory Tests for Alcohol Use Markers,Urine Pregnancy Test (women only)
- Confirmation of In-and Exclusion Criteria

9.3.3 Visit 2/Pre-treatment and Baseline Visit

Visit 2 will be scheduled 5 days (+/- 3 days) before the treatment visit and will last about 5 hours. The

following exams/tests will be performed:

- Breath Sample for Blood Alcohol Content
- Time-Line Follow Back
- Questionnaires: Alcohol Self-Efficacy Scale, Penn Alcohol Craving Scale, Quality of Life Scale, Beck Depression Inventory, Hamilton Anxiety Scale, Positive and Negative Mood Schedule, MRI Safety Form, Concomitant Medication Form, Drug Use Questionnaire, Stundenbogen (STEPP), Frankfurt Self-Concept Scale, Emotion-Regulation Questionnaire, Snaith-Hamilton-Pleasure Scale, Savoring Beliefs Inventory, Temporary Experience of Pleasure, Hopelessness Scale
- Vital Signs
- Drug Urine Test
- Urine Pregnancy Test (Women only)
- FMRI: Resting-State, Cue-Reactivity, Autobiographic Memory, DTI
- Multifaceted Empathy Test
- Psychological Support (tape recorded) and BRENDA program
- Clinician-administered ratings: Stundenbogen (STEPT)
- Epigenetic Measures and pluripotent stemcells

9.3.4 Visit 3/Treatment Visit

Visit 3 will be the treatment visit. It will last about 8 hours. The following exams/tests will be performed:

- Breath Sample for Blood Alcohol Content
- Time-Line Follow Back
- Penn Alcohol Craving Scale
- MRI Safety Form
- Concomitant Medication Form
- Drug Use Questionnaire
- Drug Urine Test
- Urine Pregnancy Test3 (Women only)
- Positive and Negative Mood Schedule (before administration of study medication and 6h after administration of study medication)
- Administration of study medication (between 9:00 and 9:30 am)
- Vital signs (every 30-60 minutes)
- FMRI: Resting-State, Cue-Reactivity, Autobiographic Memory (starting 1 hour after administration of study medication)
- Altered States of Consciousness Questionnaire (6 hours after administration of study medication, short version: 2.5 hours after administration of study medication)
- Psychological Support (tape recorded) and BRENDA program

9.3.5 Visit 4/Post-treatment visit

Visit 4 will be scheduled 1 day after the treatment visit and will last about 3 hours. The following exams/tests will be performed:

- Breath Sample for Blood Alcohol Content
- Questionnaires: Alcohol Self-Efficiency Scale, Penn Alcohol Craving Scale, Quality of Life Scale, Beck Depression Inventory, Hamilton Anxiety Scale, Symptom Checklist, Positive and Negative Mood Schedule, Concomitant Medication Form, Drug Use Questionnaire, Frankfurt Self-Concept Scale, Emotion-Regulation Questionnaire, Snaith-Hamilton-Pleasure Scale, Savoring Beliefs Inventory, Temporary Experience of Pleasure, Hopelessness Scale, Stundenbogen (STEPP)
- Vital Signs
- Multifaceted Empathy Test
- Psychological Support (tape recorded) and BRENDA program
- Clinician-administered ratings: Stundenbogen (STEPT)
- Epigenetic Measures

9.3.6 Visit 5/Post-treatment visit

Visit 5 will be scheduled 14 days (+/- 5 days) after the treatment visit and will last about 3 hours. The following exams/tests will be performed:

- Breath Sample for Blood Alcohol Content
- Time-Line Follow Back
- Questionnaires: Alcohol Self-Efficacy Scale, Penn Alcohol Craving Scale, Quality of Life Scale, Beck Depression Inventory, Hamilton Anxiety Scale, Symptom Checklist, Positive and Negative Mood Schedule, Concomitant Medication Form, Drug Use Questionnaire, Frankfurt Self-Concept Scale, Emotion-Regulation Questionnaire, Snaith-Hamilton-Pleasure Scale, Savoring Beliefs Inventory, Temporary Experience of Pleasure, Hopelessness Scale, Stundenbogen (STEPP)
- Vital Signs
- Psychological Support (tape recorded) and BRENDA program
- Clinician-administered ratings: Stundenbogen (STEPT)

9.3.7 Visit 6/Post-treatment visit

Visit 6 will be scheduled 28 days (+/- 3 days) after the treatment visit and will last about 4 hours. The following exams/tests will be performed:

- Breath Sample for Blood Alcohol Content
- Time-Line Follow Back
- Questionnaires: Alcohol Self-Efficacy Scale, Penn Alcohol Craving Scale, Quality of Life Scale, Drinker Inventory of Consequences, Beck Depression Inventory, Hamilton Anxiety Scale, Stress Questionnaire, Symptom Checklist , Positive and Negative Mood Schedule, MRI Safety Form, Frankfurt Self-Concept Scale, Emotion-Regulation Questionnaire, Snaith-Hamilton-Pleasure Scale, Savoring Beliefs Inventory , Temporary Experience of Pleasure, Hopelessness Scale, Concomitant Medication Form, Drug Use Questionnaire, Stundenbogen (STEPP)
- Vital Signs
- Drug Urine Test
- Blood Analyses: Laboratory Tests for Alcohol Use Markers, Epigenetic Measures
- Urine Pregnancy Test (Women only)
- fMRI: fMRI Resting-State, fMRI Cue-Reactivity, fMRI Autobiographic Memory, DTI
- Multifaceted Empathy Test
- Psychological Support (tape recorded) and BRENDA program
- Clinician-administered ratings: Stundenbogen (STEPT)

9.3.8 Visit 7/Follow-up (Survey)

The first follow-up measurement will be conducted three months (+/- 10 days) after treatment in an online-survey format and consists of the following questionnaires:

- Time-Line Follow Back
- Alcohol Self-Efficacy Scale
- Penn Alcohol Craving Scale
- Quality of Life Scale
- Drinker Inventory of Consequences
- Beck Depression Inventory
- Hamilton Anxiety Scale
- Stress Questionnaire
- Symptom Checklist
- Positive and Negative Mood Schedule
- NEO-FFI
- Concomitant Medication Form
- Drug Use Questionnaire
- Frankfurt Self-Concept Scale
- Emotion-Regulation Questionnaire

- Snaith-Hamilton-Pleasure Scale
- Savoring Beliefs Inventory
- Temporary Experience of Pleasure
- Hopelessness Scale
- Therapy Questionnaire

9.3.9 Visit 8/Follow-up (Survey)

The first follow-up measurement will be conducted six months (+/- 15 days) after treatment in an online-survey format and consists of the following questionnaires:

- Time-Line Follow Back
- Alcohol Self-Efficacy Scale
- Penn Alcohol Craving Scale
- Quality of Life Scale
- Drinker Inventory of Consequences
- Beck Depression Inventory
- Hamilton Anxiety Scale
- Stress Questionnaire
- Symptom Checklist
- Positive and Negative Mood Schedule
- NEO-FFI
- Concomitant Medication Form
- Drug Use Questionnaire
- Frankfurt Self-Concept Scale
- Emotion-Regulation Questionnaire
- Snaith-Hamilton-Pleasure Scale
- Savoring Beliefs Inventory
- Temporary Experience of Pleasure
- Hopelessness Scale
- Therapy Questionnaire

10. SAFETY

The Sponsor's SOPs provide more detail on safety reporting.

During the entire duration of the study, all adverse events (AE) and all serious adverse events (SAEs) are collected, fully investigated and documented in source documents and case report forms (CRF). Study duration encompassed the time from when the participant signs the informed consent until the last protocol-specific procedure has been completed, including a safety follow-up period.

10.1. Definition and assessment of (serious) adverse events and other safety related events

An **Adverse Event (AE)** is any untoward medical occurrence in a patient or a clinical investigation participant administered a pharmaceutical product and which does not necessarily have a causal relationship with the study procedure. An AE can therefore be any unfavourable and unintended sign (including an abnormal laboratory finding), symptom, or disease temporally associated with the use of a medicinal (investigational) product, whether or not related to the medicinal (investigational) product. [ICH E6 1.2] Since this study is invetigating a patient population suffering from alcohol use disorder, increased AST, ALT, and GCT levels are expected. AST, ALT, and GCP levels < 100 will therefore not be considered AEs.

A **Serious Adverse Event (SAE)** is classified as any untoward medical occurrence that:

- results in death,
- is life-threatening,
- requires in-patient hospitalization or prolongation of existing hospitalisation,
- results in persistent or significant disability/incapacity, or
- is a congenital anomaly/birth defect.

SAEs should be followed until resolution or stabilisation. Participants with ongoing SAEs at study termination (including safety visit) will be further followed up until recovery or until stabilisation of the disease after termination.

Assessment of Causality

Both Investigator and Sponsor make a causality assessment of the event to the study drug, according to the following definitions:

Unrelated	• The event started in no temporal relationship to medicinal product applied and<br>• The event can be definitely explained by underlying diseases or other situations.
Related	• The event started in a plausible temporal relationship to medicinal product applied and<br>• The event cannot be definitely explained by underlying diseases or other situations.

Unexpected Adverse Drug Reaction

An “unexpected” adverse drug reaction is an adverse reaction, the nature or severity of which is not consistent with the Investigator Brochure [ICH E2A].

Suspected Unexpected Serious Adverse Reactions (SUSARs)

A serious adverse reaction, the nature or severity of which is suspected to be not consistent with the Investigator’s Brochure. The Sponsor evaluates any SAE that has been reported regarding seriousness, causality and expectedness. If the event is related to the investigational product and is both serious and unexpected, it is classified as a SUSAR.

Safety Signals

All suspected new risks and relevant new aspects of known adverse reactions that require safety-related measures.

Assessment of Severity

All AEs, serious and non-serious, will be fully documented in the appropriate CRF. For each AE, the investigator will provide the onset, duration, intensity, treatment required, outcome and action taken with the investigational product.

The intensity of AEs will be assessed as being

- mild (hardly noticeable, negligible impairment of well-being),
- moderate (marked discomfort, but tolerable without immediate relief), or
- severe (overwhelming discomfort, calling for immediate relief).

The investigator will determine the relationship of the investigational drug to all AEs as defined on the AE page of the CRF.

10.2. Reporting of serious adverse events (SAE) and other safety related events

Reporting of SAEs

All SAEs must be reported immediately and within a maximum of 24 hours to the Sponsor of the study. The Sponsor will re-evaluate the SAE and return the form to the site.

SAEs resulting in death are reported to the Ethics Committee via BASEC within 7 days.

Reporting of SUSARs

A SUSAR needs to be reported to the Ethics Committee (local event via local Investigator) via BASEC and to Swissmedic for category C study (via Sponsor) within 7 days, if the event is fatal, or within 15 days (all other events).

Reporting of Safety Signals

All suspected new risks and relevant new aspects of known adverse reactions that require safety-related measures, i.e. so called safety signals, must be reported to the Sponsor within 24 hours. The Sponsor

must report the safety signals within 7 days to the Ethics Committee (local event via local Investigator) via BASEC and to Swissmedic in case of a category B or C study.

Reporting and Handling of Pregnancies

Pregnant participants must immediately be withdrawn from the clinical study. Any pregnancy during the treatment phase of the study and within 30 days after discontinuation of study medication will be reported to the Sponsor within 24 hours. The course and outcome of the pregnancy should be followed up carefully, and any abnormal outcome regarding the mother or the child should be documented and reported.

Periodic reporting of safety

An annual safety report is submitted once a year to the local Ethics Committee via local Investigator and to Swissmedic in case of a category C study via Sponsor.

10.3. Follow up of (Serious) Adverse Events

Participants terminating the study (either regularly or prematurely) with

- reported ongoing SAE, or
- any ongoing AEs of laboratory values or of vital signs being beyond the alert limit

will return for a follow-up investigation. This visit will take place up to 30 days after terminating the treatment period. Follow-up information on the outcome will be recorded on the respective AE page in the CRF. All other information has to be documented in the source documents. Source data has to be available upon request.

In case of participants lost to follow-up, efforts should be made and documented to contact the participant to encourage him/her to continue study participation as scheduled. In case of minor AEs a telephone call to the participants may be acceptable.

All new SAE or pregnancies that the investigators will be notified of within 30 days after discontinuation of study medication have to be reported in appropriate report forms and in the CRF if required.

Follow-up investigations may also be necessary according to the investigator’s medical judgment even if the participant has no AE at the end of the study. However, information related to these investigations does not have to be documented in the CRF but must be noted in the source documents.

11. STATISTICAL METHODS

11.1 Hypothesis

The central hypothesis of testing is:

H1: average of daily standard units of alcohol consumed between visit 3 and visit 6 is different between the patients treated with psilocybin than patients treated with placebo.

The contrasting null hypothesis is accordingly:

H0: average of daily standard units of alcohol consumed between visit 3 and visit 6 is not different between the patients treated with psilocybin than patients treated with placebo.

11.2 Determination of Sample Size

Sample size was determined by power analysis (G-Power 3.1, repeated-measures ANOVA with between factor, alpha error probability = 0.05, power = 0.95) based on effect sizes reported by Bogenschutz et al. (2015) revealing a minimum sample size per treatment arm of n=24. Adjusting the sample size calculation for the conduction of one interim analysis according to the O'Brien-Fleming method does not change the minimum sample size per treatment arm of n=24. Therefore, the planned sample size is 30 participants per treatment arm.

11.3 Statistical criteria of termination of trial

11.3.1 Criteria of stopping for futility

The trial will not be stopped in case of futility, unless the sponsor or the principal investigator advices otherwise.

11.3.2 Criteria of stopping for harm

The stopping rules for harm in this trial are not statistically defined. The trial will be terminated by the sponsor or principal investigator if unanticipated problems associated with unexpected serious harm to trial participants or with possible risks of harm to participants have taken place.

11.4 Planned Analyses

11.4.1 Datasets to be analysed, analysis populations

The primary sample for analysis will be based on all patients who have completed all assessments up to visit 6. Pilot data will not be statistically analysed.

11.4.2 Primary Analysis

The primary analysis will be conducted by the trial statistician after reaching the full sample size of N=2x30 (completed all assessments up to visit 6). For the two treatment groups (placebo versus psilocybin), data will be analysed using a two-sample t-test. The significance level is set at 5%.

For the analysis comparing relapse rates between treatment groups, a Chi-Square test will be used.

11.4.3 Secondary Analyses

Comparisons between psilocybin and placebo will be performed using a mixed-model analysis of

variance (ANOVA) to compare secondary endpoint change from baseline at the different time points (1, 14, 28 days post-treatment). Results will be Bonferroni corrected for the number of comparisons. To assess Time to Relapse, a survival analysis will be conducted.

In addition, post hoc analyses on predictors of treatment response will be carried out. For these analyses, psychological (i.e. questionnaire scores, MET scores) and (neuro)biological measures (MRI measures, genetic and epigenetic data) will be used.

MRI data will be analysed using general lineal models (GLMs) and psychophysiological interaction models. For these analyses, the generally established methods of controlling for multiple comparisons will be applied.

11.4.4 Interim analyses

No interim analysis will be conducted.

11.4.5 Safety analysis

Although this study is not a systematic investigation of safety, this study will also clinically assess safety and tolerability of psilocybin treatment in alcohol dependent patients. The following safety endpoints will be clinically assessed by the investigators at each visit: occurrence of adverse events (AEs), psychological and physical well-being, vital signs, and use of concomitant medication.

11.4.6 Deviation(s) from the original statistical plan

Any deviations from the planned analyses will be discussed between the Sponsor and the responsible statistician. Any deviation will be clearly reported in the publications resulting from the project and will be documented in the data management system.

11.5 Handling of missing data and drop-outs

The primary efficacy analysis will be done using the last observation carried forward (LOCF) approach on the intention-to-treat population (ITT), i.e. all patients randomized who received psilocybin treatment and had at least one assessment of efficacy 28 days post-treatment will be analysed. Drop-outs before the primary efficacy assessment 28 days post-treatment will be replaced. The Observed Cases (OC) approach, in which only the observed values will be analysed, will be used for supportive analyses.

12. QUALITY ASSURANCE AND CONTROL

The Sponsor is implementing and maintaining quality assurance and quality control systems with written SOPs and Working Instructions to ensure that trials are conducted and data are generated, documented (record), and reported in compliance with the protocol, GCP, and applicable regulatory requirement(s). The Principal Investigators at must have a manual of the relevant SOPs and WIs for the study on site and is responsible for proper training of all involved study personnel for the respective procedures. Monitoring and Audits will be conducted during the course of the study for quality assurance purposes.

12.1 Data handling and record keeping / archiving

For the scientific analysis during the project, raw data of all participants will be kept in pseudonymous format on password-protected computers, by referencing subject-specific data with pseudonymous codes. Data analysis will be performed using pseudonomized data only. Subject-specific data (key) will be kept in a separate password-protected database, available only to the project staff and the database administrators. In case of data reuse, only pseudonymized data will be shared by the project staff. After closing of the study, the pseudonymization key will be stored at secretariat of the research department at the PUK, who is independent from the study team.

Prior to their enrolment, participants will be asked for consent concerning the potential re-use of their data. This serves to maximize the scientific value of the acquired data and to avoid unnecessary repetitions of measurements, thus reducing the burden on the participants should they wish to take part in future studies.

The study will strictly follow the protocol. If any changes become necessary, they must be laid down in an amendment to the protocol. All amendments of the protocol must be signed by the Sponsor and submitted to CEC and Swissmedic.

12.1.1 Case Report Forms

The investigators will use electronic case report forms (eCRF), one for each enrolled study participant, to be filled in with all relevant data pertaining to the participant during the study. All participants who either entered the study or were considered not-eligible or were eligible but not enrolled into the study additionally have to be documented on a screening log. The investigator will document the participation of each study participant on the enrolment log. The correct data must be inserted, (up)-dated and initialled by the investigator. Data that are not available or not done should be made clear by adding ‘not applicable’ (NA). A declaration ensuring accuracy of data recorded in the case report forms must be electronically signed by the investigator. eCRFs must be kept current to reflect participant status at each phase during the course of study. Participants must not to be identified in the eCRF by name. Appropriate coded identification (e.g. Participant Number) must be used. Initials must not be used in combination with the date of birth in the eCRF for identification of the study participant (combination of initials and year of birth possible).

It must be assured that any authorized person, who may perform data entries and changes in the eCRF, can be identified. A list with signatures and initials of all authorized persons will be filed in the study site file and the trial master file, respectively.

Documented medical histories will be maintained. These records will also include the following: laboratory and other medical test results (ECGs, MRIs.) which must be kept on file with the individual participant’s eCRF.

The investigators assure to perform a complete and accurate documentation of the participant data in the eCRF. All data entered into the eCRF must also be available in the individual participant file as printouts by either the investigator or another responsible person assigned by the investigator.

Essential documents must be retained for at least 10 years after the regular end or a premature termination of the respective study (KlinV Art. 45). Essential documents must be retained according to local law in case of international multicentre studies.

Any patient files and source data must be archived for the longest possible period of time according to the feasibility of the investigational site, e.g. hospital, institution or private practice.

12.1.2 Specification of source documents

The following documents are considered source data, including but not limited to:

- SAE worksheets

- Nurse records, records of clinical coordinators
- Medical records from other department(s), or other hospital(s), or discharge letters and correspondence with other departments/hospitals, if participant visited any during the study period and the post study period

Source data must be available at the site to document the existence of the study participants and substantiate the integrity of study data collected. Source data must include the original documents relating to the study, as well as the medical treatment and medical history of the participant.

The following information (at least but not limited to) should be included in the source documents:

- Demographic data (age, sex)
- Inclusion and Exclusion Criteria details
- Participation in study and signed and dated Informed Consent Forms
- Visit dates
- Medical history and physical examination details
- AEs and concomitant medication (SAE report form IMP)
- Results of relevant examinations
- Laboratory printouts
- Dispensing and return of study drug details (drug accountability log total, drug accountability per patient, drug destruction record)
- Reason for premature discontinuation
- Randomization number (subject randomization log)

12.1.3 Record keeping / archiving

All study data must be archived for a minimum of 10 years after study termination or premature termination of the clinical trial. Printed source documents will be stored in locked cabinets in an office which will be locked when the office is not in use. Identifying data (e.g., consent) will be stored in separate locked cabinets. Digitalized data will be securely stored on the research server. Voice recordings are also part of the clinical medical record. Following legal advice, this data needs to be stored for at least 10 years along with the rest of the medical record. This data and will be safely stored on the servers of the psychiatric hospital. All online access to this data is secured via a log-in and password protected system. Physical access is restricted by a key system. Blood and urine specimens used for diagnostic purposes and alcohol use markers will be destroyed after assessment. Blood specimens collected for the analysis of epigenetic markers will be archived in the Biobank of the Department of Genetic Epidemiology in Psychiatry, Central Institute of Mental Health, D-68159 Mannheim. Data of individuals who do not sign an informed consent form will be destroyed. Telephone screening data of participants who are not invited to the screening visit will be destroyed. Printed study data will be stored in the archives, digitalized data on the research server of the Department of Psychiatry, Psychotherapy and Psychosomatics, Zürich University Hospital for Psychiatry.

12.2 Data management

12.2.1 Data Management System

An electronic password restricted data management system will be used. The eCRF is hosted on research servers at the Clinical Trials Unit (CTU) at the University Bern. Only approved study personnel will have access to this system. Online Follow-Up data will be collected using the same electronic system (RedCap) and will be protected similarly.

12.2.2 Data security, access and back-up

Access to the study data will be restricted. A password system will be utilized to control access. Only

approved study staff, as listed in the study site personnel signature authorization form, will have access. Data generated as a result of this study are to be available for inspection on request by the monitors, by the CEC, Swissmedic, and the regulatory health authorities. All forms, diskettes and tapes related to study data will be kept in locked cabinets. All reports prepared by the approved study staff will be prepared such that no individual subject can be identified.

12.2.3 Analysis and archiving

Data are extracted from the electronic data management system via direct download from the research server at the CTC and will be stored on password-protected on-site computers. For the data management the software REDCap will be used. Data will be validated by the study team. The electronic data will be installed and archived at the CTC on a secure server pertaining to the University Hospital of Zurich. Participant files are to be stored in numerical order and stored in a secure and accessible place and manner at the Psychiatric University Hospital. Participant files will be maintained in storage for a period of 10 years after completion of the study.

12.2.4 Electronic and central data validation

Data integrity will be enforced through a variety of mechanisms. Data will be entered immediately after data generation. Study personnel will be responsible to verify that the data entered are in the proper format. The approved study staff will be responsible for making appropriate corrections to the data forms whenever any data item is changed. Written documentation of changes will be available via electronic logs and audit trails. Study personnel will be trained for data quality purposes before study begin.

12.3 Monitoring

Regular monitoring visits at the investigator’s site prior to the start and during the course of the study will help to follow up the progress of the clinical study, to assure utmost accuracy of the data and to detect possible errors at an early time point. The Sponsor organizes independent monitoring for the study.

All original data including all patient files, progress notes and copies of laboratory and medical test results must be available for monitoring. The monitor will review all or a part of the CRF and written informed consents. The accuracy of the data will be verified by reviewing the above referenced documents.

12.4 Audits and Inspections

A quality assurance audit/inspection of this study may be conducted by the competent authority or CEC, respectively. The quality assurance auditor/inspector will have access to all medical records, the investigator's study related files and correspondence, and the informed consent documentation that is relevant to this clinical study.

The investigator will allow the persons being responsible for the audit or the inspection to have access to the source data/documents and to answer any questions arising. All involved parties will keep the patient data strictly confidential.

12.5 Confidentiality, Data Protection

Direct access to source documents will be permitted for purposes of monitoring, audits and inspections. The PI and the investigators will have access to protocol, dataset, statistical code, etc. during and after the study.

12.6 Storage of biological material and related health data

Urine specimens will be used for diagnostic purposes only and will be destroyed after assessment. Blood specimens collected at visit 1 (Routine Blood analysis and markers for alcohol use) and visit 6 (markers for alcohol use) will be used for diagnostic purposes only and will be destroyed after assessment. Blood samples collected at visit 1, 4, and 6 for genetic, epigenetic, and induced pluripotent stem cells will be securely stored at the Psychiatric University Hospital Zürich for the duration of the study. After data collection is finished for all participants, blood samples will be shipped to PD Dr. Stephanie Witt, Head of the Molecular Genetic Laboratory and Biobank, Dept. of Genetic Epidemiology in Psychiatry, Central Institute of Mental Health, D-68159 Mannheim for analyses using the form provided in Annex 1. After analyses the samples will be stored at the Molecular Genetic Laboratory and Biobank, Dept. of Genetic Epidemiology in Psychiatry, Central Institute of Mental Health, D-68159 Mannheim for an undetermined amount of time. Patients will receive additional consent forms to consent to the use and storage of their blood samples (see informed consent form).

13. PUBLICATION AND DISSEMINATION POLICY

After the statistical analysis of this trial the sponsor and principal investigator will make every endeavour to publish the data in a medical journal.

14. FUNDING AND SUPPORT

14.1 Funding

This project is supported with 281'763 CHF by the Swiss National Science Foundation, under the framework of Neuron Cofund (Grant to Prof. Dr. Franz X. Vollenweider). The funders have no role in study design, data collection and analysis, decision to publish, or preparation of a manuscript. Three independent animal studies will address complementary mechanistic questions in mice. These research projects are independent and the research groups have no role in study design, data collection or analysis of the current study.

15. INSURANCE

Insurance is covered by “Versicherung für klinische Versuche der Zürich Versicherungs-Gesellschaft AG, Mythenquai 2, 8002 Zürich” for Psychiatric University Hospital Zurich (Policy no.: 14.970.885).
Any damage developed in relation to study participation is covered by this insurance. So as not to forfeit their insurance cover, the participants themselves must strictly follow the instructions of the study personnel. Participants must not be involved in any other medical treatment without permission of the principal investigator (emergency excluded). Medical emergency treatment must be reported immediately to the investigator. The investigator must also be informed instantly, in the event of health problems or other damages during or after the course of study treatment.
The investigator will allow delegates of the insurance company to have access to the source data/documents as necessary to clarify a case of damage related to study participation. All involved parties will keep the patient data strictly confidential.

A copy of the insurance certificate will be placed in the Investigator’s Site File.

16. REFERENCES

1. Declaration of Helsinki, Version October 2013, (http://www.wma.net/en/30publications/10policies/b3/index.html)
2. International Conference on Harmonization (ICH, 1996) E6 Guideline for Good Clinical Practice. (http://www.ich.org/fileadmin/Public_Web_Site/ICH_Products/Guidelines/Efficacy/E6/E6_R2__Step_4.pdf)
3. International Conference on Harmonization (ICH, 1997) E8 Guideline: General Considerations for Clinical Trials http://www.ich.org/fileadmin/Public_Web_Site/ICH_Products/Guidelines/Efficacy/E8/Step4/E8_Guideline.pdf)
4. Humanforschungsgesetz, HFG Bundesgesetz über die Forschung am Menschen (Bundesgesetz über die Forschung am Menschen, HFG) vom 30. September 2011/ Loi fédérale relative à la recherche sur l'être humain (loi relative à la recherche sur l'être humain, LRH) du 30 septembre 2011 / Legge federale concernente la ricerca sull'essere umano (Legge sulla ricerca umana, LRUm) del 30 settembre 2011
5. Verordnung über klinische Versuche in der Humanforschung (Verordnung über klinische Versuche, KlinV) vom 20. September 2013 / Ordonnance sur les essais cliniques dans le cadre de la recherche sur l'être humain (Ordonnance sur les essais cliniques, OClin) du 20 septembre 2013. Ordinanza sulle sperimentazioni cliniche nella ricerca umana (Ordinanza sulle sperimentazioni cliniche, OSRUm) del 20 settembre 2013
6. Heilmittelgesetz, HMG Bundesgesetz über Arzneimittel und Medizinprodukte (Heilmittelgesetz, HMG) vom 15. Dezember 2000 / Loi fédérale sur les médicaments et les dispositifs médicaux (Loi sur les produits thérapeutiques, LPT) du 15 décembre 2000 / Legge federale sui medicamenti e i dispositivi medici (Legge sugli agenti terapeutici, LATer)
7. ISO 14155:2011 Clinical investigation of medical devices for human subjects -- Good clinical practice (www.iso.org)
8. ISO 10993 Biological evaluation of medical devices (www.iso.org)
9. MEDDEV 2.7/3 revision 3, May 2015
10. Medizinprodukteverordnung (MepV) vom 17. Oktober 2001 / Ordonnance sur les dispositifs médicaux (ODim) du 17 octobre 2001 / Ordinanza relativa ai dispositivi medici (ODmed) del 17 ottobre 2001
11. WHO, International Clinical Trials Registry Platform (ICTRP) (http://www.who.int/ictrp/en/)
12. European regulation on medical devices 2017/745.
13. Strahlenschutzverordnung (StSV) vom 26. April 2017 / Ordonnance sur la radioprotection (ORaP) du 26 avril 2017 / Ordinanza sulla radioprotezione (ORaP) del 26 aprile 2017.

Abi-Dargham, A., & Horga, G. (2016). The search for imaging biomarkers in psychiatric disorders. *Nat Med, 22*(11), 1248-1255. doi:10.1038/nm.4190

Baumeister, D., Barnes, G., Giaroli, G., & Tracy, D. (2014). Classical hallucinogens as antidepressants? A review of pharmacodynamics and putative clinical roles. *Ther Adv Psychopharmacol, 4*(4), 156-169. doi:10.1177/2045125314527985

Berkel, T. D., & Pandey, S. C. (2017). Emerging Role of Epigenetic Mechanisms in Alcohol Addiction. *Alcohol Clin Exp Res, 41*(4), 666-680. doi:10.1111/acer.13338

Bogenschutz, M. P., Forcehimes, A. A., Pommy, J. A., Wilcox, C. E., Barbosa, P. C., & Strassman, R. J. (2015). Psilocybin-assisted treatment for alcohol dependence: a proof-of-concept study. *J Psychopharmacol, 29*(3), 289-299. doi:10.1177/0269881114565144

Brandon, T. H., Vidrine, J. I., & Litvin, E. B. (2007). Relapse and relapse prevention. *Annu Rev Clin Psychol, 3*, 257-284. doi:10.1146/annurev.clinpsy.3.022806.091455

Bryant, F. B. (2003). Savoring beliefs inventory (SBI): A scale for measuring beliefs about savouring. *Journal of Mental Health, 12*(2), 175-196. doi:10.1080/0963823031000103489

Camchong, J., Stenger, A., & Fein, G. (2013). Resting-State Synchrony During Early Alcohol Abstinence Can Predict Subsequent Relapse. *Cerebral Cortex, 23*(9), 2086-2099. doi:10.1093/cercor/bhs190

Carhart-Harris, R. L., Bolstridge, M., Rucker, J., Day, C. M., Erritzoe, D., Kaelen, M., . . . Nutt, D. J. (2016). Psilocybin with psychological support for treatment-resistant depression: an open-label feasibility study. *Lancet Psychiatry, 3*(7), 619-627. doi:10.1016/S2215-0366(16)30065-7

Carhart-Harris, R. L., Erritzoe, D., Williams, T., Stone, J. M., Reed, L. J., Colasanti, A., . . . Nutt, D. J.

(2012). Neural correlates of the psychedelic state as determined by fMRI studies with psilocybin. *Proc Natl Acad Sci U S A, 109*(6), 2138-2143.

Carhart-Harris, R. L., Leech, R., Williams, T. M., Erritzoe, D., Abbasi, N., Bargiotas, T., . . . Nutt, D. J. (2012). Implications for psychedelic-assisted psychotherapy: functional magnetic resonance imaging study with psilocybin. *Br J Psychiatry, 200*(3), 238-244. doi:10.1192/bjp.bp.111.103309

Collaborators, G. B. D. R. F. (2017). Global, regional, and national comparative risk assessment of 84 behavioural, environmental and occupational, and metabolic risks or clusters of risks, 1990-2016: a systematic analysis for the Global Burden of Disease Study 2016. *Lancet, 390*(10100), 1345-1422. doi:10.1016/S0140-6736(17)32366-8

De Cesarei, A., & Codispoti, M. (2006). When does size not matter? Effects of stimulus size on affective modulation. *Psychophysiology, 43*(2), 207-215. doi:10.1111/j.1469-8986.2006.00392.x

Deusinger, I. M. (1982). [Measurement of change in self-concept with the Frankfurt self-concept scales]. *Z Gerontol, 15*(1), 42-45.

DiClemente, C. C., Carbonari, J. P., Montgomery, R. P., & Hughes, S. O. (1994). The Alcohol Abstinence Self-Efficacy scale. *J Stud Alcohol, 55*(2), 141-148.

Dittrich, A. (1998). The standardized psychometric assessment of altered states of consciousness (ASCs) in humans. *Pharmacopsychiatry, 31 Suppl 2*, 80-84.

Doyle, O. M., Mehta, M. A., & Brammer, M. J. (2015). The role of machine learning in neuroimaging for drug discovery and development. *Psychopharmacology (Berl), 232*(21-22), 4179-4189. doi:10.1007/s00213-015-3968-0

Due, D. L., Huettel, S. A., Hall, W. G., & Rubin, D. C. (2002). Activation in mesolimbic and visuospatial neural circuits elicited by smoking cues: evidence from functional magnetic resonance imaging. *Am J Psychiatry, 159*(6), 954-960. doi:10.1176/appi.ajp.159.6.954

Fede, S. J., Grodin, E. N., Dean, S. F., Diazgranados, N., & Momenan, R. (2019). Resting state connectivity best predicts alcohol use severity in moderate to heavy alcohol users. *Neuroimage-Clinical, 22*. doi:UNSP 101782

10.1016/j.nicl.2019.101782

Flannery, B. A., Volpicelli, J. R., & Pettinati, H. M. (1999). Psychometric properties of the Penn Alcohol Craving Scale. *Alcohol Clin Exp Res, 23*(8), 1289-1295.

Forcehimes, A. A., Tonigan, J. S., Miller, W. R., Kenna, G. A., & Baer, J. S. (2007). Psychometrics of the Drinker Inventory of Consequences (DrInC). *Addictive Behaviors, 32*(8), 1699-1704. doi: https://doi.org/10.1016/j.addbeh.2006.11.009

Fox, M. D., & Raichle, M. E. (2007). Spontaneous fluctuations in brain activity observed with functional magnetic resonance imaging. *Nat Rev Neurosci, 8*(9), 700-711.

Franke, G. (1995). *Die Symptom-Check-Liste von Derogatis - Deutsche Version*. Göttingen, Germany: Beltz Test Gesellschaft.

Franz, M., Lemke, M. R., Meyer, T., Ulferts, J., Puhl, P., & Snaith, R. P. (1998). Deutsche Version der Snaith-Hamilton-Pleasure-Scale (SHAPS-D). Erfassung von Anhedonie bei schizophrenen und depressiven Patienten. *Fortschr Neurol Psychiatr, 66*(9), 407–413.

Gable, R. S. (2004). Comparison of acute lethal toxicity of commonly abused psychoactive substances. *Addiction, 99*(6), 686-696. doi:10.1111/j.1360-0443.2004.00744.x

Garcia-Romeu, A., Davis, A. K., Erowid, F., Erowid, E., Griffiths, R. R., & Johnson, M. W. (2019). Cessation and reduction in alcohol consumption and misuse after psychedelic use. *J Psychopharmacol*, 269881119845793. doi:10.1177/0269881119845793

George, M. S., Anton, R. F., Bloomer, C., Teneback, C., Drobes, D. J., Lorberbaum, J. P., . . . Vincent, D. J. (2001). Activation of prefrontal cortex and anterior thalamus in alcoholic subjects on exposure to alcohol-specific cues. *Arch Gen Psychiatry, 58*(4), 345-352.

Griffiths, R. R., Johnson, M. W., Carducci, M. A., Umbricht, A., Richards, W. A., Richards, B. D., . . . Klinedinst, M. A. (2016). Psilocybin produces substantial and sustained decreases in depression and anxiety in patients with life-threatening cancer: A randomized double-blind trial. *Journal of Psychopharmacology, 30*(12), 1181-1197. doi:10.1177/0269881116675513

Griffiths, R. R., Richards, W. A., McCann, U., & Jesse, R. (2006). Psilocybin can occasion mystical-type experiences having substantial and sustained personal meaning and spiritual significance. *Psychopharmacology (Berl), 187*(3), 268-283; discussion 284-292. doi:10.1007/s00213-006-0457-5

Grinspoon, L., & Bakalar, J. B. (1981). The psychedelic drug therapies. *Curr Psychiatr Ther, 20*, 275-283.

Grob, C. S., Danforth, A. L., Chopra, G. S., Hagerty, M., McKay, C. R., Halberstadt, A. L., & Greer, G. R. (2011). Pilot study of psilocybin treatment for anxiety in patients with advanced-stage cancer. *Arch Gen Psychiatry, 68*(1), 71-78. doi:10.1001/archgenpsychiatry.2010.116

Grusser, S. M., Wrase, J., Klein, S., Hermann, D., Smolka, M. N., Ruf, M., . . . Heinz, A. (2004). Cue-induced activation of the striatum and medial prefrontal cortex is associated with subsequent relapse in abstinent alcoholics. *Psychopharmacology, 175*(3), 296-302. doi:10.1007/s00213-004-1828-4

Halpern, J. H., & Pope, H. G., Jr. (2003). Hallucinogen persisting perception disorder: what do we know after 50 years? *Drug Alcohol Depend, 69*(2), 109-119.

Hamilton, M. (1959). The assessment of anxiety states by rating. *Br J Med Psychol, 32*(1), 50-55.

Hampton, W. H., Hanik, I. M., & Olson, I. R. (2019). Substance abuse and white matter: Findings, limitations, and future of diffusion tensor imaging research. *Drug and alcohol dependence*. doi: 10.1016/j.drugalcdep.2019.02.005

Hasler, F., Grimberg, U., Benz, M. A., Huber, T., & Vollenweider, F. X. (2004). Acute psychological and physiological effects of psilocybin in healthy humans: a double-blind, placebo-controlled dose-effect study. *Psychopharmacology (Berl), 172*(2), 145-156.

Heinz, A., Beck, A., Grusser, S. M., Grace, A. A., & Wrase, J. (2009). Identifying the neural circuitry of alcohol craving and relapse vulnerability. *Addict Biol, 14*(1), 108-118. doi:10.1111/j.1369-1600.2008.00136.x

Hermann, D., Smolka, M. N., Wrase, J., Klein, S., Nikitopoulos, J., Georgi, A., . . . Heinz, A. (2006). Blockade of cue-induced brain activation of abstinent alcoholics by a single administration of amisulpride as measured with fMRI. *Alcoholism-Clinical and Experimental Research, 30*(8), 1349-1354. doi:10.1111/j.1530-0277.2006.00174.x

Holloway, T., & Gonzalez-Maeso, J. (2015). Epigenetic Mechanisms of Serotonin Signaling. *ACS Chem Neurosci, 6*(7), 1099-1109. doi:10.1021/acschemneuro.5b00033

Insel, T. R., Voon, V., Nye, J. S., Brown, V. J., Altevogt, B. M., Bullmore, E. T., . . . Sahakian, B. J. (2013). Innovative solutions to novel drug development in mental health. *Neurosci Biobehav Rev, 37*(10 Pt 1), 2438-2444. doi:10.1016/j.neubiorev.2013.03.022

Jasinska, A. J., Stein, E. A., Kaiser, J., Naumer, M. J., & Yalachkov, Y. (2014). Factors modulating neural reactivity to drug cues in addiction: a survey of human neuroimaging studies. *Neurosci Biobehav Rev, 38*, 1-16. doi:10.1016/j.neubiorev.2013.10.013

Johnson, M., Richards, W., & Griffiths, R. (2008). Human hallucinogen research: guidelines for safety. *J Psychopharmacol, 22*(6), 603-620. doi:10.1177/0269881108093587

Johnson, M. W., Garcia-Romeu, A., Cosimano, M. P., & Griffiths, R. R. (2014). Pilot study of the 5-HT2AR agonist psilocybin in the treatment of tobacco addiction. *J Psychopharmacol, 28*(11), 983-992. doi:10.1177/0269881114548296

Kometer, M., Schmidt, A., Jancke, L., & Vollenweider, F. X. (2013). Activation of serotonin 2A receptors underlies the psilocybin-induced effects on alpha oscillations, N170 visual-evoked potentials, and visual hallucinations. *J Neurosci, 33*(25), 10544-10551. doi:10.1523/JNEUROSCI.3007-12.2013

Kraehenmann, R., Preller, K. H., Scheidegger, M., Pokorny, T., Bosch, O. G., Seifritz, E., & Vollenweider, F. X. (2015). Psilocybin-Induced Decrease in Amygdala Reactivity Correlates with Enhanced Positive Mood in Healthy Volunteers. *Biol Psychiatry, 78*(8).

Kraehenmann, R., Schmidt, A., Friston, K., Preller, K. H., Seifritz, E., & Vollenweider, F. X. (2016). The mixed serotonin receptor agonist psilocybin reduces threat-induced modulation of amygdala connectivity. *Neuroimage Clin, 11*, 53-60. doi:10.1016/j.nicl.2015.08.009

Krampen, G. (1994). Skalen zur Erfassung von Hoffnungslosigkeit (H-Skalen). Handanweisung. Göttingen: Hogrefe.

Krampen, G., & Wald, B. (2001). Kurzinstrumente für die Prozessevaluation und adaptive Indikation in der Allgemeinen und Differentiellen Psychotherapie und Beratung. *Diagnostica, 47*(1), 43-50. doi:10.1026//0012-1924.47.1.43

Krebs, T. S., & Johansen, P. O. (2012). Lysergic acid diethylamide (LSD) for alcoholism: meta-analysis of randomized controlled trials. *J Psychopharmacol, 26*(7), 994-1002.

doi:10.1177/0269881112439253

Kuhner, C., Burger, C., Keller, F., & Hautzinger, M. (2007). [Reliability and validity of the Revised Beck Depression Inventory (BDI-II). Results from German samples]. *Nervenarzt, 78*(6), 651-656. doi:10.1007/s00115-006-2098-7

Kyzar, E. J., Nichols, C. D., Gainetdinov, R. R., Nichols, D. E., & Kalueff, A. V. (2017). Psychedelic Drugs in Biomedicine. *Trends Pharmacol Sci, 38*(11), 992-1005. doi:10.1016/j.tips.2017.08.003

Lang, P. J., Bradley, M. M., & Cuthbert, B. N. (1997). International Affective Picture System (IAPS): Technical Manual and Affective Ratings. . *NIMH Center for the Study of Emotion and Attention*(1), 39-58.

Leuner, H. (1982). Intensivierung der tiefenpsychologischen Psychotherapie durch Medikamente. In D. Eicke (Ed.), *Die Psychologie des 20. Jahrhunderts* (Vol. 3, pp. 1197-1209). Zürich: Kindler.

Lewis, C. R., Preller, K. H., Kraehenmann, R., Michels, L., Staempfli, P., & Vollenweider, F. X. (2017). Two dose investigation of the 5-HT-agonist psilocybin on relative and global cerebral blood flow. *Neuroimage, 159*, 70-78. doi:10.1016/j.neuroimage.2017.07.020

Litten, R. Z., Castle, I. J., Falk, D., Ryan, M., Fertig, J., Chen, C. M., & Yi, H. Y. (2013). The placebo effect in clinical trials for alcohol dependence: an exploratory analysis of 51 naltrexone and acamprosate studies. *Alcohol Clin Exp Res, 37*(12), 2128-2137. doi:10.1111/acer.12197

Litten, R. Z., Falk, D. E., Ryan, M. L., Fertig, J., & Leggio, L. (2018). Advances in Pharmacotherapy Development: Human Clinical Studies. *Handb Exp Pharmacol, 248*, 579-613. doi:10.1007/164_2017_79

Moreno, F. A., Wiegand, C. B., Taitano, E. K., & Delgado, P. L. (2006). Safety, tolerability, and efficacy of psilocybin in 9 patients with obsessive-compulsive disorder. *J Clin Psychiatry, 67*(11), 1735-1740.

Myrick, H., Anton, R. F., Li, X., Henderson, S., Drobes, D., Voronin, K., & George, M. S. (2004). Differential brain activity in alcoholics and social drinkers to alcohol cues: relationship to craving. *Neuropsychopharmacology, 29*(2), 393-402. doi:10.1038/sj.npp.1300295

Myrick, H., Anton, R. F., Li, X., Henderson, S., Randall, P. K., & Voronin, K. (2008). Effect of naltrexone and ondansetron on alcohol cue-induced activation of the ventral striatum in alcohol-dependent people. *Archives of General Psychiatry, 65*(4), 466-475. doi:DOI 10.1001/archpsyc.65.4.466

Nandrino, J. L., Gandolphe, M. C., & El Haj, M. (2017). Autobiographical memory compromise in individuals with alcohol use disorders: Towards implications for psychotherapy research. *Drug and Alcohol Dependence, 179*, 61-70. doi:10.1016/j.drugalcdep.2017.06.027

Nichols, D. E. (2004). Hallucinogens. *Pharmacol Ther, 101*(2), 131-181.

Organisation, W. H. (2019). https://www.who.int/healthinfo/survey/whoqol-qualityoflife/en/.

Pandey, A. K., Ardekani, B. A., Kamarajan, C., Zhang, J., Chorlian, D. B., Byrne, K. N. H., ... & Porjesz, B. (2018). Lower Prefrontal and Hippocampal Volume and Diffusion Tensor Imaging Differences Reflect Structural and Functional Abnormalities in Abstinent Individuals with Alcohol Use Disorder. *Alcoholism: Clinical and Experimental Research*, *42*(10), 1883-1896.doi: 10.1111/acer.13854

Passie, T., Seifert, J., Schneider, U., & Emrich, H. M. (2002). The pharmacology of psilocybin. *Addiction Biology, 7*(4), 357-364. doi:10.1080/1355621021000005937

Pokorny, T., Preller, K. H., Kometer, M., Dziobek, I., & Vollenweider, F. X. (2017). Effect of Psilocybin on Empathy and Moral Decision-Making. *Int J Neuropsychopharmacol, 20*(9), 747-757. doi:10.1093/ijnp/pyx047

Preller, K. H., Burt, J. B., Ji, J. L., Schleifer, C. H., Adkinson, B. D., Stampfli, P., . . . Anticevic, A. (2018). Changes in global and thalamic brain connectivity in LSD-induced altered states of consciousness are attributable to the 5-HT2A receptor. *Elife, 7*. doi:10.7554/eLife.35082

Preller, K. H., Duerler, P., Burt, J. B., Ji, J. L., Adkinson, B. D., Staempfli, P., . . . Vollenweider, F. X. (2019). Psilocybin induces time-dependent changes in global functional connectivity. *submitted*.

Preller, K. H., Pokorny, T., Hock, A., Kraehenmann, R., Stampfli, P., Seifritz, E., . . . Vollenweider, F. X. (2016). Effects of serotonin 2A/1A receptor stimulation on social exclusion processing. *Proc Natl Acad Sci U S A, 113*(18), 5119-5124. doi:10.1073/pnas.1524187113

Preller, K. H., Razi, A., Zeidman, P., Stampfli, P., Friston, K. J., & Vollenweider, F. X. (2019). Effective connectivity changes in LSD-induced altered states of consciousness in humans. *Proc Natl Acad Sci U S A, 116*(7), 2743-2748. doi:10.1073/pnas.1815129116

Preller, K. H., & Vollenweider, F. X. (2018). Phenomenology, Structure, and Dynamic of Psychedelic States. *Curr Top Behav Neurosci, 36*, 221-256. doi:10.1007/7854_2016_459

Rehm, J., Anderson, P., Barry, J., Dimitrov, P., Elekes, Z., Feijao, F., . . . Gmel, G. (2015). Prevalence of and potential influencing factors for alcohol dependence in Europe. *Eur Addict Res, 21*(1), 6-18. doi:10.1159/000365284

Ross, S., Bossis, A., Guss, J., Agin-Liebes, G., Malone, T., Cohen, B., . . . Schmidt, B. L. (2016). Rapid and sustained symptom reduction following psilocybin treatment for anxiety and depression in patients with life-threatening cancer: a randomized controlled trial. *J Psychopharmacol, 30*(12), 1165-1180. doi:10.1177/0269881116675512

Satow, L. (2012). https://www.drsatow.de/tests/stress-und-coping-inventar/.

Schneider, F., Habel, U., Wagner, M., Franke, P., Salloum, J. B., Shah, N. J., . . . Zilles, K. (2001). Subcortical correlates of craving in recently abstinent alcoholic patients. *Am J Psychiatry, 158*(7), 1075-1083. doi:10.1176/appi.ajp.158.7.1075

Simon, J. J., Zimmermann, J., Cordeiro, S. A., Marée, I., Gard, D. E., Friederich, H.-C., . . . Kaiser, S. (2018). Psychometric evaluation of the Temporal Experience of Pleasure Scale (TEPS) in a German sample. *Psychiatry Research, 260*, 138-143. doi:https://doi.org/10.1016/j.psychres.2017.11.060

Smolka, M. N., Buhler, M., Klein, S., Zimmermann, U., Mann, K., Heinz, A., & Braus, D. F. (2006). Severity of nicotine dependence modulates cue-induced brain activity in regions involved in motor preparation and imagery. *Psychopharmacology (Berl), 184*(3-4), 577-588. doi:10.1007/s00213-005-0080-x

Sobell, L. C., & Sobell, M. B. (1995). *Aclohol Timeline Followback Users' Manual*. Toronto, Canada: Addiction Research Foundation.

Spanagel, R. (2009). Alcoholism: a systems approach from molecular physiology to addictive behavior. *Physiol Rev, 89*(2), 649-705. doi:10.1152/physrev.00013.2008

Spanagel, R., & Vengeliene, V. (2013). New pharmacological treatment strategies for relapse prevention. *Curr Top Behav Neurosci, 13*, 583-609. doi:10.1007/7854_2012_205

Spreng, R. N., & Grady, C. L. (2010). Patterns of Brain Activity Supporting Autobiographical Memory, Prospection, and Theory of Mind, and Their Relationship to the Default Mode Network. *Journal of Cognitive Neuroscience, 22*(6), 1112-1123. doi:DOI 10.1162/jocn.2009.21282

Starosta, A. N., Leeman, R. F., & Volpicelli, J. R. (2006). The BRENDA model: integrating psychosocial treatment and pharmacotherapy for the treatment of alcohol use disorders. *J Psychiatr Pract, 12*(2), 80-89.

Studerus, E., Gamma, A., Kometer, M., & Vollenweider, F. X. (2012). Prediction of Psilocybin Response in Healthy Volunteers. *PLoS One, 7*(2). doi:ARTN e3080010.1371/journal.pone.0030800

Studerus, E., Kometer, M., Hasler, F., & Vollenweider, F. X. (2011). Acute, subacute and long-term subjective effects of psilocybin in healthy humans: a pooled analysis of experimental studies. *J Psychopharmacol, 25*(11), 1434-1452.

Tyls, F., Palenicek, T., & Horacek, J. (2014). Psilocybin--summary of knowledge and new perspectives. *Eur Neuropsychopharmacol, 24*(3), 342-356.

Umbricht, D., Vollenweider, F. X., Schmid, L., Grubel, C., Skrabo, A., Huber, T., & Koller, R. (2003). Effects of the 5-HT2A agonist psilocybin on mismatch negativity generation and AX-continuous performance task: implications for the neuropharmacology of cognitive deficits in schizophrenia. *Neuropsychopharmacology, 28*(1), 170-181. doi:10.1038/sj.npp.1300005

Vollenweider, F. X., & Kometer, M. (2010). The neurobiology of psychedelic drugs: implications for the treatment of mood disorders. *Nat Rev Neurosci, 11*(9), 642-651.

Vollstadt-Klein, S., Loeber, S., Kirsch, M., Bach, P., Richter, A., Buhler, M., . . . Kiefer, F. (2011). Effects of cue-exposure treatment on neural cue reactivity in alcohol dependence: a randomized trial. *Biol Psychiatry, 69*(11), 1060-1066. doi:10.1016/j.biopsych.2010.12.016

Wrase, J., Grusser, S. M., Klein, S., Diener, C., Hermann, D., Flor, H., . . . Heinz, A. (2002). Development of alcohol-associated cues and cue-induced brain activation in alcoholics. *Eur Psychiatry, 17*(5), 287-291.

Yardley, M. M., & Ray, L. A. (2017). Medications development for the treatment of alcohol use disorder: insights into the predictive value of animal and human laboratory models. *Addict Biol, 22*(3), 581-615. doi:10.1111/adb.12349
