# Supplementary material for: Psilocybin-assisted therapy for relapse prevention in alcohol use disorder: a phase 2 randomized clinical trial
Source: eClinicalMedicine. 2025 Mar 14;82:103149. doi: 10.1016/j.eclinm.2025.103149 (PMC11937691; doi:10.1016/j.eclinm.2025.103149)
Supplement: Supplementary materials [file mmc2.pdf]

Supplementary Material

Table of content

Methods....2
Study Procedure....2
Psychotherapy....2
Measures....2
Statistical Analysis....4
Missing Data....4
Protocol Deviations....4
Illicit drug use within 3- and 6-month follow-up period....5
Psychoactive drug use within 3- and 6-month follow-up period....5
Additional therapeutic support....5
Results....6
Inclusion and exclusion criteria....9
Supplementary Figures....11
Supplementary Tables....25
References....36

Methods

Study Procedure

Recruitment was conducted through clinical institutions specialized in the treatment of substance use disorder, clinical trial networks (NCT04141501), social media, and advertisements in public places. While participants had to stop drinking alcohol before study inclusion, 29% of participants in the psilocybin and 37% in the placebo group drank alcohol on single occasions (on less than 5 days) between the screening and the dosing visit. Prior to drug administration, participants were required to provide negative alcohol breath samples and abstain from alcohol consumption for at least 24 hours. It was not obligatory for participants to commit to future abstinence, but they needed at least to aim to reduce their alcohol use substantially. Additionally, participants discontinued all psychotropic and serotonergic medications for at least five days before the dosing visit and suspended any ongoing psychotherapeutic treatment from third parties between study enrollment and the 4-week follow-up.

Psychotherapy

Our psychotherapeutic interventions are grounded in the BRENDA approach, which is rooted in the principles of Motivational Interviewing (MI). The BRENDA model involves: (1) conducting a biopsychosocial evaluation; (2) providing the patient with a summary of the evaluation findings; (3) demonstrating empathy; (4) addressing the patient's needs; (5) offering direct advice; and (6) evaluating the patient's response to the advice and adjusting the treatment plan accordingly [1]. The BRENDA approach was specifically developed to be used alongside pharmacological treatments for Alcohol Use Disorder (AUD) and has been effectively implemented in various clinical trials testing pharmacotherapies in substance use disorders [2,3].

In our study, we adapted the BRENDA approach to incorporate elements specific to psychedelic-assisted therapy. During the initial visit (1 hour), we assessed the patient's medical history and reasons for alcohol use. The preparation visit (1.5 hour) focused on preparing the patient for the dosing visit by discussing their previous experiences with psychedelics, expectations, concerns, and strategies for managing challenging situations, as well as the potential effects of psilocybin. Patients also set intentions for the session and goals for their future. On the dosing visit, we encouraged patients to focus inward, to fully engage with whatever arises — even if it is challenging or unpleasant — while avoiding judgment. On the following day, patients and their therapist reviewed the dosing visit, including discussions of any difficult situations and emotions encountered (1 hour). The visits at 2-week and 4-week follow-up (1 hour each) were dedicated to interpreting and integrating the experience and transferring it to behavioral and alcohol use changes in daily life. In total, participants underwent five therapy sessions with a total amount of 5.5 hours of therapy (excluding dosing visit).

Measures

Blood markers of alcohol consumption

To objectively assess changes in alcohol consumption patterns, we analyzed specific blood alcohol markers. Given alcohol's effects on liver function, we utilized the following markers as indicators of alcohol consumption: aspartate aminotransferase (ASAT/GOT), alanine aminotransferase (ALAT/GPT), and gamma-glutamyltransferase (Gamma-GT). These enzymes are primarily found in the liver and play a key role in identifying liver-related diseases.

Penn Alcohol Craving Scale (PACS)

Craving was captured by the PACS [4], which is a self-report measure consisting of five items, evaluating craving by the frequency, intensity, and duration of cravings, along with the individual's ability to resist drinking and an overall rating of their craving for alcohol. Each question is rated on a scale of 0 to 6, resulting in a maximum total score of 30.

Alcohol Abstinence Self-Efficacy Scale (AASE)

Abstinence self-efficacy was assessed by the AASE scale [5]. The scale utilizes a self-rated 5-point Likert scale to observe levels in abstaining from alcohol across 20 distinct high-risk scenarios. It also assesses the temptation to drink in each of these situations. Participants assign ratings on a scale of 1 to 5, with a minimum sum score of 20 and maximum sum score of 100 achievable in each subscale.

Beck's Depression Inventory (BDI)

Changes in depressive symptoms were assessed with the 21-item BDI, a widely used scale in clinical research [6,7]. The total score on this scale, ranging from 0 to 63, assesses the severity of depressive symptoms. Scores falling within the range of 10 to 19 denote mild depression, while those between 20 and 30 indicate moderate depression. Scores exceeding 30 signify severe depression, whereas a score below 10 suggests a remission of symptoms [8].

Beck Hopelessness-Scale (HS)

The 20-item true-false self-report inventory evaluates negative expectations about oneself, the environment, and the future in a clinical sample [9,10]. A sum score of 0-3 indicates no or minimal hopelessness, 4-8 indicates mild hopelessness, 9-14 indicates moderate hopelessness, and 15-20 indicates severe hopelessness.

Emotion Regulation Questionnaire (ERQ)

We employed the 10-item ERQ self-report measure to evaluate two distinct emotion regulation strategies: cognitive reappraisal (6 items, range: 6-42) and expressive suppression (4 items, range: 4-24) [11]. Cognitive reappraisal is an adaptive emotion regulation strategy, whereas expressive suppression is a maladaptive approach.

Positive and Negative Affect Schedule (PANAS)

The PANAS assesses both negative affect (10 items) and positive affect (10 items), and is used in both clinical and non-clinical samples [12,13]. Respondents rate these items on a 5-point Likert scale, ranging from 1 (not at all) to 5 (very much), resulting in a total sum of 10 to 50 for each subscale.

Quality of Life (WHOQOL-BREF)

To evaluate change in quality of life, we administered the WHOQOL-BREF questionnaire, comprising 26 items [14]. Four domains – environment (i.e., safety in daily life, financial resources), psychological health, physical health, and social relationships – were computed with the transformed score (range 4-20). Additionally, the total sum score was derived from all 26 items, incorporating 24 items associated with the four domains, along with two additional items concerning overall quality of life and general health (range 26-130). Higher scores indicate enhanced quality of life.

Snaith Hamilton Pleasure Scale (SHAPS)

The SHAPS evaluates present anhedonia through 14 items, with responses ranging from 0 (strongly disagree) to 4 (strongly agree) [15]. Scores of 3 or higher indicate abnormal levels of anhedonia, with a higher score indicating more pronounced anhedonia (score range: 0-14).

Temporary Experience of Pleasure scale (TEPS)

The TEPS consists of 18 items, measuring anticipatory and consummatory pleasure on a 6-point Likert scale [16]. The scale is divided into anticipatory (10 items, range: 10-60) and consummatory (8 items, range: 8-48) pleasure, with higher scores indicating a greater inclination toward anticipating or experiencing pleasure.

Symptom Checklist-90-revised (SCL-90-R)

The SCL-90-R comprises 90 items assessing psychological problems and symptoms of psychopathology experienced within the past seven days. It contains nine syndrome scales, including somatization, obsessive-compulsive, interpersonal sensitivity, depression, anxiety, hostility, phobic anxiety, paranoid ideation, psychoticism. The total of these subscales assesses the general severity index (range: 0-360) [17]. Each item reflects distinct symptoms and is rated on a 5-point Likert scale (0 = not at all, 4 = very much), with higher scores indicating stronger symptom severity. The reported values represent the sum score of each subscale divided by the number of items within that scale.

Altered State of Consciousness (5D-ASC)

Subjective effects of psilocybin vs placebo were captured using the 5D-ASC [18]. This questionnaire comprises 94 items across 5 key dimensions and 11 subscales of altered states of consciousness. Participants completed a short version approximately 3 hours after drug administration and a full version when subjective effects subsided, approximately 7 hours later. The 5 dimensions include oceanic boundlessness, anxious ego dissolution, visionary

restructuralization, auditory alterations, and reduction in vigilance. The corresponding total score was calculated using the mean of three dimensions oceanic boundlessness, dread of ego dissolution, and visionary restructuralization. Furthermore, the 11 subscales are comprised of elementary imagery, disembodiment, impaired control and cognition, anxiety, blissful state, experience of unity, spiritual experience, insightfulness, changed meaning of percepts, complex imagery, and audio-visual synesthesia (range: 0-100).

Statistical Analysis
Acute subjective effects were explored between the psilocybin and placebo groups utilizing a two-way ANOVA (group x subscale) for the 5 dimensions and 11 subscales of 5D-ASC. To explore the relationship between the subjective experience (11 subscales of 5D-ASC) and follow-up alcohol use characteristics, a correlation matrix was constructed using Spearman correlation coefficients including participants in the psilocybin group (n = 18 for 4-week follow-up, n = 17 for 6-month follow-up). All analyses were conducted using R statistical software, with the following packages: dplyr, tidyverse, rstatix, ggplot, ggpubr, effsize, ggradar, reshape, psych, survival, lme4, lmertest, sjstats, jtools, sjPlot, sjstats, lattice, and gridExtra. Statistical significance was established at $p < 0.05$, two-tailed.

Missing Data
Two participants withdrew from the study after the 4-week follow-up, and one participant in the placebo group was unable to attend the 2-week follow-up in person and did not complete the questionnaires at the 6-month follow-up but provided data of the timeline followback. Furthermore, one participant in the placebo group returned the timeline followback questionnaire for the 6-month follow-up, but not other questionnaires, while one participant in the psilocybin group only returned the timeline followback questionnaire for 161 days following substance visit (instead of 180 days) but filled in the questionnaires at 6-month follow-up.

All randomized participants with at least one efficacy assessment 28 days post-treatment were included in the analysis. To address missing data points and account for non-normal data distribution, we utilized random coefficient models to evaluate group differences in clinical scores over time. Specific sample sizes at each timepoint are reported in the corresponding Supplementary Figures S4-13.

Protocol Deviations
Study timeline: Several visits were delayed due to illness (for a few days), which are accounted for in the reported mean days between study visits. In addition, one participant in the psilocybin group had completed their prior withdrawal treatment 7 weeks, rather than the required 6 weeks, before study inclusion.

BMI: Two participants had a BMI higher than our inclusion criteria of 30 kg/m^2 (one participant with 35.8 kg/m^2 in the psilocybin group and one participant with 32 kg/m^2 in the placebo group).

Previous psychedelic use: Three participants reported a higher use of psychedelics in the last 10 years than the allowed 10 instances (two in the psilocybin group and one in the placebo group, as reported in Table 1).

Illicit drug use within 4-week follow-up: Following drug administration, some participants in the psilocybin group used illicit drugs during the study period. In the placebo group, one participant used LSD and cocaine between the 2- and 4-week follow-up, another participant used LSD within this timeframe, and one participant used cannabis between the 1-day and 4-week follow-up. In the psilocybin group, one participant used cannabis between the 1-day and 4-week follow-up, and another participant used MDMA and cocaine between the 2- and 4-week follow-up.

Prescription drug use between withdrawal treatment and substance visit: In the placebo group, one participant used Naltrexone (50 mg/day), and another used Methylphenidate until study enrollment. One participant took Lisdexamfetamine (20 mg/day) and Zolpidem (2.5 mg/day) until study enrollment, while another used Nalmefene until study enrollment. Additionally, one participant in the placebo group took Lorazepam (10 mg) once between the screening and preparation visits. In the psilocybin group, one participant used Lisdexamfetamine (50 mg/day) until the screening visit, and another took Trazodone (50 mg –150 mg/day) until study enrollment.

Prescription drug use within 4-week follow-up: One participant in the placebo group resumed taking Naltrexone (50 mg/day) to manage alcohol cravings but stopped 3 days before the study visits at the 2- and 4-week follow-up to avoid affecting the neuroimaging measurement. Another participant used Oxazepam (15 mg/day) for 6 consecutive days between the 2- and 4-week follow-up (SAE: inpatient withdrawal treatment). One participant resumed taking Lisdexamfetamine after the 1-day follow-up (20 mg, 3x/week). In the psilocybin group, two

participants used Lisdexamfetamine once between the 2- and 4-week follow-up (1x20 mg). All medication and drug use occurred at least 3 days before the study visits.

Illicit drug use within 3- and 6-month follow-up period

During the 3- and 6-month follow-up, three participants in each group used MDMA. Three participants in the placebo group and one in the psilocybin group used psilocybin, with one participant in each group using psilocybin to microdose. Four participants in the placebo group and one in the psilocybin group used LSD (two microdosing in the placebo group, one microdosing in the psilocybin group). One participant in the placebo group used 2CB, and one in the psilocybin group Ketamine. Lastly, four participants in the placebo and five in the psilocybin group used cocaine or amphetamine in the follow-up period.

Psychoactive drug use within 3- and 6-month follow-up period

Regarding prescribed drugs, three participants in the placebo group resumed taking antidepressants (fluoxetine, duloxetine, and agomelatine). One of these participants also took additional ADHD medication. Another participant in the placebo group started Disulfiram (400 mg, 3x/week) within the 3-month follow-up period, and one other participant took Lorazepam. In the psilocybin group, one participant resumed Bupropion and Lisdexamfetamine during the 3-month follow-up period, and two participants took mood stabilizers/antidepressants (Valproate at the 3-month follow-up, Venlafaxine, and Bupropion at the 6-month follow-up).

Additional therapeutic support

One participant in the placebo group required additional psychological support, which was provided through a 30-minute phone call between the 1-day and 2-week follow-up. In total, 10 out of 18 participants in the placebo group were receiving therapy at the 3-month follow-up, and 8 out of 17 at the 6-month follow-up. In the psilocybin group, 9 out of 17 participants were in therapy at the 3-month follow-up, and 6 out of 17 at the 6-month follow-up. Therapy included CBT, psychoanalysis, couples therapy, and other therapeutic approaches.

Results

The proportional hazard assumption for the 4-week follow-up is borderline but generally met (both group and global: $\chi2 = 3.43$, p = 0.064), suggesting caution in interpreting the results. Visual inspection of Schoenfeld residuals plots showed no significant time-dependent patterns, further supporting the assumption's validity.

No differences in abstinence at 6-month follow-up.

In line with the 4-week follow-up, the Kaplan-Meier survival analysis for the 6-month follow-up period indicated no significant difference in relapse between groups ($p = 0.82$, psilocybin mean = 44.9 days, 95% CI: 29.4 – 60.4; placebo mean = 46.3 days, 95% CI = 30.8 – 61.8, see Fig. 2D). As the proportional hazard assumption was met (both group and global: $\chi2 = 1.18$, $p = 0.28$), the cox regression hazard rate was 0.92 (95% CI = -0.44 – 1.92), indicating that the participants in the psilocybin group had an 8% lower chance of relapse than the placebo group. Exact estimates should be interpreted with caution due to small sample size. In total, 18% of participants in the psilocybin and 16% in the placebo group stayed abstinent within 180 days post-dosing visit. Furthermore, we found no significant difference in duration of abstinence ($p = 0.55$, Cohen's $d = 0.104$) and total abstinent days ($p = 0.45$, Cohen's $d = 0.259$) between the psilocybin and placebo group (see Fig. 2E-F). In sum, we observed no significant difference between the psilocybin and placebo group regarding abstinence at the 4-week and 6-month follow-ups.

The comparison between the psilocybin and placebo groups revealed no significant difference in terms of either the percentage of heavy drinking days or blood alcohol markers.

To compare our findings with prior research by Bogenschutz and colleagues [19], we calculated percentage heavy drinking days per participant (> 4 AU for females and > 5 AU for males) between the dosing visit and the 4-week and 6-month follow-up. Our analysis revealed no significant difference between the psilocybin and placebo group at 4-week follow-up ($p = 0.40$, Cohen's $d = 0.104$; Fig. 3E) or 6-month follow-up ($p = 0.82$, Cohen's $d = 0.042$; Fig. 3F-G).

In line with self-report data, we observed no significant difference between psilocybin and placebo in levels of blood markers of alcohol consumption (ASAT/GOT, ALAT/GPT, and Gamma-GT) at screening and 4-week follow-up or within the groups between the timepoints (Fig. 3H-J). The ASAT/GOT values were missing in two participants in the placebo group for the 4-week follow-up. Those were excluded from the analysis, leading to a total sample size of n = 35. For the ALAT/GPT and Gamma-GT, data was missing for one participant, leading to a sample size of n = 36. We observed no significant difference between the screening and 4-week follow-up levels of ASAT/GOT in either the psilocybin group (n = 18, 95% CI = -2.51 – 3, $p = 0.94$) nor the placebo group (n = 17, 95% CI = -2 – 5, $p = 0.20$, Fig. 3H). Comparing the groups revealed no significant difference at screening (psilocybin: n = 18, placebo: n = 17, 95% CI = -58.82 – 59.95, $p = 0.50$) or 4-week follow-up (95% CI = -58.86 – 65.91, $p = 0.79$). ALAT/GPT levels showed no significant change for the psilocybin group (n = 18, 95% CI =-2 – 5.5, $p = 0.25$) or the placebo group (n = 18, 95% CI = -3 – 7, $p = 0.27$, Fig. 3I). Furthermore, we observed no significant difference in ALAT/GPT levels between the groups at screening (n = 36, 95% CI = -61.41 – 62.49, $p = 0.54$) or 4-week follow-up (95% CI = -61.47 – 62.43, $p = 0.48$). Additionally, no significant change in Gamma-GT levels was detected for both the psilocybin group (n = 18, 95% CI = -1 – 4, $p = 0.10$) and the placebo group (n = 18, 95% CI = -3 – 4.5, $p = 0.41$, Fig. 3J). This suggests that there was no significant alteration in ASAT, ALAT, or Gamma-GT levels following the intervention. Regarding the group comparison (psilocybin vs placebo), we found no significant difference at screening (n = 36, 95% CI = -61.49 – 62.4, $p = 0.66$) or 4-week follow-up (95% CI = -61.5 – 62.37, $p = 0.40$).

Intention-to-Treat (ITT) Approach reveals no substantial differences at primary efficacy timepoints.

We repeated the primary outcome analysis at 4-week follow-up including all randomized participants using an ITT approach.

For craving (PACS) and alcohol self-efficacy (AASE) analyses, two additional participants with data at the 1-day follow-up were included, and one participant with 1-day and 2-week follow-up data, increasing the total sample to n = 40 (psilocybin: n = 19, placebo: n = 21). Consistent with the main analysis, there was a significant decrease in craving scores at the 1-day follow-up ($\beta = -4.18$, 95% CI = -6.35 – -2.00, $p = 0.00019$), observed across both groups. Additionally, there was a further significant decrease in craving within the psilocybin group ($\beta = -5.23$, 95% CI = -8.37 – -2.08, $p = 0.0012$). Model details are provided in Supplementary Table S1.

For the AASE temptation score, the analysis revealed a significant decrease in temptation following the dosing visit compared to pre-dosing, across both groups (β = -6.82, 95% CI = -10.21 – -3.43, $p < 0.0001$), with an additional decrease in the psilocybin group (β = -6.99, 95% CI = -11.89 – -2.10, $p = 0.0053$). Regarding the AASE confidence score, a significant increase in confidence was observed in the psilocybin group compared to the placebo group and baseline (β = 7.18, 95% CI = 0.43 – 13.94, $p = 0.037$), which did not reach significance in the per-protocol analysis. Detailed model results are reported in Supplementary Table S2.

No significant change in anhedonia or symptom clusters due to study participation in both groups.

Our data revealed no significant change regarding anhedonia (SHAPS and TEPS) or symptom clusters (SCL-90-R) in the psilocybin or placebo group throughout the study. Therefore, the null models are reported for SHAPS (Supplementary Table S8) and TEPS (Supplementary Table S9). Mean scores are shown in Supplementary Figures S11-12. Comparisons of mean scores in SCL-90-R subscales between baseline and 4-week follow-up are reported in the Supplementary Table S10.

Acute subjective effects and relation to follow-up alcohol use characteristics.

A two-way ANOVA (group x subscale) was conducted on the 5D-ASC questionnaire, more specifically on its 5 domains and 11 subscales. Regarding the 5 domains, we found a significant main effect of group ($\eta^2 = 0.432$, $p < 0.0001$), domain ($\eta^2 = 0.111$, $p < 0.0001$), and their interaction ($\eta^2 = 0.031$, $p = 0.012$). Post-hoc Tukey-tests revealed a significant difference on all domains and the global score (Supplementary Fig. 13A). Likewise, data regarding the 11 subscales revealed a significant main effect of group ($\eta^2 = 0.368$, $p < 0.0001$) and subscale ($\eta^2 = 0.105$, $p < 0.0001$) and a significant interaction between group and subscale ($\eta^2 = 0.034$, $p = 0.0043$). A post-hoc Tukey-test revealed significant differences for all subscales between psilocybin and placebo except anxiety and impaired control and cognition ($p < 0.005$, Supplementary Fig. 13B). In previous studies, the acute subjective effects were associated with clinical outcome measures [20–23]. Therefore, we examined the relationship between acute subjective effects and follow-up alcohol use characteristics in the psilocybin group. Feelings of disembodiment, impaired control and cognition, and complex imagery were associated with alcohol use characteristics. Specifically, feelings of disembodiment were negatively correlated with mean alcohol use at the 4-week follow-up ($r_s = -0.5$, $p = 0.033$, Supplementary Fig. 13C), suggesting that acutely increased feelings of disembodiment were associated with decreased mean alcohol use in the 4 weeks following psilocybin administration. Additionally, impaired control and cognition were positively correlated with mean alcohol use at the 6-month follow-up ($r_s = 0.49$, $p = 0.046$, Supplementary Fig. 13C), indicating that participants acutely experiencing impaired control and cognition exhibited higher alcohol use in the 6-month follow-up period. Lastly, complex imagery was negatively correlated with duration of abstinence at the 6-month follow-up ($r_s = -0.49$, $p = 0.046$, Supplementary Fig. 13C) suggesting that participants acutely experiencing higher levels of complex imagery had a shorter duration of abstinence. However, it is important to note that due to the exploratory nature of this analysis, *p*-values were not corrected for multiple comparisons and should be interpreted with caution.

Subgroup analysis: prior psychedelic use and primary outcome 4-week follow-up.

As half of the participants reported previous psychedelic use, we exploratively examined the potential influence of previous psychedelic use on the primary outcome measures. We observed significant differences in the psilocybin group in abstinence and mean alcohol use at the 4-week follow-up between participants in the psilocybin group with and without prior psychedelic use. Participants with no prior psychedelic experience (n = 8) showed a longer duration of abstinence and lower mean alcohol use at 4-week follow-up than the ones with prior psychedelic use (n = 10; duration of abstinence: $p = 0.00057$, Cohen's $d = 0.823$; total days of abstinence: $p = 0.00078$, Cohen's $d = 0.803$, mean alcohol use: $p = 0.0039$, Cohen's $d = 0.690$; Supplementary Fig. S14).

When focusing exclusively on participants with no prior psychedelic use, we found significant differences between the psilocybin and placebo groups regarding the primary efficacy endpoints (psilocybin: n = 8, placebo: n = 10). Participants receiving psilocybin exhibited longer abstinence duration ($p = 0.0060$, Cohen's $d = 0.659$), more total abstinent days ($p = 0.0069$, Cohen's $d = 0.648$), and significantly lower mean alcohol use ($p = 0.0052$, Cohen's $d = 0.669$; Supplementary Fig. S14), compared to those receiving placebo. All reported *p*-values in this section are uncorrected.

These findings suggest that participants with AUD who have not previously used psychedelics may experience short-term benefits from psychedelic-assisted therapy following withdrawal treatment. However, given the small sample size in this subgroup analysis, these results should be interpreted with caution.

Subgroup analysis: prior psychedelic use and primary outcome 6-month follow-up.

In line, at the 6-month follow-up, participants in the psilocybin group with no prior psychedelic use showed a significantly longer duration of abstinence (p = 0.00060, Cohen's d = 0.844) and higher amount of total abstinent days (p = 0.038, Cohen's d = 0.515) compared to those with previous use, although differences in mean alcohol use were not statistically significant (mean alcohol use: p = 0.074, Cohen's d = 0.443). These findings suggest that prior psychedelic use may particularly influence short-term alcohol use characteristics.

Among participants with no prior psychedelic use, those receiving psilocybin had a longer duration of abstinence compared to the placebo group (p = 0.012, Cohen's d = 0.601) at the 6-month follow-up. However, no significant differences were found between the groups regarding total abstinent days (p = 0.153, Cohen's d = 0.348) or mean alcohol use (p = 0.25, Cohen's d = 0.283, p-values uncorrected). However, given the small sample size, findings should be interpreted with caution.

Inclusion and exclusion criteria

Participants fulfilling all the following inclusion criteria were eligible for the study:

- Informed Consent as documented by signature
- Male and female in- and outpatients 18 years to 60 years of age
- Right-handedness according to Oldfield (1971) performed during the telephone screening, laterality index ≥ 0.2
- DSM-IV-diagnosis of alcohol use disorder (based on clinical assessment and confirmed by the SCID Interview)
- Having undergone withdrawal treatment from alcohol use or have stopped using alcohol within 6 weeks prior to enrolment in the study
- Drug free from any psychotropic and serotonergic medication for at least five days before administration of the study drug or placebo
- No alcohol use between withdrawal treatment and administration of study drug or placebo
- Good physical health with no unstable medical conditions, as determined by medical history, physical examination, routine blood labs, electrocardiogram, urine analysis, and urine toxicology
- Normal level of language comprehension (German or Swiss-German)
- Willing to refrain from drinking caffeinated drinks during the testing days and from consuming psychoactive substances after enrolling in the study until visit 6
- Women of childbearing potential must be using an effective, established method of contraception for the entire study duration, such as oral, injectable, or implantable contraceptives, or intrauterine contraceptive devices. Note: female participants who are surgically sterilized / hysterectomized or post-menopausal for longer than 2 years are not considered as being of childbearing potential.
- Have a family member or friend who can pick them up and stay with them overnight after the psilocybin administration sessions (driving is forbidden at drug treatment days)
- No other medication than reported at study inclusion is allowed until visit 6, except for emergencies

The presence of any one of the following exclusion criteria lead to exclusion of the participant:

- Allergy, hypersensitivity, or other adverse reaction to previous use of psilocybin or other hallucinogens
- Uncorrected hypertension (assessed at screening day: higher than 139 systolic and 89 diastolic)
- Women who are pregnant or breast feeding
- Intention to become pregnant during the course of the study
- Lack of safe contraception, defined as: Female participants of childbearing potential, not using and not willing to continue using a medically reliable method of contraception for the entire study duration, such as oral, injectable, or implantable contraceptives, or intrauterine contraceptive devices, or who are not using any other method considered sufficiently reliable by the investigator in individual cases (Female participants who are surgically sterilized / hysterectomized or post-menopausal for longer than 2 years are not considered as being of child bearing potential)
- Known or suspected non-compliance
- Inability to follow the procedures of the study, e.g. due to language problems, psychological disorders, dementia, etc. of the participant
- Previous enrolment into the current study
- Enrolment of the investigator, his/her family members, employees and other dependent persons
- Lifetime history of bipolar disorder (I, II, not otherwise specified) based on our clinical interview at screening visit
- Lifetime history of schizophrenia, schizoaffective disorder, or psychosis not otherwise specified based on our clinical interview at screening visit, expect psychotic symptoms under acute alcohol intoxication or during withdrawal treatment
- History of DSM-IV drug dependence other than alcohol (except for caffeine or nicotine) within two months prior to enrolment
- Comorbid Axis I anxiety and depression disorders diagnoses as well as post-traumatic stress disorder and ADHD will be permitted if they do not require treatment after inclusion in the study
- Family history of schizophrenia or schizoaffective disorder, or bipolar disorder type 1 (first- or second-degree relatives)

- Violent behavior within last 2 years or history of suicidal behavior
- Lifetime history of hallucinogen use on more than 10 occasions within last 10 years
- Getting psychotherapeutic or psychological treatment from third parties during the study until visit 6 is forbidden
- Abnormal electrocardiogram
- Any unstable illness as determined by history or laboratory tests
- BMI <17 or >30
- Uncorrected hypo- or hyperthyroidism
- Contraindications to magnetic resonance imaging (MRI safety form)
- During the study, new use or dose changes of already existing concomitant medication without prior informing the investigators is forbidden
- High risk of adverse emotional or behavioral reaction based on investigator's clinical evaluation (e.g., evidence of serious personality disorder, antisocial behavior, serious current stressors, lack of meaningful social support)
- Participation in another study with investigational drug within the 30 days preceding and during the present study (until visit 6)
- Taking medications that are known to modulate uridine diphosphate glucuronosyltransferase enzyme
- Inhibitors of UGT1A9 and 1A10 should be discontinued at least five half-lives prior to the administration of psilocybin
- Monoamine oxidase and aldehyde or alcohol dehydrogenase inhibitors should be discontinued at least 5 half-lives prior to the dose of psilocybin

Supplementary Figures

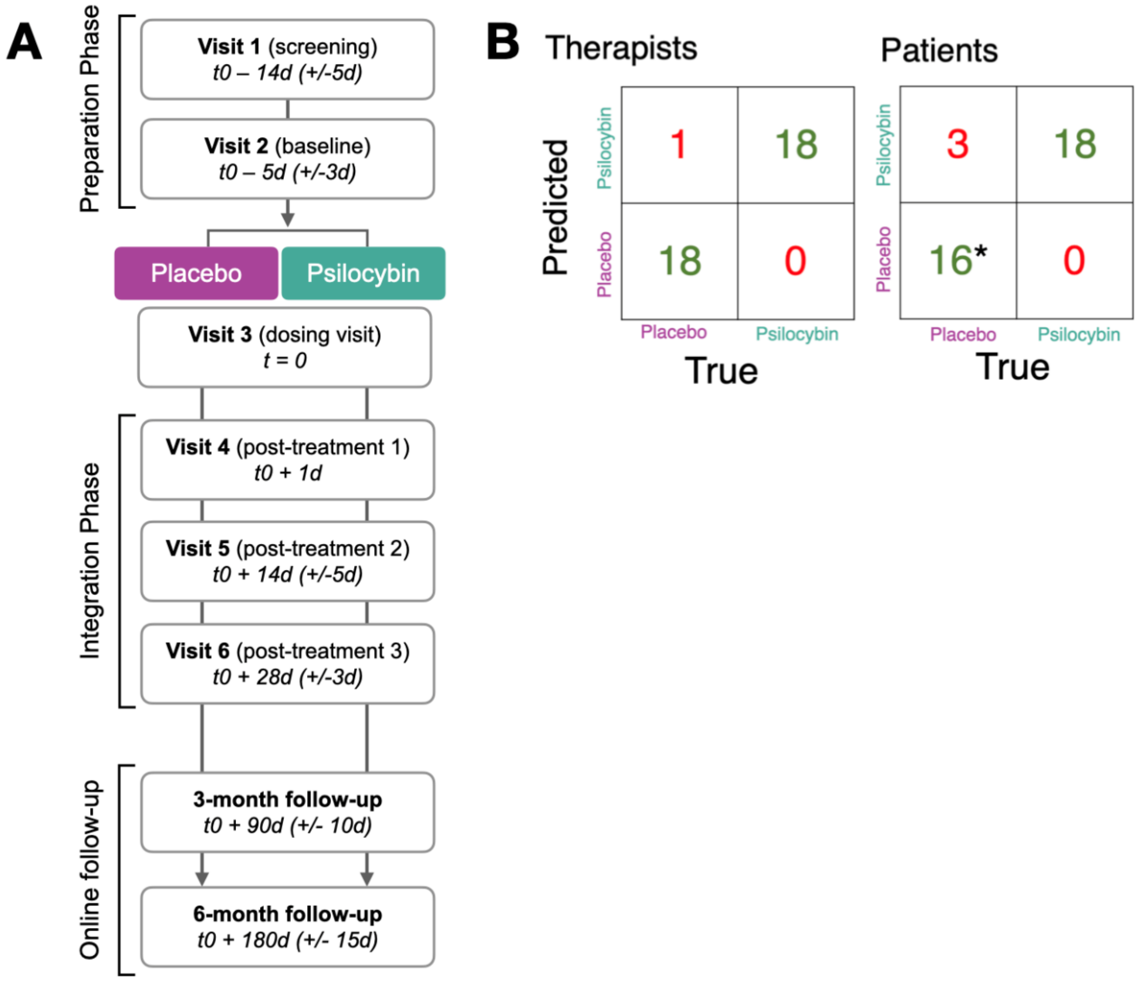

Supplementary Figure S1: A: Detailed study procedure. B: Detailed information on blinding. *: one participant could not decide between psilocybin and placebo and has been included in the predicted placebo group. d=days.

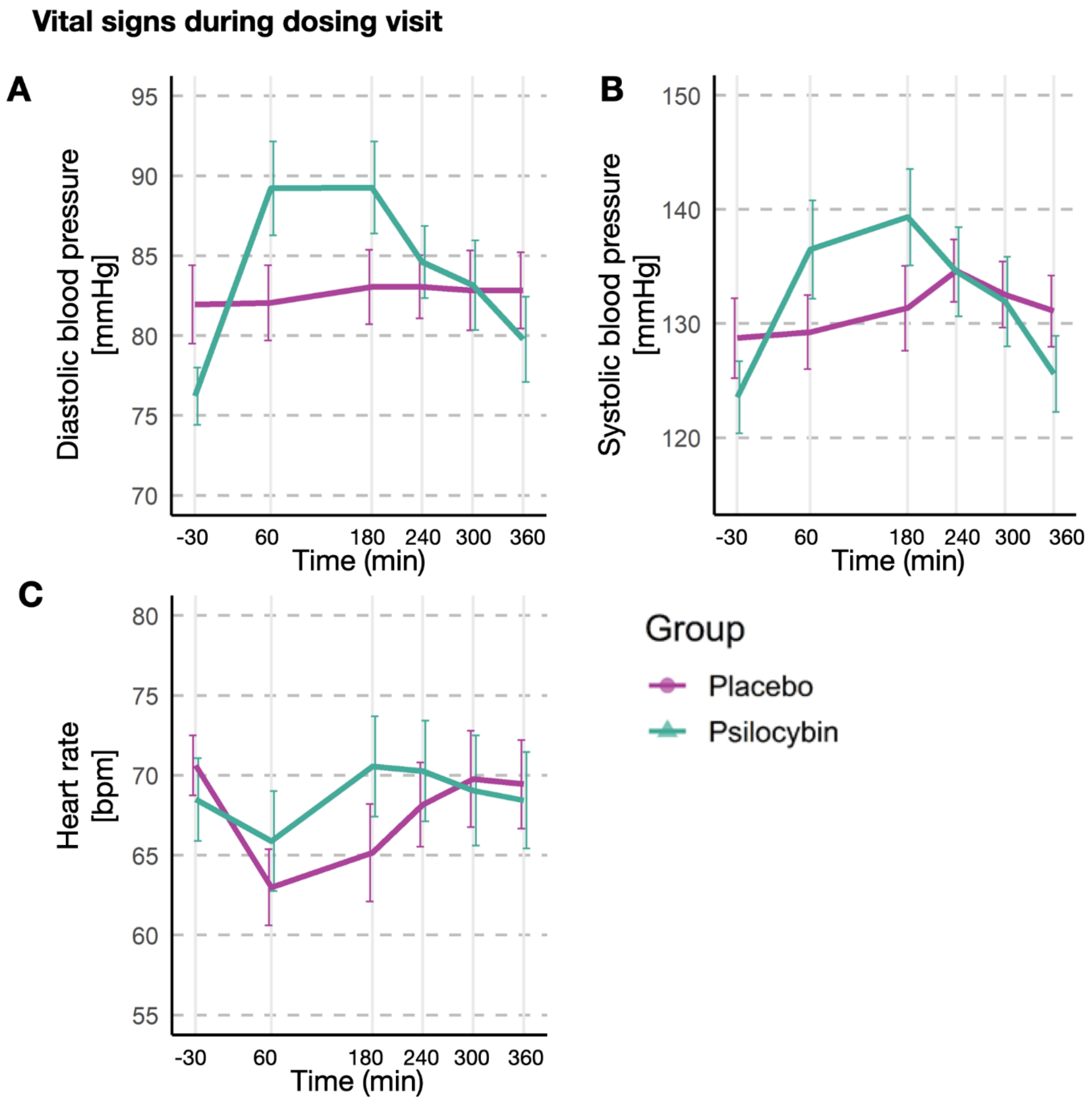

Supplementary Figure S2: Vital signs during dosing visit. Measurement happened approximately at the stated time. No measurement was taken 120 hours post substance administration as patients were in the MRI-scanner. A: Mean ± SEM of diastolic blood pressure [mmHg] throughout the acute drug effect. B: Mean ± SEM of systolic blood pressure [mmHg] throughout the acute drug effect. C: Mean ± SEM of heart rate [beats per minute; bpm] throughout the acute drug effects. (N=37).

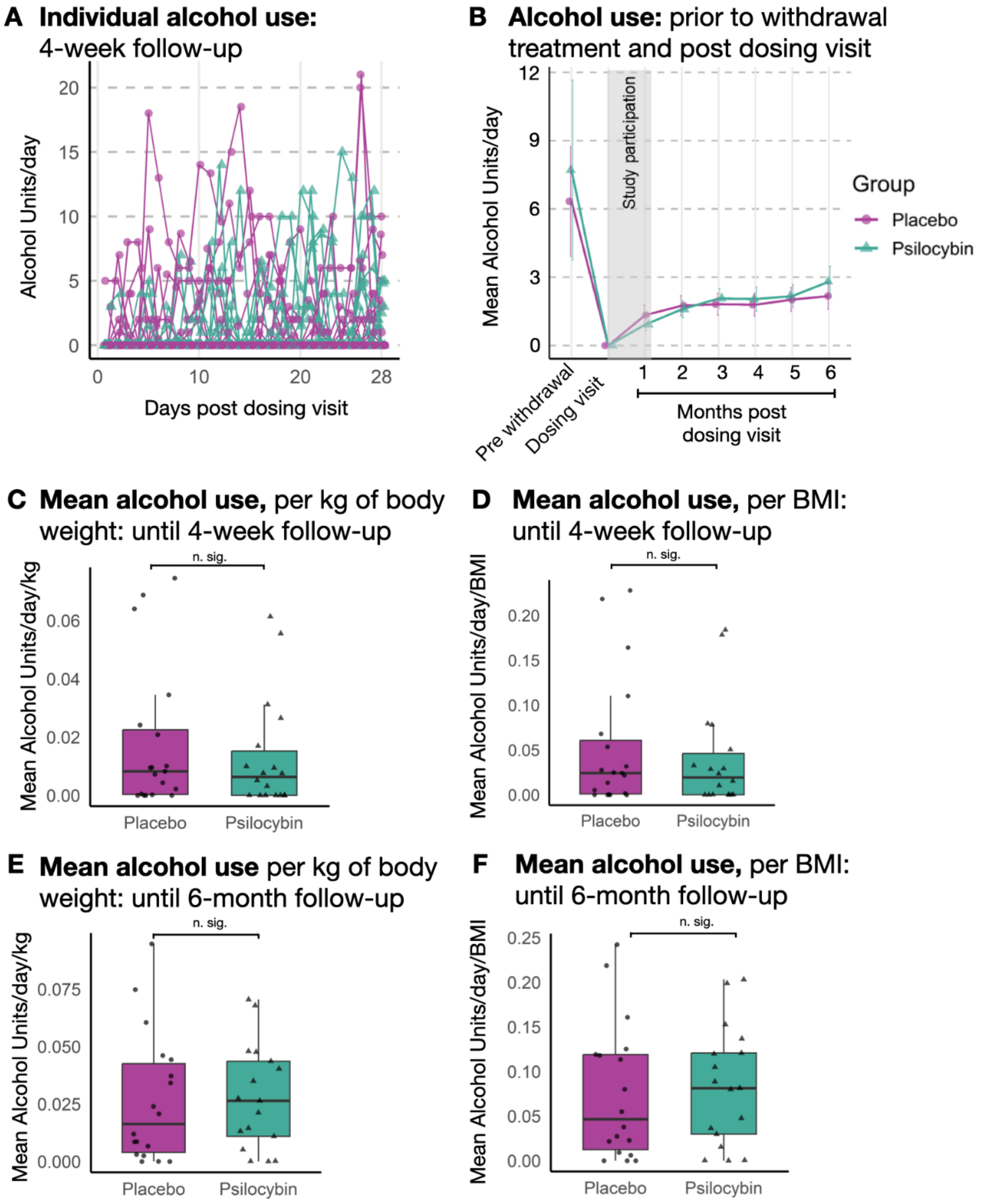

Supplementary Figure S3: Alcohol use characteristics. A: Individual alcohol use (standard alcohol units/day) 28-days following dosing visit. B: Mean ± SEM alcohol use prior to withdrawal treatment and following dosing visit up to 6-month follow-up. Withdrawal treatment was not part of the study procedure. C: Mean alcohol use per kilogram of body weight (standard alcohol units/day/kg) between dosing visit until 4-week follow-up. D: Mean alcohol use per BMI (standard alcohol units/day/BMI) between dosing visit until 4-week follow-up. E: Mean alcohol use per kilogram of body weight (standard alcohol units/day/kg) between dosing visit and 6-month follow-up. F: Mean alcohol use per BMI (standard alcohol units/day/BMI) between dosing visit and 6-month follow-up. 4-week follow-up: N=37, 6-month follow-up: n=35.

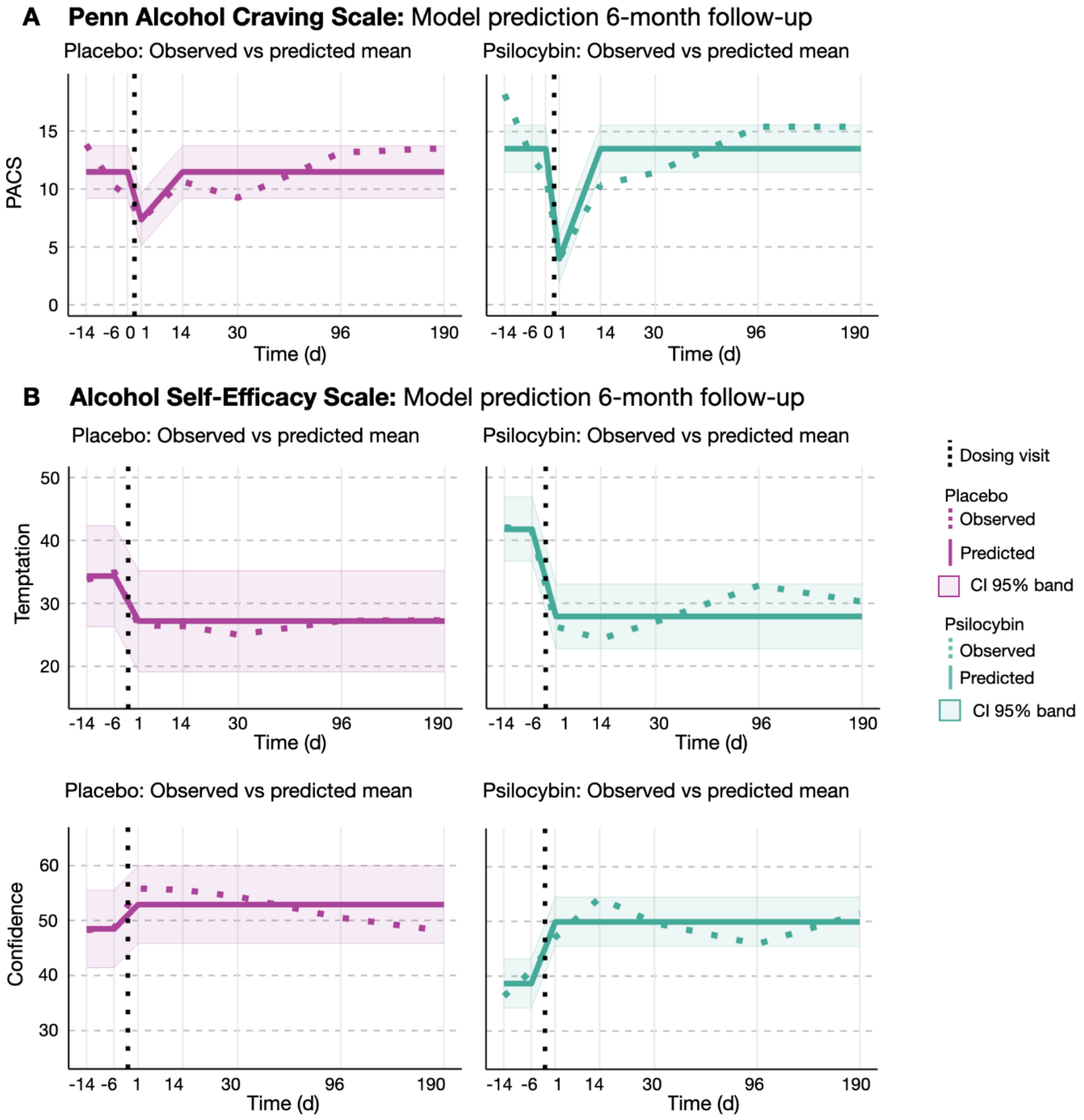

Supplementary Figure S4: Random coefficient model observed vs predicted values of Penn Alcohol Craving Scale (PACS) and Alcohol Self-Efficacy Scale (AASE). Time period between screening and 6-month follow-up. A: N=37 at timepoint -14days, -6days, day 0, +1day, +30days; n=36 at +14days; n=35 at +96days; n=34 at +190days. B: N=37 at timepoint -14days, -6days, +1day, +30days; n=36 at +14days; n=35 at +96days; n=34 at +190days.

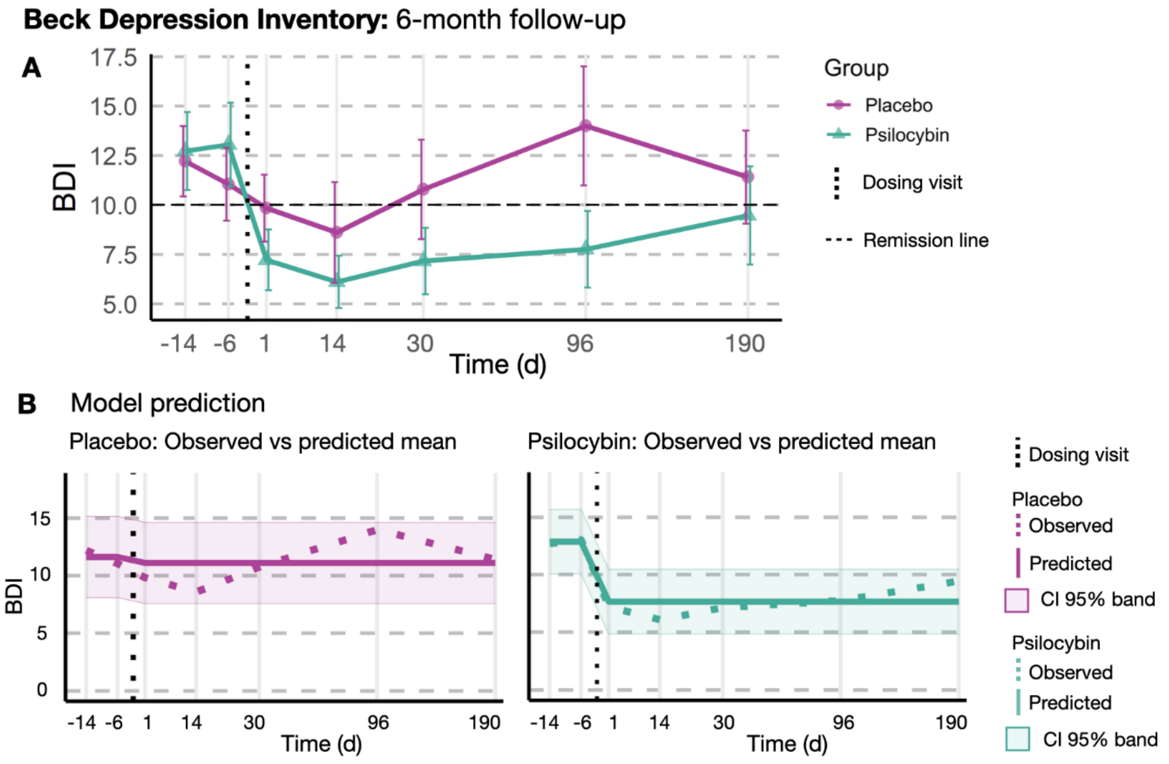

Supplementary Figure S5: Beck's Depression Score (BDI). A: Graph showing mean ± SEM scores. B: Random coefficient model observed vs predicted values of BDI for 6-month follow-up. N=37 at timepoint -14days, -6days, +1day, +30days; n=36 at +14days; n=35 at +96days; n=34 at +190days.

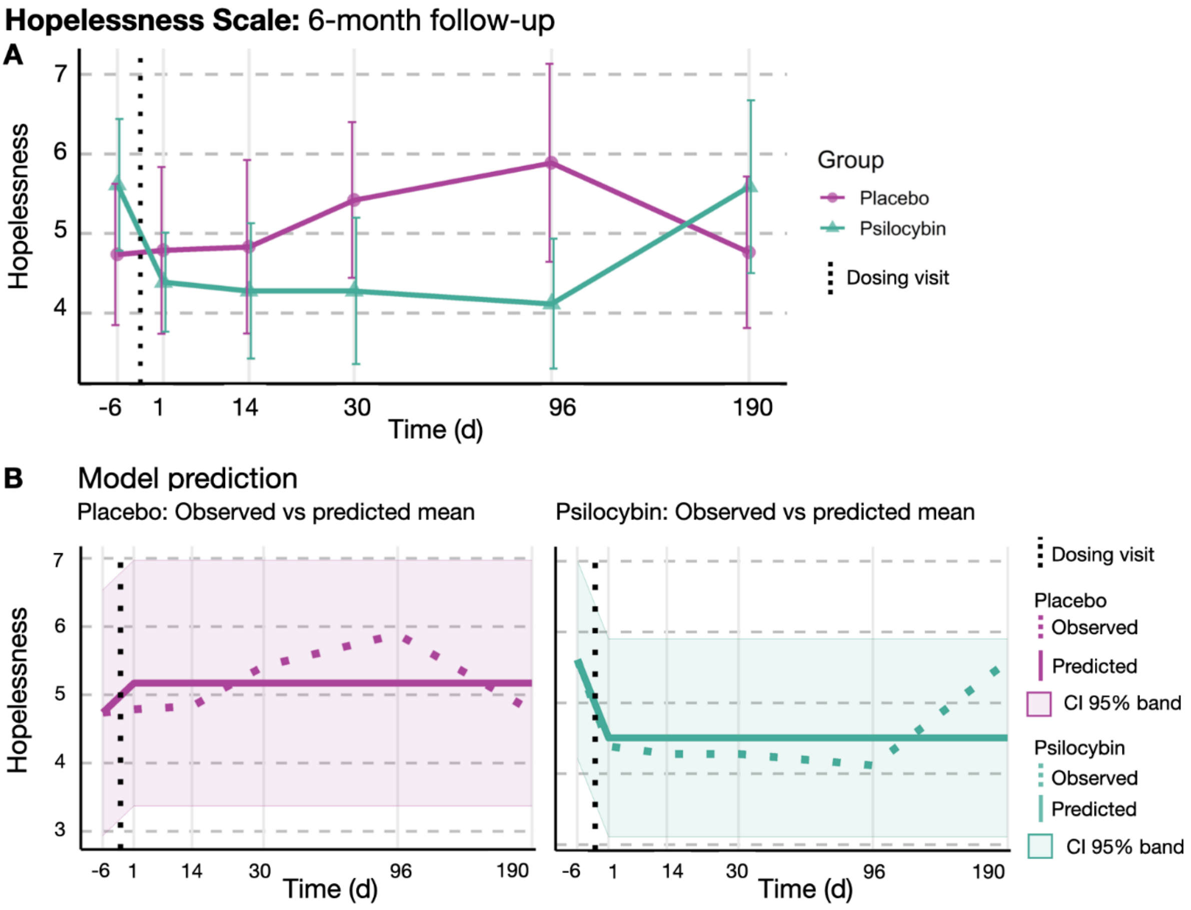

Supplementary Figure S6: Hopelessness Scale: A: Graph showing mean ± SEM scores. B: Random coefficient model observed vs predicted values of Hopelessness for 6-month follow-up. N=37 at timepoint -6days, +1day, +30days; n=36 at +14days; n=35 at +96days; n=34 at +190days.

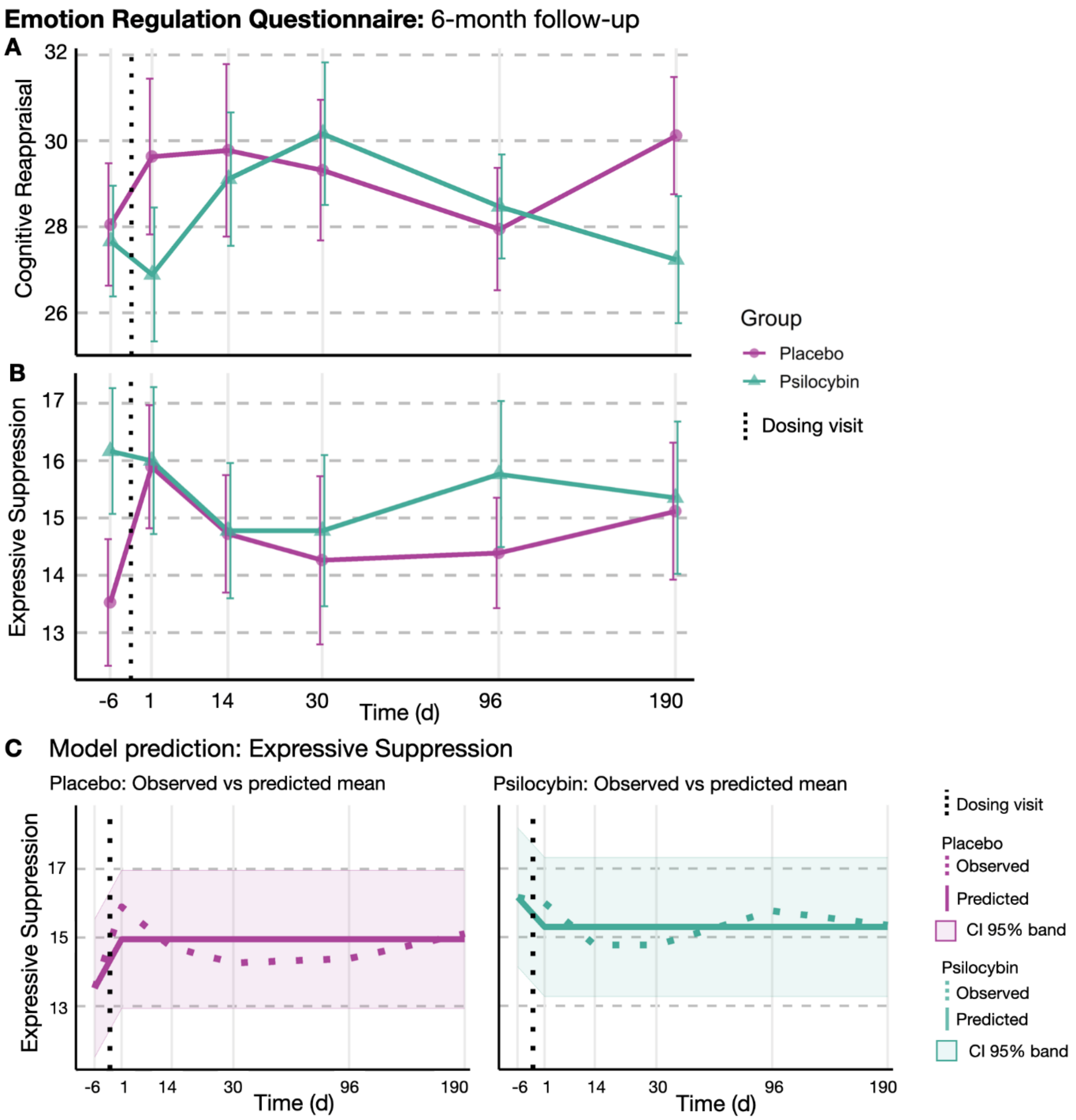

Supplementary Figure S7: Emotion Regulation Questionnaire (ERQ): A-B: Graph showing mean ± SEM scores. C: Random coefficient model observed vs predicted values of Expressive Suppression for 6-month follow-up. The null model was used for the subscale Cognitive Reappraisal and is thus not reported here. N=37 at timepoint -6days, +1day, +30days; n=36 at +14days; n=35 at +96days; n=34 at +190days.

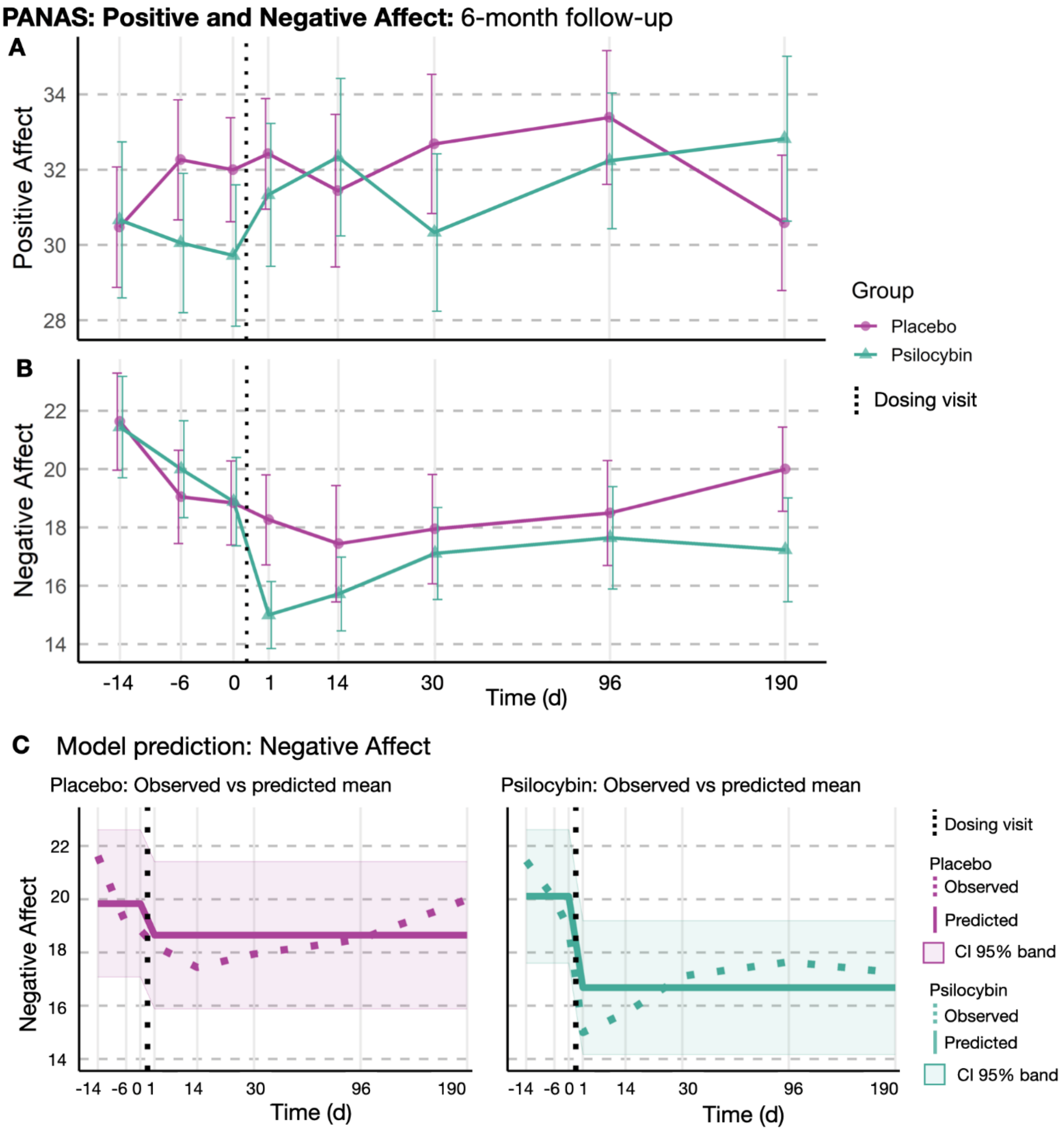

Supplementary Figure S8: PANAS (positive and negative affect schedule). A-B: Graph showing mean ± SEM scores. C: Random coefficient model observed vs predicted values of Negative Affect for 6-month follow-up. The subscale positive affect is not shown here as the null model was used. N=37 at timepoint -14days, -6days, day 0, +1day, +30days; n=36 at +14days; n=35 at +96days; n=34 at +190days.

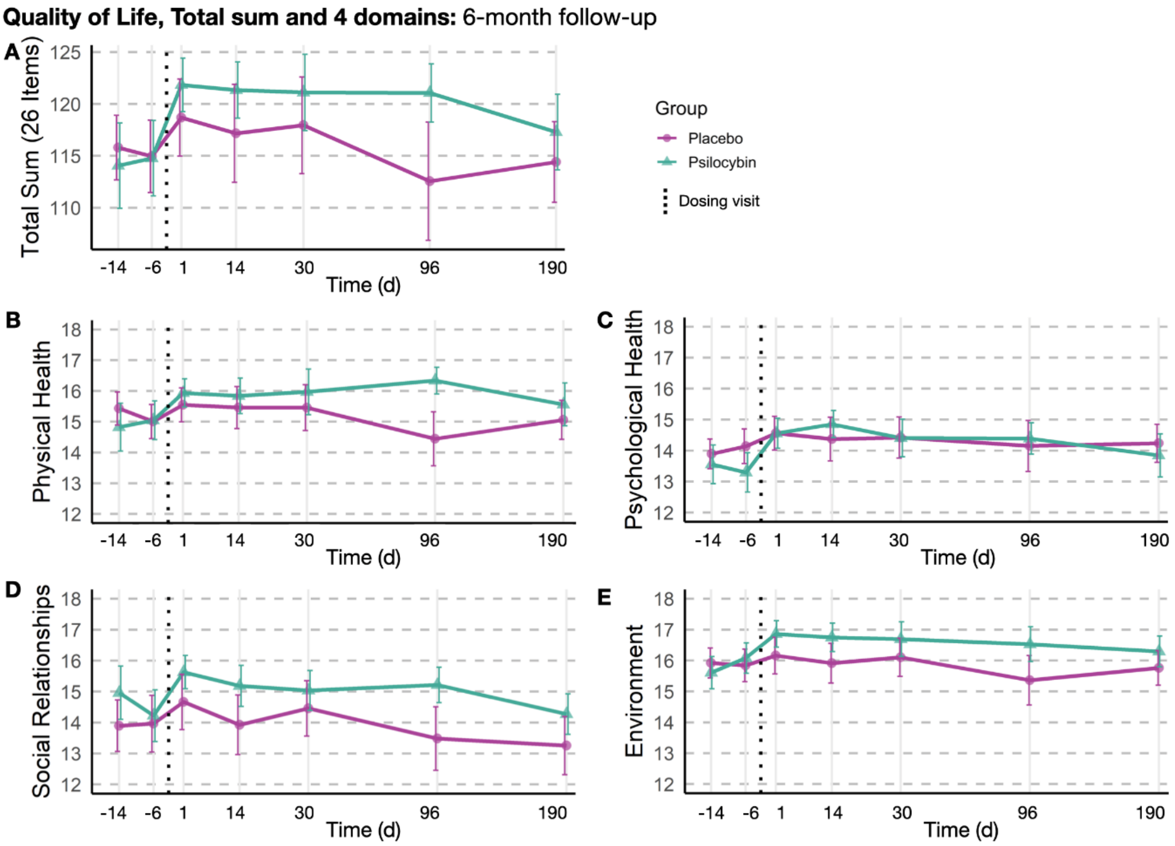

Supplementary Figure S9: Quality of life. A-E: Graph showing mean ± SEM scores. A: 6-month follow-up overall quality of life (sum of 26 items). B: 6-month follow-up quality of life, physical health. C: 6-month follow-up quality of life, environment. N=37 at timepoint -14days, -6days, +1day, +30days; n=36 at +14days; n=35 at +96days; n=34 at +190days.

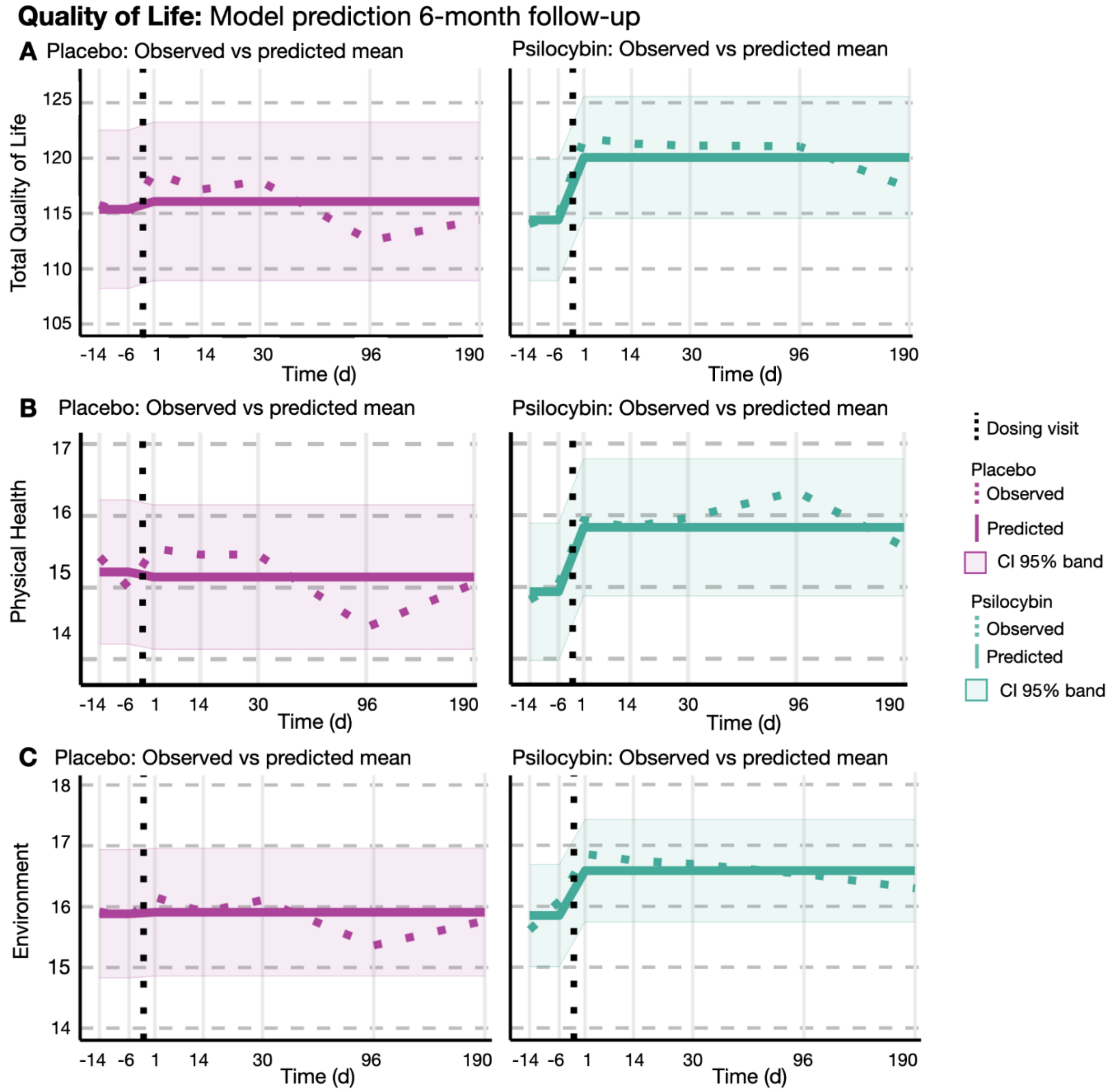

Supplementary Figure S10: Quality of Life. Random coefficient model: model prediction vs observed values of quality of life. A: 6-month follow-up overall quality of life (sum of 26 items). B: 6-month follow-up quality of life, physical health. C: 6-month follow-up quality of life, environment. N=37 at timepoint -14days, -6days, +1day, +30days; n=36 at +14days; n=35 at +96days; n=34 at +190days.

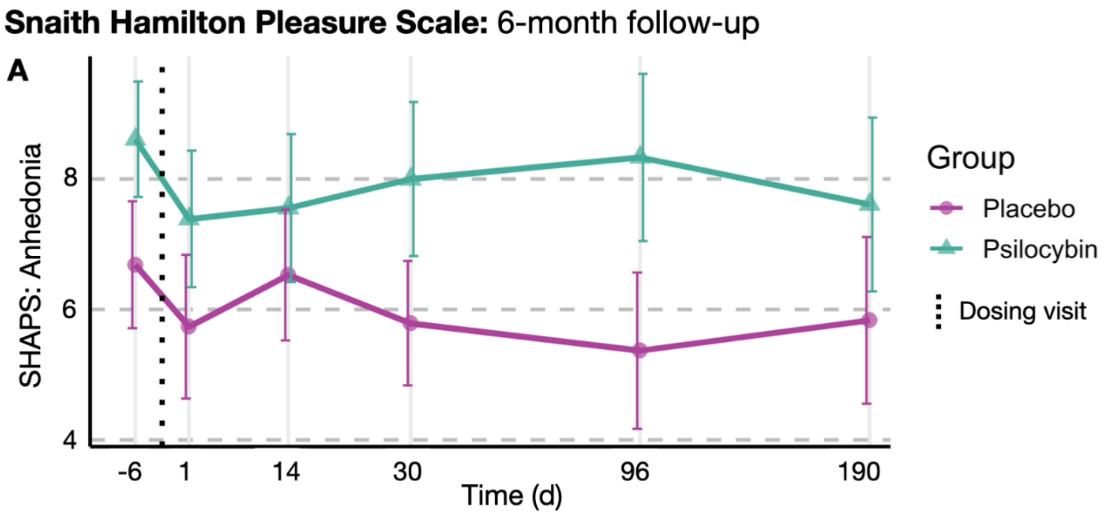

Supplementary Figure S11: Snaith Hamilton Pleasure Scale. A: Graph showing mean ± SEM scores. As we chose the null model, no prediction graph is presented here. N=37 at timepoint -6days, +1day, +30days; n=36 at +14days; n=35 at +96days; n=34 at +190days.

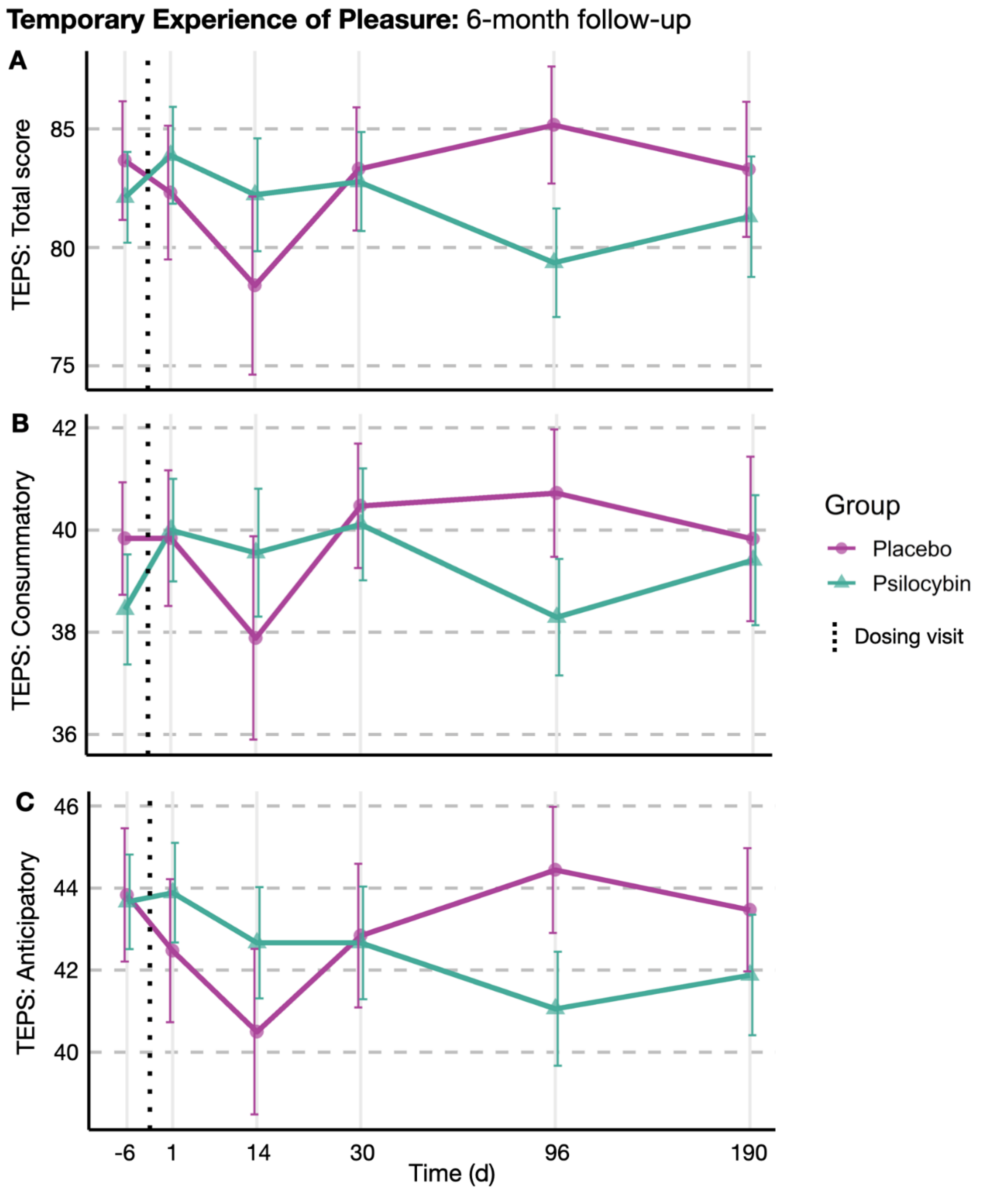

Supplementary Figure S12: Temporary Experience of Pleasure Scale. A-C: Graphs showing mean ± SEM scores. As we chose the null model, no prediction graphs are presented here. A: Total score. B: Subscale 'Consummatory Pleasure'. C: Subscale 'Anticipatory Pleasure'. N=37 at timepoint -6days, +1day, +30days; n=36 at +14days; n=35 at +96days; n=34 at +190days.

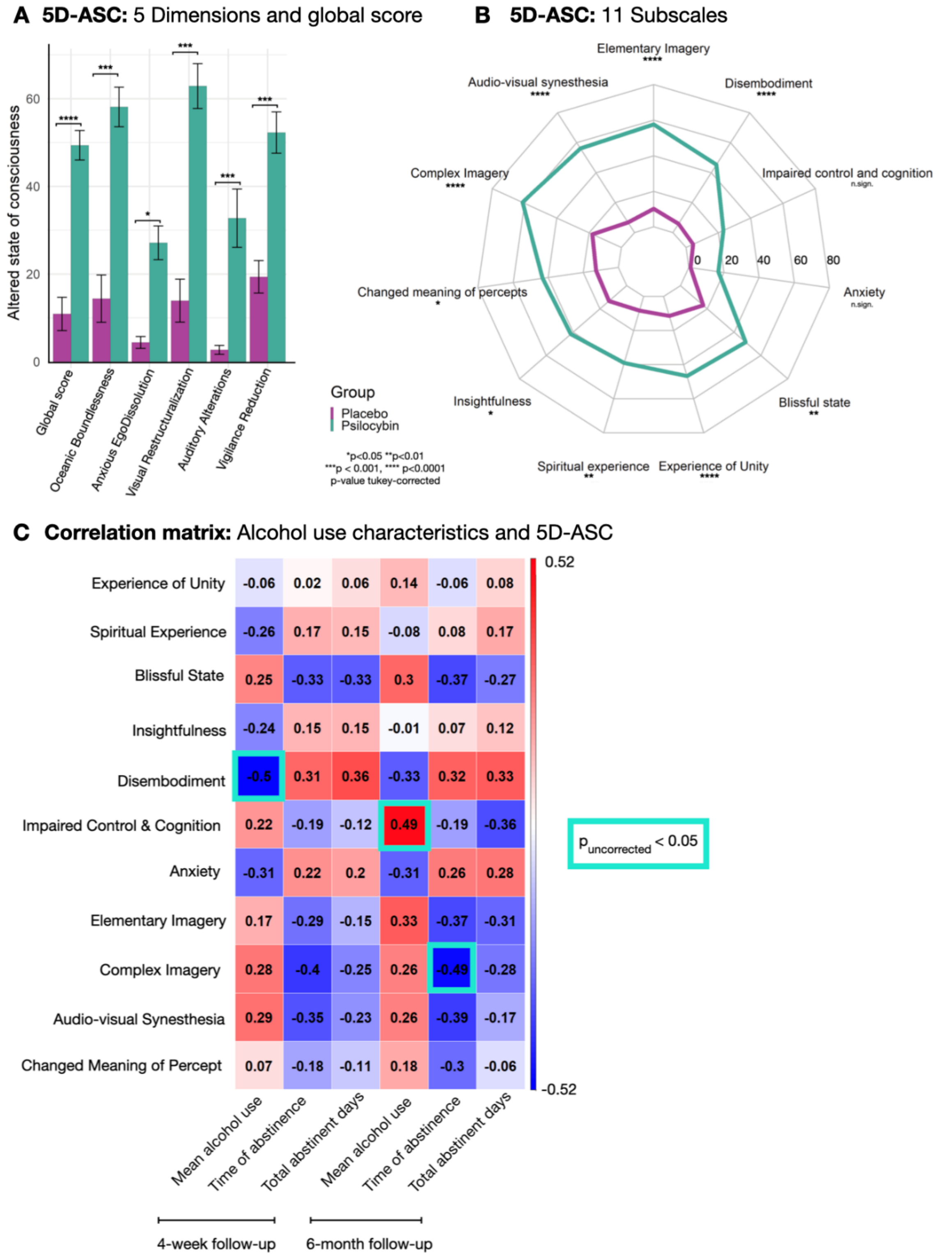

Supplementary Figure S13: Acute subjective effects following psilocybin vs placebo administration (N=37). Experience was assessed using the 5 Dimensions of Altered States of Consciousness (5D-ASC). A: Barplot showing mean ± SEM scores for the global score and 5 subscales. B: Radarplot visualizing subjective effects following psilocybin vs placebo on 11 subscales of the 5D-ASC. C: Correlation matrix displaying Spearman correlation coefficients between acute effects of psilocybin and follow-up alcohol use characteristics. Green squares indicate significance at $p < 0.05$, the analysis is not corrected for multiple comparisons. (n=18 for 4-week follow-up, n=17 for 6-month follow-up).

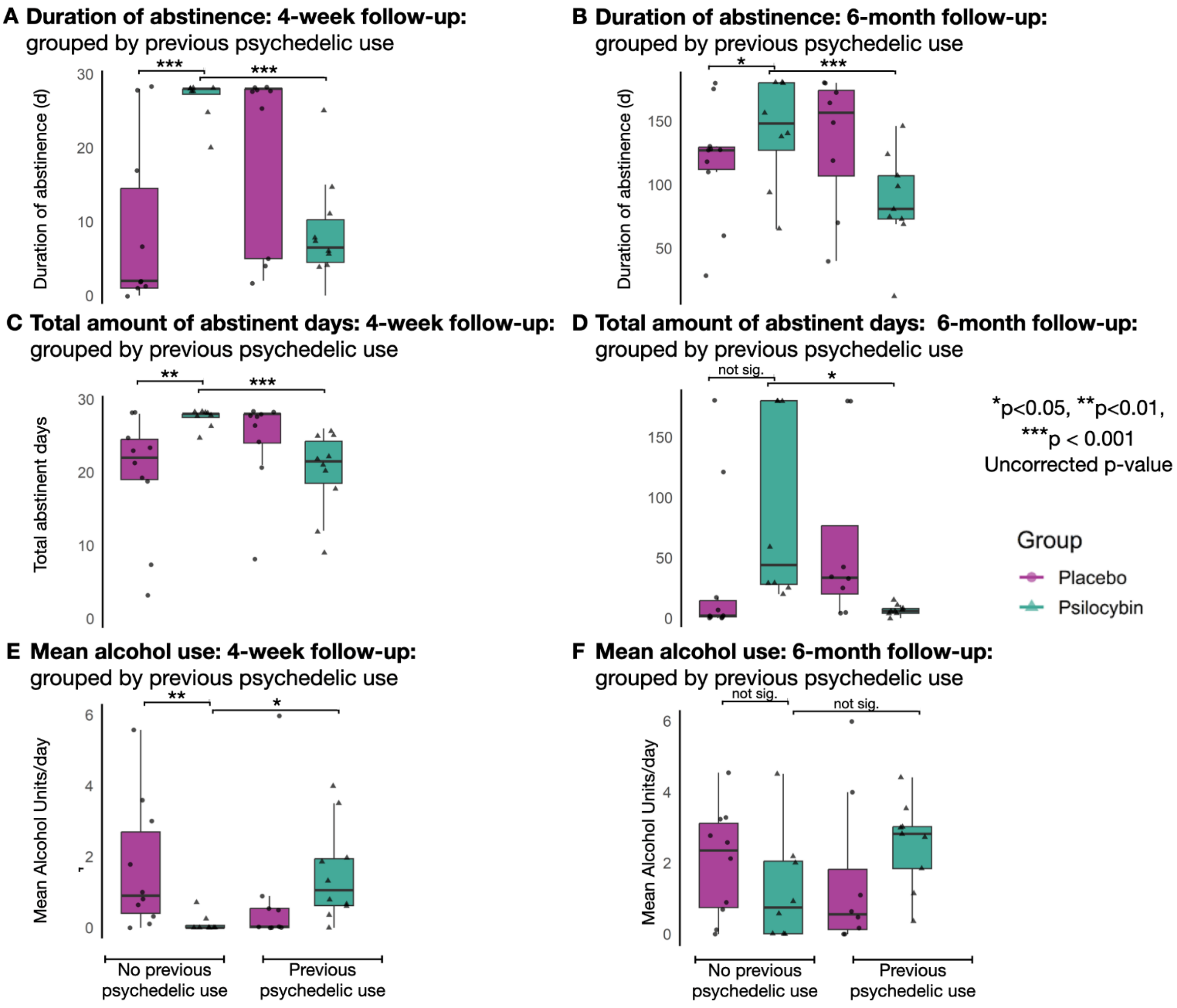

Supplementary Figure S14: Alcohol use characteristics grouped in participants without previous psychedelic use vs with previous psychedelic use. A: Duration of abstinence until 4-week follow-up. B: Duration of abstinence until 6-month follow-up. C: Total amount of abstinent days until 4-week follow-up. D: Total amount of abstinent days until 6-month follow-up. E: Mean alcohol use per day until 4-week follow-up. F: Mean alcohol use per day until 6-month follow-up. 4-week follow-up: N = 37. 6-month follow-up: n = 35. Relapse ≤ 1 SU/day.

Supplementary Tables

Supplementary Table S1: Penn Alcohol Craving Scale 6-month follow-up: Random coefficient model details for per protocol analysis and intention-to-treat approach.

Per protocol analysis

Predictors	*Estimates*	*CI (95%)*	*p*
Intercept	11.48	9.09– 13.88	**<0.001**
Psilocybin (vs Placebo)	2.04	-1.40 – 5.47	0.194
1 day follow-up	-4.12	-6.36 – -1.87	**<0.001**
Psilocybin * 1 day follow-up	-5.40	-8.62 – -2.19	**0.001**
Random Effects			
σ2	21.50		
$\tau_{00\ id}$	24.99		
ICC	0.54		
N	37		
Data points	290		
REML	1785.5		
BIC	1819.5		
Marginal R^2 / Conditional R^2	0.120 / 0.593		

Note: σ2: error variance; ICC: Intraclass correlation coefficient;
τ00: between-person variance
Sample size: N=37 at timepoint -14d, -6d, day 0, +1d, +30d; n=36 at +14d; n=35 at +96d; n=34 at +190d. (d=days)

Intention-to-treat appraoch

Predictors	*Estimates*	*CI (95%)*	*p*
Intercept	11.13	8.88 – 13.38	**<0.001**
Psilocybin (vs Placebo)	2.38	-0.88 – 5.64	0.152
1 day follow-up	-4.18	-6.35 – -2.00	**<0.001**
Psilocybin * 1 day follow-up	-5.23	-8.37 – -2.08	**0.001**
Random Effects			
σ2	22.05		
$\tau_{00\ id}$	23.96		
ICC	0.52		
N	40		
Data points	303		
REML	1873.3		
BIC	1907.5		
Marginal R^2 / Conditional R^2	0.126 / 0.581		

Note: σ2: error variance; ICC: Intraclass correlation coefficient;
τ00: between-person variance
Sample size: N=40 at timepoint -14d, -6d, day 0, +1d; n=37 at +14d, +30d n=35 at +96d; n=34 at +190d. (d=days)

Supplementary Table S2: Alcohol Self-Efficacy Scale 6-month follow-up: Random coefficient model details for per protocol analysis and intention-to-treat approach.

Temptation

**Per protocol analysis**			
Predictors	*Estimates*	*CI (95%)*	*p*
Intercept	34.34	26.81 – 41.87	**<0.001**
Psilocybin (vs Placebo)	7.44	-3.36 – 18.23	0.176
Post measurements	-7.16	-10.66 – -3.67	**<0.001**
Psilocybin * Post measurements	-6.71	-11.71 – -1.70	**0.009**
Random Effects			
σ2	84.12		
$\tau_{00\ id}$	235.69		
ICC	0.74		
N	37		
Data points	253		
REML	1931.5		
BIC	1964.7		
Marginal R^2 / Conditional R^2	0.077 / 0.757		

Note: σ2: error variance; ICC: Intraclass correlation coefficient; τ00: between-person variance
Sample size: N=37 at timepoint -14d, -6d, +1d, +30d; n=36 at +14d; n=35 at +96d; n=34 at +190d. (d=days)

**Intention-to-treat approach**			
Predictors	*Estimates*	*CI (95%)*	*p*
Intercept	33.29	26.18 – 40.39	**<0.001**
Psilocybin (vs Placebo)	7.42	-2.89 – 17.74	0.157
Post measurements	-6.82	-10.21 – -3.43	**<0.001**
Psilocybin * Post measurements	-6.99	-11.89 – -2.10	**0.005**
Random Effects			
σ2	84.20		
$\tau_{00\ id}$	231.40		
ICC	0.73		
N	40		
Data points	263		
REML	2010.9		
BIC	2044.38		
Marginal R^2 / Conditional R^2	0.076 / 0.754		

Note: σ2: error variance; ICC: Intraclass correlation coefficient; τ00: between-person variance.
Sample size: N=40 at timepoint -14d, -6d, +1d; n=37 at +14d, +30d; n=35 at +96d; n=34 at +190d. (d=days)

Confidence

**Per protocol analysis**			
Predictors	*Estimates*	*CI (95%)*	*p*
Intercept	48.50	40.99 – 56.01	**<0.001**
Psilocybin (vs Placebo)	-9.81	-20.58 – 0.97	0.074
Post measurements	4.41	-0.48 – 9.29	0.077
Psilocybin * Post measurements	6.84	-0.15 – 13.83	0.055
Random Effects			
σ2	164.36		
$\tau_{00\ id}$	194.32		
ICC	0.54		
N	37		
Data points	253		
REML	2070.4		
BIC	2103.6		
Marginal R^2 / Conditional R^2	0.055 / 0.567		

Note: σ2: error variance; ICC: Intraclass correlation coefficient; τ00: between-person variance
Sample size: N=37 at timepoint -14d, -6d, +1d, +30d; n=36 at +14d; n=35 at +96d; n=34 at +190d. (d=days)

**Intention-to-treat approach**			
Predictors	*Estimates*	*CI (95%)*	*p*
Intercept	48.71	41.71 – 55.72	**<0.001**
Psilocybin (vs Placebo)	-9.19	-19.36 – 0.98	0.076
Post measurements	4.06	-0.62 – 8.73	0.089
Psilocybin * Post measurements	7.18	0.43 – 13.94	**0.037**
Random Effects			
σ2	160.64		
$\tau_{00\ id}$	185.63		
ICC	0.54		
N	40		
Data points	263		
REML	2148.1		
BIC	2181.5		
Marginal R^2 / Conditional R^2	0.052 / 0.560		

Note: σ2: error variance; ICC: Intraclass correlation coefficient; τ00: between-person variance.
Sample size: N=40 at timepoint -14d, -6d, +1d; n=37 at +14d, +30d; n=35 at +96d; n=34 at +190d. (d=days)

Supplementary Table S3: Beck Depression Inventory: Random coefficient model details.

Predictors	*Estimates*	*CI (95%)*	*p*
Intercept	11.63	7.95 – 15.32	**<0.001**
Psilocybin (vs Placebo)	1.26	-4.03 – 6.54	0.640
Post measurements	-0.51	-2.48 – 1.47	0.612
Psilocybin * Post measurements	-4.71	-7.54 – -1.89	**0.001**
Random Effects			
σ2	26.84		
$\tau_{00\ id}$	53.14		
ICC	0.66		
N	37		
Data points	253		
REML	1635.6		
BIC	1668.8		
Marginal R^2 / Conditional R^2	0.046 / 0.680		

Note: σ2: error variance; ICC: Intraclass correlation coefficient;
τ00: between-person variance
Sample size: N=37 at timepoint -14d, -6d, +1d, +30d; n=36 at +14d;
n=35 at +96d; n=34 at +190d. (d=days)

Supplementary Table S4: Hopelessness Scale: Random coefficient model details.

Predictors	*Estimates*	*CI (95%)*	*p*
Intercept	4.74	2.87 – 6.60	**<0.001**
Psilocybin (vs Placebo)	0.87	-1.80 – 3.55	0.520
Post measurements	0.43	-0.52 – 1.39	0.371
Psilocybin * Post measurements	-1.54	-2.91 – -0.17	**0.028**
Random Effects			
σ2	3.68		
$\tau_{00\ id}$	13.29		
ICC	0.78		
N	37		
Data points	216		
REML	1000.9		
BIC	1033.2		
Marginal R^2 / Conditional R^2	0.008 / 0.785		

Note: σ2: error variance; ICC: Intraclass correlation coefficient;
τ00: between-person variance
Sample size: N=37 at timepoint -14d, -6d, +1d, +30d; n=36 at +14d;
n=35 at +98d; n=34 at +190d. (d=days)

Supplementary Table S5: Emotion Regulation Questionnaire: Random coefficient model details.

Cognitive Reappraisal: Nullmodel

Predictors	*Estimates*	*CI (95%)*	*p*
Intercept	28.60	26.60 – 30.59	**<0.001**
Random Effects			
σ2	11.49		
$\tau_{00\,id}$	36.05		
ICC	0.76		
N	37		
Data points	216		
REML	1246.8		
BIC	1263		
Marginal R^2 / Conditional R^2	0.000 / 0.758		

Note: σ2: error variance; ICC: Intraclass correlation coefficient;
τ00: between-person variance
Sample size: N=37 at timepoint -6d, +1d, +30d; n=36 at +14d;
n=35 at +96d; n=34 at +190d. (d=days)

Expressive Suppression

Predictors	*Estimates*	*CI (95%)*	*p*
Intercept	13.53	11.16 – 15.89	**<0.001**
Psilocybin (vs Placebo)	2.64	-0.75 – 6.03	0.126
Post measurements	1.43	0.15 – 2.70	**0.028**
Psilocybin * Post measurements	-2.29	-4.12 – -0.47	**0.014**
Random Effects			
σ2	6.52		
$\tau_{00\,id}$	20.75		
ICC	0.76		
N	37		
Data points	216		
REML	1117.9		
BIC	1150.1		
Marginal R^2 / Conditional R^2	0.012 / 0.764		

Note: σ2: error variance; ICC: Intraclass correlation coefficient;
τ00: between-person variance
Sample size: N=37 at timepoint -6d, +1d, +30d; n=36 at +14d;
n=35 at +96d; n=34 at +190d. (d=days)

Supplementary Table S6: Positive and Negative Affect Schedule: Random coefficient model details.

Positive Affect: Nullmodel

Predictors	*Estimates*	*CI (95%)*	*p*
Intercept	31.42	29.26 – 33.59	**<0.001**
Random Effects			
σ2	20.81		
$\tau_{00\,id}$	42.10		
ICC	0.67		
N	37		
Data points	290		
REML	1804.7		
BIC	1821.7		
Marginal R^2 / Conditional R^2	0.000 / 0.669		

Note: σ2: error variance; ICC: Intraclass correlation coefficient;
τ00: between-person variance
Sample size: N=37 at timepoint -14d, -6d, day 0, +1d, +30d; n=36 at +14d;
n=35 at +96d; n=34 at +190d. (d=days)

Negative Affect

Predictors	*Estimates*	*CI (95%)*	*p*
Intercept	19.84	16.98 – 22.71	**<0.001**
Psilocybin (vs Placebo)	0.27	-3.84 – 4.38	0.898
Post measurements	-1.19	-2.45 – 0.07	0.063
Psilocybin * Post measurements	-2.24	-4.04 – -0.44	**0.015**
Random Effects			
σ2	14.27		
$\tau_{00\,id}$	35.55		
ICC	0.71		
N	37		
Data points	290		
REML	1694.6		
BIC	1728.6		
Marginal R^2 / Conditional R^2	0.036 / 0.724		

Note: σ2: error variance; ICC: Intraclass correlation coefficient;
τ00: between-person variance
Sample size: N=37 at timepoint -14d, -6d, day 0, +1d, +30d; n=36 at +14d;
n=35 at +96d; n=34 at +190d. (d=days)

Supplementary Table S7: Quality of life: Random coefficient model details.

Total quality of life

Predictors	*Estimates*	*CI (95%)*	*p*
Intercept	115.37	108.41 – 122.33	**<0.001**
Psilocybin (vs Placebo)	-0.95	-10.93 – 9.03	0.851
Post measurements	0.72	-2.21 – 3.65	0.628
Psilocybin * Post measurements	4.93	0.74 – 9.13	**0.021**
Random Effects			
σ2	59.14		
$\tau_{00\ id}$	207.62		
ICC	0.78		
N	37		
Data points	253		
REML	1851.3		
BIC	1884.5		
Marginal R^2 / Conditional R^2	0.018 / 0.782		

Note: σ2: error variance; ICC: Intraclass correlation coefficient;
τ00: between-person variance
Sample size: N=37 at timepoint -14d, -6d, +1d, +30d; n=36 at +14d;
n=35 at +96d; n=34 at +190d. (d=days)

Physical Health

Predictors	*Estimates*	*CI (95%)*	*p*
Intercept	15.22	14.08 – 16.35	**<0.001**
Psilocybin (vs Placebo)	-0.28	-1.91 – 1.35	0.734
Post measurements	-0.07	-0.68 – 0.55	0.825
Psilocybin * Post measurements	0.97	0.09 – 1.85	**0.032**
Random Effects			
σ2	2.61		
$\tau_{00\ id}$	5.01		
ICC	0.66		
N	37		
Data points	253		
REML	1054		
BIC	1087.2		
Marginal R^2 / Conditional R^2	0.016 / 0.663		

Note: σ2: error variance; ICC: Intraclass correlation coefficient;
τ00: between-person variance
Sample size: N=37 at timepoint -14d, -6d, +1d, +30d; n=36 at +14d;
n=35 at +96d; n=34 at +190d. (d=days)

Psychological Health: Nullmodel

Predictors	*Estimates*	*CI (95%)*	*p*
Intercept	14.14	13.40 – 14.87	**<0.001**
Random Effects			
σ2	1.70		
$\tau_{00\ id}$	4.91		
ICC	0.74		
N	37		
Data points	253		
REML	949.6		
BIC	979.6		
Marginal R^2 / Conditional R^2	0.000 / 0.743		

Note: σ2: error variance; ICC: Intraclass correlation coefficient;
τ00: between-person variance
Sample size: N=37 at timepoint -14d, -6d, +1d, +30d; n=36 at +14d;
n=35 at +96d; n=34 at +190d. (d=days)

Social Relationships: Nullmodel

Predictors	*Estimates*	*CI (95%)*	*p*
Intercept	14.46	13.44 – 15.47	**<0.001**
Random Effects			
σ2	2.83		
$\tau_{00\ id}$	9.36		
ICC	0.77		
N	37		
Data points	253		
REML	1096.2		
BIC	1112.8		
Marginal R^2 / Conditional R^2	0.000 / 0.768		

Note: σ2: error variance; ICC: Intraclass correlation coefficient;
τ00: between-person variance
Sample size: N=37 at timepoint -14d, -6d, +1d, +30d; n=36 at +14d;
n=35 at +96d; n=34 at +190d. (d=days)

Environment

Predictors	*Estimates*	*CI (95%)*	*p*
Intercept	15.88	14.86 – 16.90	**<0.001**
Psilocybin (vs Placebo)	-0.03	-1.50 – 1.43	0.963
Post measurements	0.03	-0.37 – 0.42	0.894
Psilocybin * Post measurements	0.71	0.15 – 1.27	**0.013**
Random Effects			
σ2	1.06		
$\tau_{00\ id}$	4.56		
ICC	0.81		
N	37		
Data points	253		
REML	856.4		
BIC	889.7		
Marginal R^2 / Conditional R^2	0.019 / 0.815		

Note: σ2: error variance; ICC: Intraclass correlation coefficient;
τ00: between-person variance
Sample size: N=37 at timepoint -14d, -6d, +1d, +30d; n=36 at +14d;
n=35 at +96d; n=34 at +190d. (d=days)

Supplementary Table S8: Snaith Hamilton Pleasure Scale: Random coefficient model details.

**Snaith Hamilton Pleasure Scale:** Nullmodel			
Predictors	*Estimates*	*CI (95%)*	*p*
Intercept	6.91	5.55 – 8.27	**<0.001**
Random Effects			
σ2	7.28		
$\tau_{00\ id}$	16.37		
ICC	0.69		
N	37		
Data points	221		
REML	1162.5		
BIC	1178.7		
Marginal R^2 / Conditional R^2	0.000 / 0.692		

Note: σ2: error variance; ICC: Intraclass correlation coefficient;
τ00: between-person variance
Sample size: N=37 at timepoint -6d, +1d, +30d; n=36 at +14d;
n=35 at +96d; n=34 at +190d. (d=days)

Supplementary Table S9: Temporary Experience of Pleasure Scale: Random coefficient model details.

Total

Predictors	*Estimates*	*CI (95%)*	*p*
Intercept	82.33	79.23 – 85.44	**<0.001**
Random Effects			
σ2	35.79		
$\tau_{00\,id}$	85.50		
ICC	0.70		
N	37		
Data points	215		
REML	1475.3		
BIC	1491.4		
Marginal R^2 / Conditional R^2	0.000 / 0.705		

Note: σ2: error variance; ICC: Intraclass correlation coefficient;
τ00: between-person variance
Sample size: N=37 at timepoint -6d, +1d, +30d; n=36 at +14d;
n=35 at +96d; n=34 at +190d. (d=days)

Consumatory

Predictors	*Estimates*	*CI (95%)*	*p*
Intercept	39.56	38.04 – 41.08	**<0.001**
Random Effects			
σ2	9.71		
$\tau_{00\,id}$	20.25		
ICC	0.68		
N	37		
Data points	215		
REML	1191.6		
BIC	1207.7		
Marginal R^2 / Conditional R^2	0.000 / 0.676		

Note: σ2: error variance; ICC: Intraclass correlation coefficient;
τ00: between-person variance
Sample size: N=37 at timepoint -6d, +1d, +30d; n=36 at +14d;
n=35 at +96d; n=34 at +190d. (d=days)

Anticipatory

Predictors	*Estimates*	*CI (95%)*	*p*
Intercept	42.77	40.86 – 44.68	**<0.001**
Random Effects			
σ2	11.83		
$\tau_{00\,id}$	32.71		
ICC	0.73		
N	37		
Data points	215		
REML	1243.4		
BIC	1259.5		
Marginal R^2 / Conditional R^2	0.000 / 0.734		

Note: σ2: error variance; ICC: Intraclass correlation coefficient;
τ00: between-person variance
Sample size: N=37 at timepoint -6d, +1d, +30d; n=36 at +14d;
n=35 at +96d; n=34 at +190d. (d=days)

Supplementary Table S10: Symptom Checklist. Mean(SD)

	PSILOCYBIN		PLACEBO		Welch's t-Test: 4-week follow-up				
**Subscale**	**Screening**	**4-weeks follow-up** (n=18)	**Screening**	**4-weeks follow-up** (n=19)	**mean difference:** 4-weeks - Screening (Psi-Pla)	**t-value**	**df**	**CI (95%)**	***p*-value**
Somatization	0.54 (0.72)	0.38 (0.40)	0.40 (0.40)	0.40 (0.40)	-0.16	-1.03	24.98	-0.47 - 0.16	0.31
Obsessive-compulsive	0.77 (0.80)	0.51 (0.63)	0.81 (0.65)	0.70 (0.58)	-0.16	-1.11	34.82	-0.44 - 0.13	0.27
Interpersonal sensitivity	0.53 (0.48)	0.40 (0.46)	0.54 (0.57)	0.59 (0.68)	-0.18	-1.40	34.98	-0.45 - 0.08	0.17
Anger-hostility	0.31 (0.41)	0.27 (0.31)	0.37 (0.43)	0.47 (0.55)	-0.13	-1.13	34.98	-0.37 - 0.11	0.27
Anxiety	0.59 (0.64)	0.27 (0.31)	0.57 (0.57)	0.45 (0.47)	-0.21	-1.31	28.32	-0.53 - 0.12	0.20
Depression	0.75 (0.86)	0.52 (0.53)	0.75 (0.63)	0.72 (0.67)	-0.20	-1.19	29.59	-0.23 - 0.03	0.25
Psychoticism	0.30 (0.36)	0.22 (0.26)	0.26 (0.31)	0.24 (0.33)	-0.06	-0.69	34.95	-0.08 - 0.02	0.50
Paranoid Ideation	0.24 (0.24)	0.19 (0.24)	0.40 (0.50)	0.49 (0.44)	-0.14	-1.36	27.74	-0.06 - 0.09	0.18
Phobic Anxiety	0.19 (0.20)	0.13 (0.23)	0.20 (0.34)	0.21 (0.30)	-0.07	-0.77	33.84	-0.26 - 0.11	0.45
Global Severity Index	0.53 (0.52)	0.36 (0.34)	0.52 (0.42)	0.51 (0.45)	-0.16	-1.45	30.41	-0.37 - 0.06	0.16

Supplementary Table S11: Safety Reporting.

Adverse Events

	Psilocybin (n=18)	Placebo (n=19)
**Prior to substance visit**		
Flu	2 (11%)	0
Gout	0	1 (5%)
Dizziness	0	1 (5%)
**Acute adverse events**		
Nausea	2 (11%)	0
Chest pressure	1 (6%)	0
Hypoglycemia	1 (6%)	0
Migraine	1 (6%)	0
**1 day post substance visit**		
Headache	5 (28%)	0
Physical pain	0	1 (5%)
**2 weeks post substance visit**		
Flu	1 (6%)	3 (16%)
Difficulties with memory	1 (6%)	0
Physical pain	0	1 (5%)
Stress	0	1 (5%)
Nausea	0	1 (5%)
**4 weeks post substance visit**		
Flu	2 (11%)	0

Serious Adverse Events

	Psilocybin (n=18)	Placebo (n=19)
**2 weeks post substance visit**		
Hospitalized withdrawal treatment	0	1 (5%)
**4 weeks post substance visit**		
Hospitalized withdrawal treatment	0	1 (5%)
**3 months post substance visit**		
Hospitalized withdrawal treatment	0	1 (5%)
**6 months post substance visit**		
Hospitalized withdrawal treatment	1 (6%)	1 (5%)

References

1 Starosta AN, Leeman RF, Volpicelli JR. The BRENDA Model: Integrating Psychosocial Treatment and Pharmacotherapy for the Treatment of Alcohol Use Disorders. *J Psychiatr Pract* 2006; **12:** 80–89.
2 Monterosso JR, Flannery BA, Pettinati HM, et al. Predicting treatment response to naltrexone: the influence of craving and family history. *Am J Addict* 2001; **10:** 258–68. https://doi.org/10.1080/105504901750532148.
3 Pettinati HM, Volpicelli JR, Pierce JD, O'Brien CP. Improving naltrexone response: an intervention for medical practitioners to enhance medication compliance in alcohol dependent patients. *J Addict Dis* 2000; **19:** 71–83. https://doi.org/10.1300/J069v19n01_06.
4 Flannery BA, Volpicelli JR, Pettinati HM. Psychometric properties of the Penn Alcohol Craving Scale. *Alcohol Clin Exp Res* 1999; **23:** 1289–95.
5 DiClemente CC, Carbonari JP, Montgomery RP, Hughes SO. The Alcohol Abstinence Self-Efficacy scale. *J Stud Alcohol* 1994; **55:** 141–48. https://doi.org/10.15288/jsa.1994.55.141.
6 Beck AT, Ward CH, Mendelson M, Mock J, Erbaugh J. An inventory for measuring depression. *Arch Gen Psychiatry* 1961; **4:** 561–71. https://doi.org/10.1001/archpsyc.1961.01710120031004.
7 Kühner C, Bürger C, Keller F, Hautzinger M. Reliabilität und Validität des revidierten Beck-Depressionsinventars (BDI-II). Befunde aus deutschsprachigen Stichproben. *Nervenarzt* 2007; **78:** 651–56. https://doi.org/10.1007/s00115-006-2098-7.
8 Beck AT, Steer RA, Carbin MG. Psychometric properties of the Beck Depression Inventory: Twenty-five years of evaluation. *Clin Psychol Rev* 1988; **8:** 77–100. https://doi.org/10.1016/0272-7358(88)90050-5.
9 Krampen G, Beck AT. Skalen zur Erfassung von Hoffnungslosigkeit, H-Skalen: Handanweisung, 1994.
10 Beck AT, Weissman A, Lester D, Trexler L The measurement of pessimism: the hopelessness scale. *J Consult Clin Psychol* 1974; **42:** 861–65. https://doi.org/10.1037/h0037562.
11 Gross JJ, John OP. Individual differences in two emotion regulation processes: implications for affect, relationships, and well-being. *J Pers Soc Psychol* 2003; **85:** 348–62. https://doi.org/10.1037/0022-3514.85.2.348.
12 Watson D, Clark LA, Tellegen A. Development and validation of brief measures of positive and negative affect: the PANAS scales. *J Pers Soc Psychol* 1988; **54:** 1063–70. https://doi.org/10.1037//0022-3514.54.6.1063.
13 Krohne HW, Egloff B, Kohlmann C-W, Tausch A. PsycTESTS Dataset, 1996.
14 The WHOQOL Group. The World Health Organization Quality of Life Assessment (WHOQOL-BREF): Introduction, administration, scoring and generic version of the assessment 1996.
15 Snaith RP, Hamilton M, Morley S, Humayan A, Hargreaves D, Trigwell P. A scale for the assessment of hedonic tone the Snaith-Hamilton Pleasure Scale. *Br J Psychiatry* 1995; **167:** 99–103. https://doi.org/10.1192/bjp.167.1.99.
16 Simon JJ, Zimmermann J, Cordeiro SA, et al. Psychometric evaluation of the Temporal Experience of Pleasure Scale (TEPS) in a German sample. *Psychiatry Res* 2018; **260:** 138–43. https://doi.org/10.1016/j.psychres.2017.11.060.
17 Franke G. Die Symptom-Checkliste von Derogatis: Manual. SCL-90-R; Deutsche Version, 1995.
18 Dittrich A. The standardized psychometric assessment of altered states of consciousness (ASCs) in humans. *Pharmacopsychiatry* 1998; **31 Suppl 2:** 80–84. https://doi.org/10.1055/s-2007-979351.
19 Bogenschutz MP, Ross S, Bhatt S, et al. Percentage of Heavy Drinking Days Following Psilocybin-Assisted Psychotherapy vs Placebo in the Treatment of Adult Patients With Alcohol Use Disorder: A Randomized Clinical Trial. *JAMA Psychiatry* 2022. https://doi.org/10.1001/jamapsychiatry.2022.2096.
20 Holze F, Gasser P, Müller F, Dolder PC, Liechti ME. Lysergic acid diethylamide-assisted therapy in patients with anxiety with and without a life-threatening illness A randomized, double-blind, placebo-controlled Phase II study. *Biological Psychiatry* 2022. https://doi.org/10.1016/j.biopsych.2022.08.025.
21 Garcia-Romeu A, Griffiths RR, Johnson MW. Psilocybin-occasioned mystical experiences in the treatment of tobacco addiction. *Curr Drug Abuse Rev* 2014; **7:** 157–64. https://doi.org/10.2174/1874473708666150107121331.
22 Johnson MW, Garcia-Romeu A, Griffiths RR. Long-term follow-up of psilocybin-facilitated smoking cessation. *Am J Drug Alcohol Abuse* 2017; **43:** 55–60. https://doi.org/10.3109/00952990.2016.1170135.
23 Bogenschutz MP, Forcehimes AA, Pommy JA, Wilcox CE, Barbosa PCR, Strassman RJ. Psilocybin-assisted treatment for alcohol dependence: a proof-of-concept study. *J Psychopharmacol (Oxford)* 2015; **29:** 289–99. https://doi.org/10.1177/0269881114565144.
