# Supplementary material for: Psilocybin-assisted therapy for relapse prevention in alcohol use disorder: a phase 2 randomized clinical trial
Source: eClinicalMedicine. 2025 Mar 14;82:103149. doi: 10.1016/j.eclinm.2025.103149 (PMC11937691; doi:10.1016/j.eclinm.2025.103149)
Supplement: Informed Consent [file mmc3.pdf]

Studieninformation

Klinische und mechanistische Effekte von Psilocybin bei Patienten mit einer Alkoholabhängigkeit

Phase II, randomisierte, doppelverblindete, placebo-kontrollierte, Parallelgruppen, monozentrische Studie über die Wirksamkeit und Wirkungsweise von Psilocybin in Patienten mit einer Alkoholabhängigkeit

Diese Studie ist organisiert durch:
Prof. Dr. med. Franz X. Vollenweider
Department of Psychiatry, Psychotherapy and Psychosomatics
Neuropsychopharmacology and Brain Imaging
Psychiatric Hospital, University of Zurich
Lenggstrasse 31
CH-8032 Zürich,
Switzerland
E-mail: vollen@bli.uzh.ch
Phone: +41 58 384 24 04

Sehr geehrte Dame, sehr geehrter Herr,

Wir möchten Sie anfragen, ob Sie an einer klinischen Studie teilnehmen wollen. Im Folgenden wird Ihnen dieses Studienvorhaben dargestellt: zunächst in einer kurzen Zusammenfassung, damit Sie wissen, um was es geht, anschliessend in einer detaillierten Beschreibung.

Zusammenfassung

1	**Ziel der Studie**<br>Wir möchten Sie hiermit bitten, an unserer Heilmittelstudie mit Psilocybin teilzunehmen. Die Studie untersucht Patienten mit einer Alkoholabhängigkeit nachdem eine Entzugsbehandlung abgeschlossen wurde. Wir führen diese Studie durch, um die Wirkung von Psilocybin auf Ihre Symptome und Gehirnaktivität zu erforschen.
2	**Auswahl**<br>Sie leiden unter einer Alkoholabhängigkeit. Deshalb lassen wir Ihnen diese Informationsschrift zukommen.
3	**Allgemeine Informationen zur Studie**<br>In dieser Studie wollen wir untersuchen, wie gut Psilocybin in der Behandlung von Alkoholabhängigkeit wirkt und mittels welchen Wirkungsmechanismen.<br>Insgesamt werden wir 60 Patienten mit der Diagnose der Alkoholabhängigkeit einschliessen nachdem eine Entzugsbehandlung abgeschlossen wurde. Die Teilnehmer werden zwei Gruppen zugeordnet. Die Teilnehmenden der ersten Gruppe nehmen einmalig Psilocybin ein – die Substanz, die wir untersuchen wollen. Die Teilnehmenden in der zweiten Gruppe erhalten ein Scheinmedikament (Placebo). Ein Placebo sieht aus wie die Substanz, enthält aber keinen Wirkstoff. In dieser Studie besteht das Placebo aus Zucker. Die Zuteilung in die Studiengruppen erfolgt „randomisiert“, d.h. es wird ausgelost, wer in welche Gruppe kommt. Es ist bei einem solchen Test also Zufall, ob man das echte Medikament oder Placebo erhält. Die Wahrscheinlichkeit, dass Sie mit dem Prüfpräparat behandelt werden, beträgt 50%. Die Studientermine beinhalten unter anderem wiederholte funktionelle Magnetresonanztomographie (fMRT) Untersuchungen, neuropsychologische Untersuchungen (MET), sowie Blutentnahmen zur Bestimmung mehrerer genetischer Marker zur Vorhersage der Reaktion auf die Behandlung. Zudem werden Sie angehalten, Fragebogen zu Ihrem Alkohol- und Substanzkonsum und Ihrem physischen und psychischen Zustand auszufüllen. Die psychologischen Gespräche mit dem Versuchsleiter sowie Behandlungssitzungen werden auf Tonband aufgezeichnet. Die Studie setzt sich zusammen aus sechs Sitzungen die an der Psychiatrischen Universitätsklinik stattfinden (über sechs Wochen verteilt) und zwei Folge-Untersuchungen, welche Sie von Zuhause aus ausfüllen können (drei und sechs Monate später).
4	**Ablauf**<br>Die Studie findet an der Psychiatrischen Universitätsklinik Zürich statt. Zuerst findet ein Screening

	Besuch von ca. 5 Stunden Dauer statt. Nach der Screening-untersuchung werden wir Ihnen mitteilen, ob Sie in die Studie eingeschlossen werden. Darauffolgend findet eine Baselinesitzung à ca. 5h statt. Etwa drei Tage danach werden Sie an der ca. 8h Behandlungssitzung das Placebo oder das Psilocybin einnehmen. Am Tag danach findet eine Nachbesprechung von 3h statt. Nach einer kurzen Pause werden Sie nochmals für zwei weitere Sitzungen (Verlaufsgespräch: 3h und Abschlussmessung: 4h) an die PUK reisen. Zuletzt werden Sie drei und sechs Monate nach der eigentlichen Studie an zwei Folge-Untersuchungen teilnehmen, für welches Sie Zuhause 1.5h einrechnen sollten.
5	**Nutzen**<br>Es steht für Sie kein individueller Nutzen in Aussicht.
6	**Rechte**<br>Sie entscheiden freiwillig, ob Sie an der Studie teilnehmen wollen oder nicht. Ihre Entscheidung hat keinen Einfluss auf Ihre medizinische Behandlung und Sie müssen diese Entscheidung nicht begründen.
7	**Pflichten**<br>Wenn Sie teilnehmen, müssen Sie zu Ihrer Sicherheit bestimmte Regeln befolgen. Als Studienteilnehmende/r sind Sie dazu verpflichtet:<br>▪ Ihr psychisches Befinden wahrheitsgetreu Ihrem Studienarzt mitzuteilen; insbesondere müssen Sie sich bei Verschlechterung Ihres psychischen Befindens mit Ihrem Studienarzt oder Versuchsleitung in Verbindung setzen;<br>▪ Den medizinischen Anweisungen Ihres Studienarztes zu folgen und sich an den Studienplan zu halten;<br>▪ Innerhalb von sechs Wochen vor dem Studienstart müssen Sie ein Entzugsprogramm abgeschlossen haben;<br>▪ Ihren Studienarzt über den Verlauf der Erkrankung zu informieren und neue Symptome, neue Beschwerden und Änderungen im Befinden zu melden;<br>▪ Ihren Studienarzt über die gleichzeitige Behandlung und Therapien bei einem anderen Arzt und über die Einnahme von Medikamenten zu informieren;<br>▪ Am Behandlungstag kein Fahrzeug zu lenken oder Maschinen zu bedienen.
8	**Risiken**<br>Psilocybin ist körperlich sehr gut verträglich und hat ein sehr geringes Abhängigkeitsrisiko. Trotzdem können einzelne körperliche oder psychische Nebenwirkungen auftreten, die in den allermeisten Fällen kurzfristiger Natur sind. Diese sind: Verstärkung oder Auslösung emotionaler Erlebnisse oder Reaktionen, vorübergehende psychotische Symptome (z.B. Wahngedanken, Paranoia, Halluzinationen), wiederkehrende Halluzinationen nach Abklingen der akuten Substanzwirkung („flashbacks“), Kopfschmerzen und Erschöpfung („Katergefühl“), Blutdruckerhöhung und Pulsbeschleunigung.
9	**Andere Behandlungsmöglichkeiten**<br>Ihr Arzt wird Sie beraten, welche anderen Möglichkeiten zu Ihrer Behandlung bestehen.
10	**Ergebnisse**<br>Bei Studienergebnissen und Zufallsbefunden während der Studie werden Sie informiert, wenn diese Ergebnisse für Sie gesundheitlich wichtig sind. Falls Sie dies nicht möchten, informieren Sie bitte Ihren Prüfarzt.
11	**Vertraulichkeit von Daten und Proben**<br>Wir halten alle gesetzlichen Regeln des Datenschutzes ein und alle Beteiligten unterliegen der Schweigepflicht. Ihre persönlichen und medizinischen Daten und Ihr biologisches Material/ Ihre Proben (Blut, Urin, etc.) werden verschlüsselt verwendet und geschützt. Die Daten und Proben werden für andere Forschungsprojekte weiter verwendet, wenn Sie Ihr separates Einverständnis dafür geben.
12	**Rücktritt**<br>Sie können jederzeit von der Studie zurück treten und nicht mehr teilnehmen. Die bis dahin erhobenen Daten und Proben werden noch ausgewertet.

13	**Entschädigung**<br>Sie erhalten keine Entschädigung.
14	**Haftung**<br>Die Zürich Versicherungs-Gesellschaft kommt für Schäden im Rahmen der Studie auf.
15	**Finanzierung**<br>Die Studie wird vom Schweizerischen Nationalfonds zur Förderung der wissenschaftlichen Forschung bezahlt.
16	**Kontaktpersonen:**<br>Sie erhalten jederzeit auf alle Ihre Fragen Auskunft.<br><br>Studienkoordination:<br>Nathalie Rieser<br>Psychiatrische Universitätsklinik Zürich<br>Postfach 1931, 8032 Zürich<br>Tel.: +41 58 384 33 24<br>Email: nathalie.rieser@bli.uzh.ch<br><br>StudienärztInnen:<br>Dr. med. Raoul Bitar<br>Psychiatrische Universitätsklinik Zürich<br>Postfach 1931, 8032 Zürich<br>Tel.: +41 58 384 33 77<br>Email: raoul.bitar@pukzh.ch<br><br>Dr. med. Christina Rossgoderer Pinto da Silva<br>Psychiatric Hospital, University of Zurich<br>Lenggstrasse 31<br>CH-8032 Zürich,<br>Phone: +41 58 384 2329<br>E-mail: christina.rossgoderer@pukzh.ch<br><br>Pract. med. Simon Halm<br>Department of Psychiatry, Psychotherapy and Psychosomatics<br>Center for Addiction Disorders<br>Selnaustrasse 9<br>CH-8001 Zürich,<br>Switzerland<br>Phone: +41 44 205 58 60<br>E-mail: simon.halm@pukzh.ch<br><br>Leiter der Studie:<br>PD Dr. med. Marcus Herdener<br>Department of Psychiatry, Psychotherapy and Psychosomatics<br>Center for Addiction Disorders<br>Selnaustrasse 9<br>CH-8001 Zürich,<br>Switzerland<br>Phone: +41 44 205 58 11<br>E-mail: marcus.herdener@bli.uzh.ch<br><br>Notfallnummer, 24h Erreichbarkeit: 0800 33 66 55

Detailliertere Information

1. Ziel der Studie

Wir wollen untersuchen, ob und wie gut der Wirkstoff Psilocybin in der Behandlung der Alkoholabhängigkeit wirkt und welche Vorgänge im Gehirn und im Blut damit verbunden sind. Im Rahmen von internationalen klinischen Studien wird die Substanz derzeit ausserdem zur Behandlung von Angsterkrankungen, Zwangsstörungen und anderen Abhängigkeitserkrankungen erprobt. Psilocybin ist ein halluzinogener (Halluzinationen bzw. Pseudo-Halluzinationen erzeugender) Wirkstoff, welcher in den mexikanischen Zauberpilzen der Gattung Psilocybe vorkommt und von den indigenen Kulturen seit Jahrtausenden zur Erzeugung von aussergewöhnlichen Bewusstseinszuständen im Rahmen religiöser Zeremonien und Heilpraktiken angewendet wird. Der Wirkstoff wurde 1957 erstmals vom Schweizer Chemiker Albert Hoffmann isoliert und 1958 chemisch hergestellt. Psilocybin wurde in den 1960er Jahren durch die Firma Sandoz, Basel, in Form des Medikamentes Indocybin® zur Verwendung in psychiatrischen und klinischen Studien zugelassen. Seit den 1990er Jahren wird die Substanz Psilocybin in unserem Labor zur Erforschung von veränderten Bewusstseinszuständen und deren therapeutische Anwendung angewendet. Studien haben gezeigt, dass Psilocybin hilft, den Alkoholkonsum zu reduzieren.

Psilocybin entfaltet seine Wirkung über spezifische Andockstellen im Hirn (Neurorezeptoren), insbesondere für den Botenstoff (Neurotransmitter) Serotonin. Etwa 20 bis 40 Minuten nach Einnahme von Psilocybin (bei uns 25mg in Form von einer Kapsel) kommt es in den meisten Fällen zu einer deutlichen Veränderung des Bewusstseinszustandes, welcher 60-90 Minuten nach Einnahme am stärksten ist und sich innerhalb von 4-6 Stunden vollständig zurückbildet. Psilocybin führt dosisabhängig zu (meist als sehr angenehm erlebten) Veränderungen der Stimmung, zu Veränderung der Wahrnehmung (meist des Sehens, aber auch des Hörens oder des Körperempfindens), zu Veränderung des Erlebens von Zeit, Raum und Selbst. Unter der Wirkung von Psilocybin kommt es bei gutem Erinnerungsvermögen zu tagtraumartigen Imaginationen vor geschlossenen Augen und einer Aktivierung der Emotionen sowie der Sinnesfunktionen. Das Gefühlserleben ist gesteigert, Vorstellungen sind intensiviert, sodass Trugwahrnehmungen (Wahrnehmungen, die nicht mit der Realität übereinstimmen) entstehen. Die Gedanken sind bildhafter, es können emotional bedeutsame Einsichten gewonnen werden und unbewusste psychische Inhalte treten hervor. Diese psychischen Inhalte sind häufig von hoher Relevanz zum Verständnis der Krankheitsentstehung und -aufrechterhaltung und ähneln in ihrer Form der Traumsymbolik. Häufig gelingt es dem Patienten aus einer Beobachterperspektive, nach dem Prinzip eines Weitwinkelobjektives, weit auseinanderliegende innerseelische Fakten wie Erinnerungen, menschliche Beziehungen, Gefühlserlebnisse oder fehlerhafte charakterliche Einstellungen miteinander in Sinnzusammenhang zu bringen. Der Betreffende kann eine Fülle introspektiver Einsichten gewinnen, was einen therapeutischen Effekt bewirken kann.
Um Krankheiten zu verstehen, ist es wichtig, mehr über die zugrundeliegenden biologischen Abläufe zu erfahren. So wissen wir heute, dass zum Beispiel die Erbsubstanz (Gene) bei der Entstehung und Behandlung von Krankheiten eine wichtige Rolle spielt. Diese wollen wir in dieser Studie ebenfalls untersuchen.

2. Auswahl

Es können alle Personen teilnehmen, die an einer Alkoholabhängigkeit leiden. Ausserdem müssen Sie zwischen 18 und 60 Jahre alt sein und Rechtshänder/Innen sein. Sie müssen innerhalb von sechs Wochen vor Studienbeginn einen Alkoholentzug durchlaufen haben und dürfen ab dem Studienstart bis zur Substanz- oder Placeboeinnahme keinen Alkohol konsumieren; Sie dürfen mindestens fünf Tage vor der Substanz- oder Placeboeinnahme bis zum Ende der Studie keine anderen psychisch wirksamen Medikamente einnehmen (ausser in Notfällen); Sie müssen in guter, stabiler körperlicher Verfassung sein und gut (Schweizer)Deutsch sprechen und verstehen. Frauen im gebärfähigen Alter müssen während der Studie auf eine ausreichende Schwangerschaftsverhütung achten (z.B. „Pille“, Spirale, zusätzlich Kondom beim Partner).

Nicht teilnehmen dürfen Personen, die im Rahmen einer früheren Einnahme von Psilocybin allergische oder Überempfindlichkeitsreaktionen oder andere Nebenwirkungen gezeigt haben. Frauen, die schwanger sind oder Personen die in den letzten zehn Jahren mehr als 10 Mal halluzinogene Substanzen konsumiert haben. Personen, die an einer schwerwiegenden psychischen oder physischen Erkrankung erkrankt sind, werden auch von der Studie ausgeschlossen. Auch für die MRT-Messungen muss ihre Sicherheit gewährleistet werden, weshalb Personen bei denen Kontraindikationen für die Magnetresonanztomographie-Untersuchungen bestehen, nicht teilnehmen können (z.B. Herzschrittmacher, Metallsplitter, grossflächige Tätowierungen, etc.).
Bitte informieren Sie die Versuchsleitung, falls einer dieser Punkte auf Sie zutrifft.

3. Allgemeine Informationen

- Die Psilocybin-Studie findet an der Psychiatrischen Universitätsklinik Zürich statt. Psilocybin ist derzeit nicht als Medikament zugelassen, wird jedoch sowohl national als auch international bei gesunden Probanden und bei Patienten im Rahmen von klinischen Studien angewendet. In dieser Studie wollen wir untersuchen, wie gut Psilocybin bei der Behandlung von Alkoholabhängigkeit wirkt. Dazu teilen wir die insgesamt 60 Teilnehmenden zwei verschiedenen Gruppen zu. Den Teilnehmenden in der ersten Gruppe werden wir Psilocybin abgeben – die Substanz, die wir untersuchen wollen. Die Teilnehmenden in der zweiten Gruppe erhalten ein Scheinmedikament (Placebo – enthält kein Wirkstoff). Ob Sie Psilocybin oder das Placebo erhalten ist zufällig, die Wahrscheinlichkeit beträgt also 50%. Ausserdem wissen weder wir, noch Sie, was einnehmen werden. In einem Notfall kann diese Verblindung jedoch aufgehoben werden.
- Psilocybin wird einmalig in Kapselform (insgesamt 25mg) oral (durch Schlucken) verabreicht. Diese Dosierung wurde auch schon in vorherigen Studien verwendet und zeigte gute Verträglichkeit.
- Der erste Teil der Studie an der Psychiatrischen Universitätsklinik (PUK) streckt sich über sechs Wochen hinweg. Anschliessend finden zwei Folge-Untersuchungen statt (drei und sechs Monate nach der Behandlungssitzung), welche von Zuhause aus in Onlinefragebögen eingetragen werden können.
- Wir machen diese Studie so, wie es die Gesetze in der Schweiz vorschreiben. Ausserdem beachten wir alle international anerkannten Richtlinien. Die zuständige Kantonale Ethikkommission und Swissmedic haben die Studie geprüft und bewilligt.
- Eine Beschreibung dieser Studie finden Sie auch auf der Internetseite des Bundesamtes für Gesundheit: www.kofam.ch.

4. Ablauf

- Die gesamte Studie findet an der Psychiatrischen Universitätsklinik Zürich statt. Zuerst findet eine Screening-Untersuchung von ca. 5 Stunden Dauer statt. Danach werden wir Ihnen mitteilen, ob Sie an der Studie teilnehmen können oder nicht. Anschliessend findet eine Baseline-Messung von ca. 5 Stunden Dauer, eine Behandlungssitzung von ca. 8 Stunden Dauer, zwei Verlaufsmessungen von ca. 3 Stunden Dauer, sowie eine Abschlussmessung von ca. 4 Stunden Dauer über einen Zeitraum von sechs Wochen statt. Anschliessend werden von Zuhause aus zwei Folge-Untersuchungen-Fragebögen von je ca. 1.5 Stunden ausgefüllt (3 und 6 Monate nach der Abschlussmessung). Alle Teilnehmenden werden somit mindestens sechs Mal in die Klinik kommen.
- Direkt nach dem Einschluss in die Studie würden wir uns gerne mit Ihrem behandelnden Arzt in Verbindung setzen und über Ihre Studienteilnahme informieren. Hierzu dient die im Anschluss ausgeteilte Schweigepflichtsentbindung.
- Eine schematische Darstellung des gesamten Studienablaufes zeigt die folgende Grafik:

1) Screening-Untersuchung Dauer: 5 Stunden
- Studieninformation
- Fragebögen
- Alkoholtest
- Blutentnahme 3 Röhrchen / insgs. 11ml
  Gesundheitliche Faktoren, Alkoholmarker

ca. 9 Tage

2) Voruntersuchung Dauer: 5 Stunden
- Alkoholtest
- Fragebögen
- MRT-Untersuchung
- Blutentnahme 2 Röhrchen / insgs. 18ml
  Genetik, Epigenetik und Stammzellen

ca. 5 Tage

3) Behandlungssitzung Dauer: 8 Stunden
- Alkoholtest
- Substanzeinnahme (Placebo / Psilocybin)
- MRT-Untersuchung

1 Tag

4) Nachbesprechung Dauer: 3 Stunden
- Alkoholtest
- Nachbesprechung der Behandlungssitzung
- Blutentnahme 1 Röhrchen / insgs. 10ml
  Epigenetik

ca. 13 Tage

5) Verlaufssitzung Dauer: 3 Stunden
- Alkoholtest
- Fragebögen

ca. 14 Tage

6) Abschlussmessung Dauer: 6 Stunden
- Alkoholtest
- Fragebögen
- MRT-Untersuchung
- Blutentnahme 2 Röhrchen / insgs. 13.5ml
  Epigenetik, Alkoholmarker

3 Monate nach der Behandlungs-sitzung

7) Folge-Untersuchung Dauer: 1,5 Stunden
- Elektronische Fragebögen

6 Monate nach der Behandlungs-sitzung

8) Folge-Untersuchung II Dauer: 1,5 Stunden
- Elektronische Fragebögen

- Screening-Untersuchung (Dauer ca. 5 Stunden): 14 Tage vor der Behandlungssitzung findet eine körperliche Untersuchung inklusive elektrischer Ableitung der Herzaktivität (EKG), Blut- (3 Röhrchen, insgs. 11ml) und Urinuntersuchungen (inkl. Drogenurintest) und einer ausführlichen psychiatrischen und psychologischen Abklärung (Interview zur Biographie und zur medizinisch-psychiatrischen Vorgeschichte) statt. Blut- und Urinproben werden zu diagnostischen Zwecken verwendet und nicht für spätere Analysen aufbewahrt. Des Weiteren werden Sie angehalten, Fragebogen zu Ihrer Alkoholabhängigkeit, Persönlichkeit, Ihrem physischen und psychischen Zustand sowie zum Gebrauch psychoaktiver Substanzen auszufüllen. Vor Beginn der Studie wird bei Frauen, die schwanger werden können (s. Fussnote[1]) ein Schwangerschaftstest durchgeführt. Aufgrund dieser Daten wird entschieden, ob Sie sich für die Studie eignen und in diese aufgenommen werden können.

[1] d.h. noch nicht in der Menopause und letzte Monatsblutung vor weniger als 12 Monaten, nicht chirurgisch unterbunden, Eierstöcke und/oder Gebärmutter nicht chirurgisch entfernt

- Baseline-Untersuchung (Dauer ca. 5 Stunden): Ca. 5 Tage vor der Behandlungssitzung finden eine funktionelle Magnetresonanztomographie (fMRT) Untersuchung, eine neuropsychologische Untersuchung (MET, mehr Informationen hierzu siehe unten), ein Schwangerschafts- (bei Frauen) und Drogenurintest, sowie eine Blutentnahme (2 Röhrchen, insgs. 18ml) statt. Zudem werden Sie angehalten, Fragebogen zu Ihrer Alkoholabhängigkeit, zu Ihrem physischen und psychischen Zustand und zum Gebrauch psychoaktiver Substanzen auszufüllen.

- Behandlungssitzung (Dauer ca. 8 Stunden): Im Rahmen der Behandlungssitzung erfolgt die Substanzeinnahme (Erläuterungen siehe unten: „Substanzabgabe“). Sie werden hier nochmals ein fMRI durchlaufen, einen Schwangerschafts- (bei Frauen) und Drogenurintest abgeben und anschliessend einige Fragebögen ausfüllen.

- Nachbesprechung (Dauer ca. 3 Stunden): Am Tag nach der Behandlungssitzung findet eine Nachbesprechung statt. Hier werden Sie nochmals einige Fragebögen ausfüllen und eine Urin- und Blutprobe (1 Röhrchen, insgs. 10ml) abgeben.

- Verlaufsgespräch (Dauer ca. 3 Stunden): 14 Tage nach der Behandlungssitzung findet ein psychiatrisch-psychologisches Gespräch statt und Sie werden dazu angehalten, Fragebögen zu Ihrem physischen und psychischen Zustand auszufüllen. In diesem psychologischen Gespräch wird nochmals die Behandlungssitzung und Ihre Befindlichkeit und Ihre Bedürfnisse besprochen. Ausserdem findet eine Blutentnahme und Drogenurintest statt.

- Abschlussmessung (Dauer ca. 4 Stunden): 28 Tage nach der Behandlungssitzung findet nochmals ein Schwangerschaftsurintest, eine Drogenurinprobe, eine Blutentnahme (2 Röhrchen, insgs. 13.5ml), eine funktionelle Magnetresonanztomographie (fMRT) Untersuchung und eine neuropsychologische Untersuchung (MET) statt. Zudem werden Sie Fragebögen zu Ihrem Substanzkonsum und physischen und psychischen Zustand ausfüllen.

- Folge-Untersuchung 1 (online): 90 Tage nach der Behandlungssitzung werden Sie von uns per Mail aufgefordert, eine Batterie an Fragebogen über ein verschlüsseltes Online-Tool auszufüllen (Dauer ca. 1.5h).

- Folge-Untersuchung 2 (online): 180 Tage nach der Behandlungssitzung werden Sie von uns per Mail aufgefordert, eine Batterie an Fragebogen über ein verschlüsseltes Online-Tool auszufüllen (Dauer ca. 1.5h).

- Substanzabgabe: Je nach Gruppenzuteilung (die weder Ihnen noch dem Versuchsleiter bekannt ist) erhalten Sie im Rahmen der Behandlungssitzung das Psilocybin oder Placebo. Am Morgen vor der Behandlungssitzung sollten Sie ein leichtes Frühstück einnehmen. Die Substanz wird Ihnen vom Versuchsleiter in Form von einer Kapsel ausgehändigt. Für die gesamte Dauer der Substanzwirkung (ab Einnahme bis zur Entlassung) bleiben Sie entweder im MRT-Gebäude oder einem leicht abgedunkelten, ruhigen Raum – beide an der Psychiatrischen Universitätsklinik Zürich. Sie werden von der Versuchsleitung oder einer Krankenpflegekraft betreut. Es wird immer eine Begleitperson bei Ihnen sein und Sie durch die verschiedenen Erlebnisse während der Substanzwirkung führen. Um den Ablauf nicht zu stören, möchten wir Sie bitten, keine elektronischen Geräte wie Handys, Radiotransmitter, Laptops, Bücher usw. Mitzunehmen. Der Fokus der unter Substanzwirkung stattfindenden Gespräche liegt im Anbieten von unterstützenden, auf das innere Erleben fokussierten Anweisungen und Rückmeldungen. Zudem soll die Möglichkeit geschaffen werden, auf die Krankheitsentstehung und -aufrechterhaltung bezogene Einsichten zu gewinnen, die in den weiteren psychologischen Sitzungen vertieft werden können. Dazu werden die Gespräche auf Tonband aufgezeichnet. Falls Sie keine Substanzwirkung verspüren (weil Sie entweder das Placebo erhalten haben oder trotz Substanzeinnahme keine Wirkung verspüren) findet analog ein psychologisches Gespräch statt. Wenn keine Substanzwirkung mehr vorhanden ist (ca. 4-6 Stunden nach Einnahme) und alle Untersuchungen abgeschlossen sind, werden Sie entlassen. Wir bitten Sie, sich von einem Freund oder Familienmitglied nach Hause begleiten zu lassen und dort über Nacht unter Aufsicht zu bleiben. Sie dürfen an diesem Tag kein Fahrzeug führen oder andere Maschinen bedienen.

- Mutlifaceted Empathy Task (MET): Bei dieser Aufgabe werden Ihnen Gesichtsausdrücke gezeigt. Sie würden dann unter Anderem angeben, was die Person fühlt.

- Time-Line Follow Back Tagebuch: Sie werden vom Einschluss in die Studie bis zur sechsten Behandlungssitzung ein Tagebuch über Ihren Substanzkonsum führen. Dies werden Sie täglich zuhause ausfüllen und teilweise gemeinsam rückwirkend während der Sitzung besprechen (Voruntersuchung, Behandlungssitzung und Nachbesprechung) besprechen. Zwischen Abschlussmessung und Folge-Untersuchungen werden Sie das Tagebuch täglich zuhause ausfüllen und uns den Papierbogen monatlich in einem von uns vorbereiten und frankierten Umschlag zusenden. Die Papierbögen und Umschläge erhalten Sie and der Abschlussmessung.
- Blutuntersuchungen: Die Blutentnahmen für die molekulargenetische und (epi)genetische Analysen werden in Deutschland durchgeführt. In der molekulargenetischen Untersuchung wird der Einfluss von Psilocybin auf die DNA (das Erbgut) angeschaut.

- fMRT-Messungen: An den Messtagen (siehe Schema oben: Termin Nr. 2, 3 und 6) werden Sie im MRT-Scanner positioniert, wo eine funktionelle MagnetResonanz-Tomographie (fMRT) aufgenommen wird. Die (f)MRT ist ein etabliertes bildgebendes Standardverfahren, welches in der medizinischen Diagnostik zur Darstellung von kortikalen Reaktionen auf externe Reize im menschlichen Körper herangezogen wird. Der Scanner kommt ohne radioaktive Strahlenbelastung aus. Es basiert auf sehr starken Magnetfeldern, mit denen die Wasserstoffatome im Körper zum Schwingen angeregt werden. Diese Schwingungen können vom Gerät aufgefangen werden. Je nach Gewebeart ergibt sich ein anderes Schwingungsmuster und so entstehen strukturelle Bilder z.B. Bilder des Gehirns. Funktionelle Bilder entstehen durch die Messung des Sauerstoffverbrauchs in verschiedenen Gehirnregionen. Aktive Hirnareale weisen einen höheren Sauerstoffverbrauch auf, als weniger aktive Regionen, so dass der Zusammenhang von Struktur und Funktion analysiert werden kann. Sie werden ca. 60 Minuten im Scanner verweilen, in denen Sie gebeten werden ruhig zu liegen. Während dieser Zeit werden Sie kleinere Aufgaben lösen.

- Es kann sein, dass wir Sie von der Studie vorzeitig ausschliessen müssen. Das kann deshalb geschehen, weil Sie Ihre Einwilligung zur Studienteilnahme zurückziehen (jederzeit möglich) oder weil die Versuchsleiter die Studie abbrechen. Gründe, eine Studie abzubrechen, können beispielsweise sein, dass die Teilnehmer die Studienvorschriften nicht befolgen oder dass die weitere Studienteilnahme ein Gesundheitsrisiko für den Teilnehmer darstellt. Im Falle eines vorzeitigen Studienabbruches werden wir Ihnen anbieten, Sie zu ihrer Sicherheit abschliessend noch einmal zu untersuchen.

5. Nutzen

Es steht für Sie kein individueller Nutzen in Aussicht.

6. Rechte

Sie nehmen freiwillig teil. Wenn Sie nicht mitmachen oder später Ihre Teilnahme zurückziehen wollen, müssen Sie dies nicht begründen. Ihre medizinische Behandlung/Betreuung ist unabhängig von Ihrem Entscheid gewährleistet. Sie dürfen jederzeit Fragen zur Studienteilnahme stellen. Wenden Sie sich dazu bitte an die Person, die am Ende dieser Information genannt ist.

7. Pflichten

Wenn Sie bei der Studie mitmachen, müssen Sie bestimmte Regeln beachten. Dies ist notwendig für Ihre Sicherheit und Gesundheit. Wir werden Sie dabei so gut wir können unterstützen. Als Studienteilnehmende/r sind Sie verpflichtet:

- Ihr psychisches Befinden wahrheitsgetreu Ihrem Studienarzt oder Versuchsleitung zu berichten; insbesondere müssen Sie sich bei Verschlechterung Ihres psychischen Befindens unverzüglich mit Ihrem Studienarzt in Verbindung setzen
- Den medizinischen Anweisungen Ihres Studienarztes zu folgen und sich an den Studienplan zu halten. Es sind die spezifischen Anweisungen für den Versuch aufzuführen, z.B. am Tag der Substanzeinnahme und am Vortag keine koffeinhaltigen Getränke (Kaffee, Tee, Süssgetränke wie Cola oder Energy Drinks) und keinen Alkohol zu trinken, sowie 2 Wochen vor der Untersuchung und für die Dauer der Studie keine Drogen zu konsumieren
- Ihren Studienarzt über den Verlauf der Erkrankung zu informieren und neue Symptome, neue Beschwerden und Änderungen im Befinden zu melden;
- Innerhalb von sechs Wochen vor dem Studienstart müssen Sie ein Entzugsprogramm abgeschlossen haben;
- Ihren Studienarzt über die gleichzeitige Behandlung und Therapien bei einem anderen Arzt und über die Einnahme von Medikamenten zu informieren. Nennen Sie bitte alle Medikamente, auch solche, die Sie

selbst gekauft haben, für die Sie kein Rezept brauchen, oder auch Kräutertees, pflanzliche Arzneien etc. Sie müssen uns auch Medikamente der Alternativmedizin nennen: Homöopathie, Spagyrik, etc.

- Am Behandlungstag kein Fahrzeug zu lenken oder eine Maschine zu bedienen

Wenn Sie die Pflichten nicht beachten, können Sie Haftungsansprüche verlieren.

8. Risiken und Belastungen für die Teilnehmenden

Risiken und Belastungen durch Psilocybin:

In dieser Studie wird die halluzinogene Substanz Psilocybin als vermutlich wirksame Substanz zur Behandlung einer Depression verwendet. Psilocybin wurde in den letzten 50 Jahren im Rahmen von Forschungsprojekten bei Tausenden von Probanden untersucht und zeigte minimale Nebenwirkungen bei gleichzeitig hoher Sicherheit. Psilocybin ist körperlich sehr gut verträglich und hat ein sehr geringes Abhängigkeitsrisiko. Trotzdem können einzelne körperliche oder psychische Nebenwirkungen auftreten, die in den allermeisten Fällen vorübergehender Natur sind:

-Verstärkung oder Auslösung emotionaler Erlebnisse oder Reaktionen, welche in wenigen Fällen auch Ängste auslösen oder die Depression verstärken können. Diese Symptome können meistens durch die psychologische Betreuung während der Substanzwirkung gut beruhigt werden. Zudem können auch negative emotionale Reaktionen therapeutisch wertvoll sein und genutzt werden.

-Vorübergehende psychotische Symptome (z.B. Wahngedanken, Paranoia, Halluzinationen) wurden unter LSD-Einfluss berichtet, sind jedoch im Rahmen bisheriger Studien mit Psilocybin nicht vorgekommen. Trotzdem schliessen wir Personen mit erhöhtem Psychose-Risiko aufgrund familiärer Belastung von der Studienteilnahme aus.

-Wiederkehrende Halluzinationen nach Abklingen der akuten Substanzwirkung („flashbacks“) sind nach LSD-Konsum berichtet worden, jedoch nicht für Psilocybin. Trotzdem werden wir Risikopersonen ausschliessen (Drogenabhängigkeit, Flashbacks nach Konsum von halluzinogenen Substanzen in Vergangenheit)

-Kopfschmerzen und Erschöpfung kommen nach Psilocybin-Einnahme häufig vor. Diese sind innerhalb von 1-2 Tagen vollständig rückläufig („Katergefühl“).

-Mässige Blutdruckerhöhung und Pulsbeschleunigung sind v.a. zu Beginn der Psilocybinwirkung häufig, benötigen jedoch in den seltensten Fällen medikamentöse Gegenbehandlung.

Risiken und Belastungen durch Gegenmedikamente:

In den seltensten Fällen wird zur Behandlung der Psilocybin-Nebenwirkungen ein für die entsprechende Symptomatik zugelassenes und häufig verwendetes Gegenmedikament verabreicht. Die mit dem Gegenmedikament verbundenen Risiken und Belastungen sollen kurz genannt werden.

-Adalat retard® (im Falle starker Blutdruckerhöhung) kann Kopfschmerzen, Verstopfung, Gefässerweiterung oder Weichteilschwellungen verursachen.

-Temesta® (im Falle starker Angst oder Aufregung) kann Schläfrigkeit, Schwindel, Schwächegefühl, in seltenen Fällen Atemschwierigkeiten, Atemstillstand oder Anaphylaxie verursachen.

-Zyprexa® (im Falle von psychotischen Symptomen wie Wahn, desorganisiertem Verhalten, oder Aggressionen) kann Schläfrigkeit, Schwindel, Blutdruckabfall, Bewegungsstörungen, Mundtrockenheit, Zittern, Übelkeit und Erbrechen, sowie in seltenen Fällen Krampfanfälle, malignes neuroleptisches Syndrom und Tardive Dyskinesie verursachen.

Risiken und Belastungen durch die psychiatrischen Untersuchungen:

Es sind im Allgemeinen keine Risiken mit den Fragebögen verbunden. Allerdings kann die Besprechung und Thematisierung von Ereignissen im Leben, in der Beziehung, oder im Zusammenhang mit psychischer oder körperlicher Krankheit unangenehm oder belastend sein.

Risiken und Belastungen durch die funktionelle Magnetresonanztomographie-Untersuchung:

Bei der funktionellen Magnetresonanztomographie (fMRT)-Untersuchung handelt es sich um ein modernes, mehrfach getestetes und etabliertes Verfahren, das mit speziell dafür geeigneten Geräten durchgeführt wird und die Teilnehmer keinen gesundheitlichen Risiken aussetzt. Nach heutigen Erkenntnissen ist die fMRT-Untersuchung ohne Risiko für die menschliche Gesundheit. Bekannte Gefahren gehen nur von Metallteilen oder elektronischen Implantaten im Körper aus. Durch das technisch notwendige Magnetfeld darf man sich dem Gerät nicht mit metallenen Gegenständen nähern. Sie müssen vor der Untersuchung alle Metallgegenstände ablegen. Hierzu zählt jeglicher Körperschmuck aus Metall (Piercings, Ohrringe etc.). Bitte weisen Sie den Studienarzt oder Versuchsleitung auch auf etwaige Tätowierungen hin. Da Tätowierungen gelegentlich metallische Partikel enthalten, können sich diese während der fMRT-Untersuchung stark erhitzen, was zu Verbrennungen führen könnte. Personen mit grossflächigen Tätowierungen sind daher von der Studienteilnahme ausgeschlossen. Ausgeschlossen sind auch Personen mit Metallteilen im Körper

(Gefässclips, künstliche Herzklappen, Herzschrittmacher, Nervenstimulator, Swan Ganz Katheter, Insulinpumpe, Cochleaimplantat, Metallprothesen, auch Metallfragmente nach Geschossverletzung oder Metallsplitter im Auge, Tätigkeit in der metallverarbeitenden Industrie). Vor jeder Untersuchung wird deshalb ein fMRT-Sicherheitsscreening durchgeführt. Da die Scanner-Röhre technisch bedingt wenig Platz bietet und die Teilnehmer während der Messung in der Röhre liegen müssen, kann ein Beklemmungsgefühl bis hin zu Platzangst auftreten. Individuen mit Neigung zu Platzangst (Klaustrophobie) dürfen nicht an der Studie teilnehmen. Scanner-Geräte mit der in dieser Studie verwendeten Magnetfeldstärke (3.0 Tesla) werden in Spitälern seit vielen Jahren regelmässig für die Routinediagnostik eingesetzt und es konnten keine Folgen für die Gesundheit festgestellt werden. Vermeiden Sie dennoch rasche Bewegungen im Magnetfeld; sie können vorübergehend zu Schwindel oder metallischem Geschmack im Mund führen. Vereinzelt werden auch kurze Lichtblitze, sogenannte Phosphene, wahrgenommen. Für die eingesetzten Radiowellen gelten ähnliche Grenzwerte wie für Mobiltelefone, die bei fMRT-Untersuchungen strikt eingehalten werden. Auf diese Weise werden allfällige Effekte auf den Körper vermieden. Zum Schutz gegen die Klopfgeräusche während der Untersuchung erhalten Sie einen den arbeitsmedizinischen Vorschriften entsprechenden Gehörschutz. Sie können dennoch jederzeit per Gegensprechanlage Kontakt mit dem Personal aufnehmen.

Risiken der molekulargenetischen Untersuchungen
Bei jeder Erhebung, Speicherung und Übermittlung von Daten aus Ihren Biomaterialien im Rahmen von Forschungsprojekten bestehen Vertraulichkeitsrisiken (z.B. die Möglichkeit, Sie zu identifizieren), insbesondere im Hinblick auf die Information zu Ihrer Erbsubstanz. Diese Risiken lassen sich nicht völlig ausschließen und steigen, je mehr Daten miteinander verknüpft werden können, insbesondere auch dann, wenn Sie selbst (z.B. zur Ahnenforschung) genetische Daten im Internet veröffentlichen. Im Kapitel 11 „Vertraulichkeit der Daten und Proben“ erläutern wir Ihnen genauer, wie Ihre Privatsphäre geschützt wird. Informationen zu Ihrer Erbsubstanz können auch Bedeutung für Ihre Familienangehörigen und die Familienplanung haben.

Für Frauen, die schwanger werden können
Aufgrund von Tierversuchen wissen wir, dass Psilocybin beim ungeborenen Tier keine Schädigungen hervorruft. Die Auswirkungen von Psilocybin auf das ungeborene Kind sind aber noch nicht bekannt. Deshalb müssen Studienteilnehmerinnen während der Studie eine einfache, zuverlässige Verhütungsmethode anwenden (hormonale Pille, Intrauterinpessar, Spirale). Teilnehmerinnen, die eine chirurgische Sterilisation oder Gebärmutterentfernung durchgeführt haben, oder deren Menopause über zwei Jahre zurückliegt, müssen keine Verhütungsmethode anwenden, da sie kein Risiko für eine Schwangerschaft haben.
Teilnehmerinnen, die während der Studie schwanger werden, müssen ihren Studienarzt oder Versuchsleitung umgehend informieren und dürfen nicht weiter an der Studie teilnehmen. In diesem Fall werden Sie gebeten, Angaben über den Verlauf und den Ausgang der Schwangerschaft zu machen. Der Studienarzt wird mit Ihnen das weitere Vorgehen besprechen. Frauen, die stillen, sind von einer Studienteilnahme ausgeschlossen.

9. Andere Behandlungsmöglichkeiten
Sie müssen bei dieser Studie nicht teilnehmen. Wenn Sie nicht mitmachen, kann Ihr behandelnder Arzt auch eine medikamentöse Behandlung durchführen. In erster Linie gibt es als Behandlung für die Alkoholabhängigkeit Medikamente um Rückfälle zu reduzieren (Antabus, Acamprosat, Naltrexon, etc.).

10. Ergebnisse aus der Studie
Der Prüfarzt wird Sie während der Studie über alle neuen Erkenntnisse informieren, die den Nutzen der Studie oder Ihre Sicherheit und somit Ihr Einwilligung zur Teilnahme an der Studie beeinflussen können. Sie werden die Information mündlich und schriftlich erhalten.
Bei Zufallsbefunden (z.B. durch MRI, genetische Analysen), die bei Ihnen zur Verhinderung, Feststellung und Behandlung bestehender oder künftig zu erwartender Krankheiten beitragen können, werden Sie informiert. Wenn Sie nicht informiert werden wollen, sprechen Sie bitte mit Ihrem Prüfarzt oder Versuchsleitung.

11. Vertraulichkeit der Daten und Proben
Für diese Studie werden Ihre persönlichen und medizinischen Daten erfasst. Nur sehr wenige Fachpersonen werden Ihre unverschlüsselten Daten sehen, und zwar ausschliesslich, um Aufgaben im Rahmen der Studie zu erfüllen. Bei der Datenerhebung zu Studienzwecken werden die Daten verschlüsselt. Verschlüsselung bedeutet, dass alle Bezugsdaten, die Sie identifizieren könnten (Name, Geburtsdatum), gelöscht und durch einen Schlüssel ersetzt werden. Die Schlüssel-Liste bleibt immer in der Institution. Diejenigen Personen, die den Schlüssel nicht kennen, können daher keine Rückschlüsse auf Ihre Person ziehen. Bei einer Publikation sind die zusammengefassten Daten daher auch nicht auf Sie als Einzelperson rückverfolgbar. Ihr Name taucht niemals im Internet oder einer Publikation auf. Manchmal gibt es die Vorgabe bei einer Zeitschrift zur

Publikation, dass Einzel-Daten (sogenannte Roh-Daten) übermittelt werden müssen. Wenn Einzel-Daten übermittelt werden müssen, dann sind die Daten immer verschlüsselt und somit ebenfalls nicht zu Ihnen als Person rückverfolgbar. Alle Personen, die im Rahmen der Studie Einsicht in Ihre Daten haben, unterliegen der Schweigepflicht. Die Vorgaben des Datenschutzes werden eingehalten und Sie als teilnehmende Person haben jederzeit das Recht auf Einsicht in Ihre Daten.

Die Blutproben für die Analyse der (Epi- und Molekular)Genetik werden verschlüsselt nach Deutschland/ Mannheim versandt, dort für dieses Projekt untersucht und aufbewahrt. Falls Sie dem zustimmen, dass Ihr Blut für weitere Forschungszwecke verwendet werden darf, werden die Proben auf unbestimmte Zeit in einer Biobank gelagert. Jedoch bleibt die Schlüssel-Liste in der Institution und Zugriff haben nur autorisierte Mitarbeiter der Studie an der psychiatrischen Universitätsklinik Zürich. Der Sponsor ist dafür verantwortlich zu sorgen, dass im Ausland die gleichen Standards wie in der Schweiz eingehalten werden. Die verschlüsselten Biomaterialien und medizinischen Daten können für genauer bestimmte medizinische Forschungszwecke nach zuvor festgelegten Regeln unter Umständen auch an andere Einrichtungen wie Universitäten, Forschungsinstitute und forschende Unternehmen, ggf. auch im Ausland, weitergegeben werden. Dabei werden die Daten unter Umständen auch mit medizinischen Daten in anderen Datenbanken verknüpft, sofern die gesetzlichen Voraussetzungen hierfür erfüllt sind. Biomaterialien und Daten, die an Forscher herausgegeben wurden, dürfen nur für den vorbestimmten Forschungszweck verwendet und vom Empfänger nicht zu anderen Zwecken weitergegeben werden. Nicht verbrauchtes Material wird an die Biobank zurückgegeben oder vernichtet. Für diese Weiterverwendung bitten wir Sie, ganz am Ende dieses Dokuments eine weitere Einwilligungserklärung zu unterzeichnen.

Darüber hinaus kann es aber auch vorkommen, dass Proben und Daten an Forschungspartner in Drittländern weitergegeben werden sollen, für die keine dieser beiden Voraussetzungen erfüllt ist. Diese Länder haben möglicherweise ein niedrigeres Datenschutzniveau als die EU. Die Studienleitung sichert zu, auch in diesen Fällen die Forschungspartner vertraglich, soweit rechtlich möglich, zur Einhaltung des EU-Datenschutz-Niveaus zu verpflichten. Dennoch besteht das Risiko, dass staatliche oder private Stellen auf Ihre Daten zugreifen, obwohl dies nach dem europäischen Datenschutzrecht nicht zulässig wäre. Zudem kann es sein, dass Ihnen dort weniger oder schlechter durchsetzbare Betroffenenrechte zustehen und es keine unabhängige Aufsichtsbehörde gibt, die Sie bei der Wahrnehmung ihrer Rechte unterstützen könnte. Eine Weitergabe Ihrer Proben und Daten kann in diesem Fall nur erfolgen, wenn Sie dem ausdrücklich zugestimmt haben. Dazu können Sie in der Einwilligungserklärung das entsprechende Kästchen ankreuzen.

Die Tonbandaufnahmen der Sitzungen müssen aus rechtlichen Gründen für 10 Jahre aufbewahrt werden. Diese werden aber mit räumlicher Zugangskontrolle (Schlüssel), passwortgeschützt auf dem Server der Psychiatrischen Universitätsklinik abgelegt und nur vom Studienteam selbst ausgewertet.

Möglicherweise wird diese Studie durch die zuständige Ethikkommission oder die Arzneimittelbehörde überprüft. Der Prüfarzt muss eventuell Ihre persönlichen und medizinischen Daten für solche Kontrollen offenlegen. Ebenso kann es sein, dass bei Schäden ausnahmsweise auch ein Vertreter der Versicherung Ihre Daten ansehen muss. Alle Personen müssen absolute Vertraulichkeit wahren.

Es ist möglich, dass Ihr nachbehandelnder Arzt kontaktiert wird, um Auskunft über Ihren Gesundheitszustand zu geben.

12. Rücktritt

Sie können jederzeit aufhören und von der Studie zurücktreten, wenn Sie das wünschen. Die bis dahin erhobenen Daten und Proben werden noch verschlüsselt auswertet, weil das ganze Projekt sonst seinen Wert verliert. Nach der Auswertung werden Ihre Daten und Proben vollständig anonymisiert, d.h. Ihre Schlüsselzuordnung wird vernichtet, so dass danach niemand mehr erfahren kann, dass die Daten und Proben ursprünglich von Ihnen stammten. Eine solche Anonymisierung Ihrer Biomaterialien kann eine spätere Zuordnung des genetischen Materials zu Ihrer Person über andere Quellen allerdings niemals völlig ausschließen. Sobald die Anonymisierung erfolgt ist, ist außerdem eine gezielte Vernichtung aufgrund Ihrer Entscheidung nicht mehr möglich.

13. Entschädigung für Teilnehmende

Wenn Sie an dieser Studie teilnehmen, bekommen Sie dafür keine Entschädigung. Auslagen wie Reisespesen, die nur durch die Teilnahme bedingt sind, und Mahlzeiten die während der Studie stattfinden, werden wir Ihnen vergüten.

Es entstehen Ihnen oder Ihrer Krankenkasse keine Kosten durch die Teilnahme.

14. Haftung

Die Institution oder Firma (der Sponsor), die die Studie veranlasst hat und für die Durchführung verantwortlich ist, haftet für Schäden, welche Ihnen im Zusammenhang mit der getesteten Substanz oder Forschungshandlungen (z.B. Untersuchungen) entstehen könnten. Die Voraussetzungen und das Vorgehen dazu sind gesetzlich geregelt. Die Psychiatrische Universitätsklinik (Finanzdirektion des Kantons Zürich, Walcheplatz 1, 8090 Zürich) hat daher eine Versicherung bei der Zürich Versicherungs-Gesellschaft AG (Mythenquai 2, 8002 Zürich) abgeschlossen, um in einem möglichen Schadenfall für die Haftung aufkommen zu können. Falls Sie einen Schaden erlitten haben, so wenden Sie sich bitte an den Prüfarzt oder an das oben erwähnte Versicherungsunternehmen.

15. Finanzierung der Studie

Die Studie wird vom Schweizerischen Nationalfonds zur Förderung der wissenschaftlichen Forschung bezahlt.

16. Kontaktperson(en)

Bei Fragen, Unsicherheiten oder Notfällen, die während der Studie oder danach auftreten, können Sie sich jederzeit an eine dieser Kontaktpersonen wenden.

StudienärztInnen:
Dr. med. Raoul Bitar
Psychiatrische Universitätsklinik Zürich
Postfach 1931, 8032 Zürich
Tel.: +41 58 384 33 77
Email: raoul.bitar@puk.zh.ch

Dr. med. Christina Rossgoderer Pinto da Silva
Psychiatric Hospital, University of Zurich
Lenggstrasse 31
CH-8032 Zürich,
E-mail: christina.rossgoderer@pukzh.ch
Phone: +41 58 384 2329

Pract. med. Simon Halm
Center for Addiction Disorders
Selnaustrasse 9
CH-8001 Zürich,
Switzerland
Phone: +41 44 205 58 60
E-mail: simon.halm@pukzh.ch

Studienkoordination:
Nathalie Rieser, MSc.
Psychiatrische Universitätsklinik Zürich
Postfach 1931, 8032 Zürich
Tel.: +41 58 384 33 24
Email: nathalie.rieser@bli.uzh.ch

Leiter der Studie:
PD Dr. med. Marcus Herdener
Department of Psychiatry, Psychotherapy and Psychosomatics
Center for Addiction Disorders
Selnaustrasse 9
CH-8001 Zürich,
Switzerland
E-mail: marcus.herdener@bli.uzh.ch
Phone: +41 44 205 58 11

Notfallnummer, 24h Erreichbarkeit: 0800 33 66 55

Einwilligungserklärung

Schriftliche Einwilligungserklärung zur Teilnahme an einem Studienprojekt
Bitte lesen Sie dieses Formular sorgfältig durch. Bitte fragen Sie, wenn Sie etwas nicht verstehen oder wissen möchten. Für die Teilnahme ist Ihre schriftliche Einwilligung notwendig.

**BASEC-Nummer (nach Einreichung):**	2019-01390
**Titel der Studie (wissenschaftlich und Laiensprache)**:	Klinische und mechanistische Effekte von Psilocybin bei Patienten mit einer Alkoholabhängigkeit
**verantwortliche Institution (Sponsor mit Adresse)**:	Prof. Dr. med. Franz X. Vollenweider<br>Klinik für Psychiatrie, Psychotherapie und Psychosomatik<br>Psychiatrische Universitätsklinik Zürich<br>Neuropsychopharmakologie und Brain Imaging<br>Lenggstr. 31, Postfach 1931<br>8032 Zürich
**Ort der Durchführung**:	Klinik für Psychiatrie, Psychotherapie und Psychosomatik<br>Psychiatrische Universitätsklinik Zürich<br>Lenggstr. 31, Postfach 1931<br>8032 Zürich
**Verantwortliche Versuchsleitung am Studienort:**<br>Name und Vorname in Druckbuchstaben:	PD Dr. med. Marcus Herdener
**Teilnehmerin/Teilnehmer:**<br>Name und Vorname in Druckbuchstaben:<br><br>Geburtsdatum:	☐ weiblich ☐ männlich

- Ich wurde von der unterzeichnenden Versuchsleitung mündlich und schriftlich über den Zweck, den Ablauf der Studie mit dem Psilocybin über mögliche Vor- und Nachteile sowie über eventuelle Risiken informiert.
- Ich nehme an dieser Studie freiwillig teil und akzeptiere den Inhalt der abgegebenen schriftlichen Information. Ich hatte genügend Zeit, meine Entscheidung zu treffen.
- Meine Fragen im Zusammenhang mit der Teilnahme an dieser Studie sind mir beantwortet worden. Ich behalte die schriftliche Information und erhalte eine Kopie meiner schriftlichen Einwilligungserklärung.
- Ich wurde über mögliche andere Behandlungen und Behandlungsverfahren aufgeklärt.
- Ich bin einverstanden, dass mein behandelnder Arzt über meine Teilnahme an der Studie informiert wird. Ich bin einverstanden, dass die zuständigen Fachleute des Sponsors, der zuständigen Ethikkommission und der Arzneimittelbehörde Swissmedic zu Prüf- und Kontrollzwecken in meine unverschlüsselten Daten Einsicht nehmen dürfen, jedoch unter strikter Einhaltung der Vertraulichkeit.
- Bei Studienergebnissen oder Zufallsbefunden, die direkt meine Gesundheit betreffen, werde ich informiert. Wenn ich das nicht wünsche, informiere ich meinen Prüfarzt.
- Ich weiss, dass meine gesundheitsbezogenen und persönlichen Daten und Proben nur in verschlüsselter Form zu Forschungszwecken für diese Studie weitergegeben werden können (auch ins Ausland).
- Ich kann jederzeit und ohne Angabe von Gründen von der Studienteilnahme zurücktreten. Meine weitere medizinische Behandlung ist unabhängig von der Studienteilnahme immer gewährleistet. Die bis zum Rücktritt erhobenen Daten und Proben werden für die Auswertung zur Studie verwendet.
- Ich bin darüber informiert, dass eine Versicherung Schäden deckt, die auf die Studie zurückzuführen sind.
- Ich bin mir bewusst, dass die in der Teilnehmerinformation genannten Pflichten einzuhalten sind. Im Interesse meiner Gesundheit kann mich der Prüfarzt oder Versuchsleitung jederzeit von der Studie ausschliessen.

Ort, Datum	Unterschrift Teilnehmerin/Teilnehmer

Bestätigung der Prüfperson: Hiermit bestätige ich, dass ich dieser Teilnehmerin/ diesem Teilnehmer Wesen, Bedeutung und Tragweite der Studie erläutert habe. Ich versichere, alle im Zusammenhang mit dieser Studie stehenden Verpflichtungen gemäss dem geltenden Recht zu erfüllen. Sollte ich zu irgendeinem Zeitpunkt während der Durchführung der Studie von Aspekten erfahren, welche die Bereitschaft der Teilnehmerin/ des Teilnehmers zur Teilnahme an der Studie beeinflussen könnten, werde ich sie/ ihn umgehend darüber informieren.

Ort, Datum	Name und Vorname der Versuchsleitung in Druckbuchstaben<br><br>Unterschrift der Prüfperson

Einwilligungserklärung für Weiterverwendung von (genetischen) Daten und biologischem Material in verschlüsselter Form

**Teilnehmerin/Teilnehmer:**<br>Name und Vorname in Druckbuchstaben:		
Geburtsdatum:	☐ weiblich	☐ männlich

Ich erlaube, dass meine (genetischen) Daten und Proben aus dieser Studie für die medizinische Forschung weiterverwendet werden dürfen. Dies bedeutet, dass die Proben in einer Biobank gelagert und für zukünftige, noch nicht näher definierte Forschungsprojekte auf unbestimmte Zeitdauer verwendet werden dürfen. Diese Einwilligung gilt unbegrenzt.

Ich entscheide freiwillig und kann diesen Entscheid zu jedem Zeitpunkt wieder zurücknehmen. Wenn ich zurücktrete, werden meine (genetischen) Daten anonymisiert und meine Proben vernichtet. Ich informiere lediglich meinen Prüfarzt und muss diesen Entscheid nicht begründen.

Ich habe verstanden, dass die Daten und Proben verschlüsselt sind und der Schüssel sicher aufbewahrt wird. Die Daten und Proben können im In- und Ausland an andere Forschungsprojekte zur Analyse gesendet werden, wenn diese dieselben Standards wie in der Schweiz einhalten. Alle rechtlichen Vorgaben zum Datenschutz werden eingehalten. ☐ Ja ☐ Nein

Darüber hinaus stimme ich der Weitergabe meiner Biomaterialien und Daten in Länder außerhalb der EU auch in den Fällen zu, in denen kein Angemessenheitsbeschluss der Europäischen Kommission vorliegt und keine behördlich genehmigten Datenschutzklauseln angewendet werden. Über die möglichen Risiken einer solchen Weitergabe bin ich aufgeklärt worden (Ziff. 7c in der Information). ☐ Ja ☐ Nein

Normalerweise werden alle Daten und Proben gesamthaft ausgewertet und die Ergebnisse zusammenfassend publiziert. Sollte sich ein für meine Gesundheit wichtiges Ergebnis ergeben, ist es möglich, dass ich über meinen Prüfarzt kontaktiert werde. Wenn ich das nicht wünsche, teile ich es meinem Prüfarzt mit.

Wenn Ergebnisse aus den Daten und Proben kommerzialisiert werden, habe ich keinen Anspruch auf Anteil an der kommerziellen Nutzung.

Ort, Datum	Unterschrift Teilnehmerin/ Teilnehmer

Bestätigung der Prüfperson: Hiermit bestätige ich, dass ich dieser Teilnehmerin/ diesem Teilnehmer Wesen, Bedeutung und Tragweite der Weiterverwendung von Proben und/ oder genetischen Daten erläutert habe.

Ort, Datum	Name und Vorname der informierenden Versuchsleitung in Druckbuchstaben<br><br>Unterschrift der Prüfperson
